# Genome-Wide Identification, Characterization, and Expression Profiling of Glutathione *S*-Transferase (GST) Family in Pumpkin Reveals Likely Role in Cold-Stress Tolerance

**DOI:** 10.3390/genes9020084

**Published:** 2018-02-10

**Authors:** Md. Abdul Kayum, Ujjal Kumar Nath, Jong-In Park, Manosh Kumar Biswas, Eung Kyoo Choi, Jae-Young Song, Hoy-Taek Kim, Ill-Sup Nou

**Affiliations:** 1 Department of Horticulture, Sunchon National University, 255 Jungang-ro, Suncheon, Jeonnam 57922, Korea; kayumagb@gmail.com (M.A.K.); ujjalnath@gmail.com (U.K.N.); jipark@sunchon.ac.kr (J.-I.P.); manosh24@yahoo.com (M.K.B.); 2 Department of Genetics and Plant Breeding, Bangladesh Agricultural University, Mymensingh 2202, Bangladesh; 3 Jangchun Seed Company, 72 Sideok-ro, Yakmokmyeon, Chilgok-gun, Kyeongsangbuk-do 39821, Korea; jc@jcseed.co.kr; 4 National Institute of Biological Resources, 42, Hwangyeong-ro, Seo-gu, Incheon 22689, Korea; novaplant@naver.com; 5 University-Industry Cooperation Foundation, Sunchon National University, 255 Jungang-ro, Suncheon, Jeonnam 57922, Korea; htkim@sunchon.ac.kr

**Keywords:** cold, GST, pumpkin, genome-wide analysis, expression pattern, gene evolution

## Abstract

Plant growth and development can be adversely affected by cold stress, limiting productivity. The glutathione *S*-transferase (GST) family comprises important detoxifying enzymes, which play major roles in biotic and abiotic stress responses by reducing the oxidative damage caused by reactive oxygen species. Pumpkins (*Cucurbita* *maxima*) are widely grown, economically important, and nutritious; however, their yield can be severely affected by cold stress. The identification of putative candidate genes responsible for cold-stress tolerance, including the GST family genes, is therefore vital. For the first time, we identified 32 *C. maxima GST* (*CmaGST*) genes using a combination of bioinformatics approaches and characterized them by expression profiling. These *CmaGST* genes represent seven of the 14 known classes of plant *GSTs*, with 18 *CmaGST*s categorized into the tau class. The *CmaGST*s were distributed across 13 of pumpkin’s 20 chromosomes, with the highest numbers found on chromosomes 4 and 6. The large number of *CmaGST* genes resulted from gene duplication; 11 and 5 pairs of *CmaGST* genes were segmental- and tandem-duplicated, respectively. In addition, all *CmaGST* genes showed organ-specific expression. The expression of the putative *GST* genes in pumpkin was examined under cold stress in two lines with contrasting cold tolerance: cold-tolerant CP-1 (*C. maxima*) and cold-susceptible EP-1 (*Cucurbita moschata*). Seven genes (*CmaGSTU3*, *CmaGSTU7*, *CmaGSTU8*, *CmaGSTU9*, *CmaGSTU11*, *CmaGSTU12*, and *CmaGSTU14*) were highly expressed in the cold-tolerant line and are putative candidates for use in breeding cold-tolerant crop varieties. These results increase our understanding of the cold-stress-related functions of the GST family, as well as potentially enhancing pumpkin breeding programs.

## 1. Introduction

The glutathione *S*-transferases (GSTs, EC 2.5.1.18) are a large and complex enzyme family, involved in key metabolic steps in many eukaryotic organisms [1,2,3], including catalyzing the nucleophilic conjugation of the reduced site-specific (G-site) tri-peptide (glutathione, GSH, and Glu-Cys-Gly) into a wide range of electrophilic and hydrophobic substrates, as well as redox buffering. In plants, GSTs play vital roles in the detoxification of xenobiotics and toxic lipid peroxides [4,5], signal transduction, protection against damage from ozone, heavy metals [6], and glucosinolate biosynthesis and metabolism [7]. They also act as non-catalytic carrier proteins, which are required for vacuolar uptake of anthocyanins [8,9] and the regulation of plant growth and development [10]. The GST proteins of plants were classified into 14 distinct classes, namely tau, phi, theta, zeta, lambda, γ-subunit of the eukaryotic translation elongation factor 1B, dehydroascorbate reductase (DHAR), metaxin, tetrachlorohydroquinone dehalogenase (TCHQD), Ure2p, microsomal prostaglandin E synthase type 2 (PGES2), hemerythrin, iota, and glutathionyl-hydroquinone reductases (GHR) [11]. GST proteins in plants usually contain two domains, the GST N-domain (thioredoxin-like domain), involved in the conjugation of the GSH moiety (G-site), and the GST C-domain, which binds to hydrophobic substrates (H-site) [12]. The secondary structure analysis of the GSTs suggests that α-helix is the predominant structure followed by random coil and β-sheet in most of the plant GSTs [13].

The GSTs are directly involved in reducing oxidative damage in plants [2,14]; the overexpression of *GSTs* results in significant tolerance to oxidative stress [15,16]. The GSTs have also been suggested to be involved in controlling programmed cell death [17]. The biochemical and molecular pathways involved in the functions of GSTs in biotic and abiotic stress adaptation are the result of both catalytic and non-catalytic functions of reactive electrophile species with GSH [11]. The tau and phi GSTs are common in plants and are mainly involved in xenobiotic metabolism [18,19,20]. These GSTs were found to have broad substrate specificities in *Arabidopsis thaliana* [21] and *Populus trichocarpa* [22].The expression of the tau and phi *GSTs* is also correlated with a high tolerance to a range of abiotic stresses, including cold, dehydration, UV, oxidative stress, salt, and heavy metals [21]. In plant cells, the DHAR-class GSTs, which are particularly up-regulated during light and drought stress, catalyze the generation of the antioxidant ascorbic acid, and are therefore considered antioxidant proteins [23]. Little is known about the roles that the GHR and mPGES2 GSTs play against environmental stresses; however, *Arabidopsis* microarray data indicate they are differentially regulated under various abiotic stresses [24]. The theta GSTs putatively function to detoxify oxidized lipids [25], although GST theta 1 (GSTT1) was also found to be up-regulated in *Euphorbia esula* during drought stress and treatment with xenobiotics [26]. The zeta GSTs are involved in tyrosine catabolism [25], and were also found to be involved in enabling *E. esula* germination under chilling and salt stresses [26]. Among the GST family, a few tau, phi, and theta GSTs are known to possess glutathione peroxide activity [12]. The lambda GSTs have a putative role in flavonoid metabolism [27] and are also up-regulated during heavy metal stress [28]. 

Cold stress has a major impact on plant growth and development, causing physiological, biochemical, metabolic, and molecular changes [29]. Cold-stress tolerance can be induced in plants by acclimating them to lower temperatures over a period of time, which induces an array of cellular physical and physiochemical changes and may prevent the formation of ice within their cells, this process is called cold-acclimation. The cold-acclimation mechanism is present in many plants and agricultural crops [30]. The cold-regulated (*COR*) genes are involved in cold acclimation, and their activities are mediated by one or more effector genes and various transcription factors [30]. The *COR* genes play key roles in stabilizing membranes against freezing damage [30]. Major *cis*-acting elements, such as the LTREs (low-temperature responsive elements), ABREs (abscisic acid, ABA,-responsive elements), and DREs/CRTs (dehydration-responsive elements/C-repeats), are regulated by ABA-dependent and ABA-independent pathways in osmotic- and cold-stress responses [31]. Messengers such as ABA and reactive oxygen species (ROS) act via Ca^2+^ signaling to play a key role in inducing the expression of *COR* genes, such as *FAD8* (fatty acid desaturase 8), *ERD6* (early-responsive to dehydration 6) [30], *LOS5* (low osmotic stress 5), *FRO1* (ferric reduction oxidase 1) [32], *hsp70* (70 kDa heat shock protein) [33], and the *CBF*s (c-repeat binding factors) [34]. Most of the *COR* genes encode hydrophilic boiling-soluble polypeptides and function to promote the accumulation of secondary metabolites in the cell membrane to protect them from damage during cold stress. Under cold stress, the increased activity of anti-oxidative enzymes, such as superoxide dismutase, GST, glutathione reductase, glutathione peroxidase (GPX), ascorbate peroxidase, and catalase, as well as non-enzymatic antioxidants such as tripeptidthiol, GSH, and ascorbic acid, contribute directly to membrane stabilization [35]. 

The *Cucurbita* genus, which includes the pumpkins, squashes, and gourds, belongs to the Cucurbitaceae family. Two pumpkin species, *C.maxima* and *C. moschata*, are the most economically important cultivated *Cucurbita* crops, used as a staple food in many developing countries, although their mature fruits and seeds are consumed worldwide. The largest pumpkin producers are China, India, Russian Federation, Ukraine, United States, and Mexico [36]. *C*. *maxima* and *C*. *moschata* are used as rootstocks for other cucurbit crops, such as watermelon (*Citrullus lanatus* var. *lanatus*), cucumber (*Cucumis sativus*), and melon (multiple genera), to improve resistance against soil-borne pathogens and tolerance to abiotic stresses [37]. In our study, we identify and characterize the GST genes in *C. maxima* using a genome-wide analysis and various bioinformatics tools. We also predict which putative GSTs may be involved in cold-stress tolerance through a comparison of cold-induced gene expression data from cold-tolerant *C*. *maxima* and cold-susceptible *C*. *moschata* materials. 

## 2. Materials and Methods

### 2.1. Identification and Sequence Analysis of the CmaGSTs

The GST family members in pumpkin were identified from the cucurbit genomic database [38] and National Center for Biotechnology Information (NCBI) databases using the keyword “GST”. The cucumber (Chinese Long) *GST* gene sequences were used as queries in a Basic Local Alignment Search Tool (BLAST) search, with a cut-off E-value of <10^−10^. The coding sequence (CDS) and protein sequences of the putative GST family members were extracted from the cucurbit genomic database, and the protein sequences were further examined to confirm the presence of the GST-N (thioredoxin-like) and GST-C (hydrophobic or electrophilic binding) domains using the SMART web tool [39] and the NCBI tools [40]. In addition, the protein structures (protein length, molecular weight, and isoelectric point) were determined from the primary gene sequence using Expasy [41]. The sub-cellular localization of the identified CmaGST proteins was determined using Plant-mPLoc [42]. The GST-N domain sequences were aligned using CLUSTAL Omega [43]. The Multiple Expectation Maximization for Motif Elicitation (MEME) [44] was used to determine conserved motifs in the encoded proteins, using the following parameters: maximum number of motifs: 10; width of optimum motif ≥15 and ≤50. To determine the number of introns and exons, the CDS and genomic sequences of the *CmaGST* genes were compared using the Gene Structure Display Server (GSDS) tool [45].Putative *cis*-acting regulatory elements in the *CmaGST* genes were predicted in regions approximately 1 kb upstream of the translation initiation site (ATG) using Place [46]. 

### 2.2. Phylogenetic Analysis

The predicted CmaGST protein sequences were collected from the cucurbit genomics database [38]. *Arabidopsis thaliana*, *Oryza sativa* (rice), and *Cucumis sativus* var. *sativus* (cucumber) GST protein sequences were downloaded from TAIR [47], the MSU Rice Genome Annotation Project [48], and the Cucurbit Genome Database [38], respectively. The CmaGST protein sequences were aligned with those of *Arabidopsis*, rice, and cucumber using Clustal Omega. A phylogenetic tree was constructed in MEGA 6.0 [49] using the neighbor-joining algorithm with 1000 bootstrap replicates, using complete deletion mode to analyze tree topology and reliability. The names and sequences of all proteins used in the phylogenetic analysis are provided in Table S1.

### 2.3. Chromosome Localization and Gene Duplication Analysis

Genomic positional information for the *CmaGST* genes was collected from the cucurbit genome database, and their locations were plotted using Mapchart 2.2 [50]. Duplicated *CmaGST* genes were identified by BLAST-searching [51] them against each other, and were classed as duplicated genes when both their identity and query coverage was >80% of their partner sequence [52]. Tandem-duplicated genes were identified as an array of two or more homologous genes within a distance of 100 kb. A chromosome region containing more than two genes within 200 kb was defined as a gene cluster [53]. We estimated the synonymous rate (K_s_), non-synonymous rate (K_a_), and evolutionary constraint (K_a_/K_s_) between the duplicated *CmaGST* gene pairs based on coding sequence alignments, using the method described by Nei and Gojobori [54] for MEGA 6.0 software. The mode of selection was identified using the K_a_/K_s_ value between duplicated genes, where values >1, <1, and equal to 1 reflected positive selection, purifying selection, and neutral selection, respectively. We calculated divergence time of the duplicated gene pairs using the formula: T million year (Mya) = K_s_/2r; where T is divergence time, K_s_ is the number of synonymous substitutions per site, and r is the fixed rate of 1.5×10^−8^ synonymous substitutions per site per year expected for dicotyledonous plants [55].

### 2.4. Microsynteny of the GST Gene Family

The microsyntenic relationships of the *GST* genes in *C. maxima, C. sativus*, and *C. lanatus* subsp. *vulgaris* were detected using BLAST searches of these genes against the whole genomes of these crops. The physical location of the *CmaGST* genes on each chromosome were collected from the respective databases, and the relationships among the three crop species were plotted using Circos-0.52 [56].

### 2.5. Plant Materials, Growth, and Treatments

**Figure 1:**
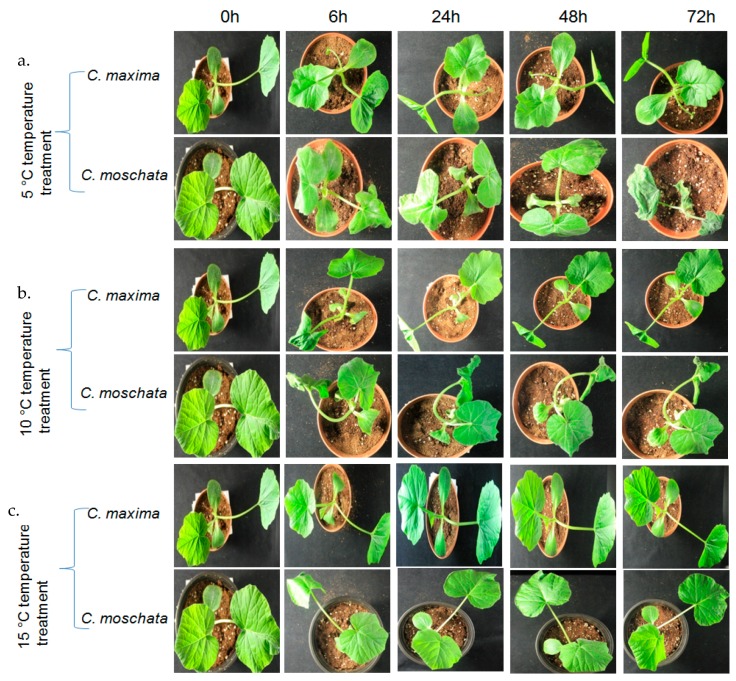
Comparison of the cold injury symptoms in cold-tolerant *Cucurbita maxima* and cold-susceptible *Cucurbita moschata* during temperature treatments; (**a**) 5 °C, (**b**) 10 °C and (**c**) 15 °C. Cold injury symptoms first appeared after 24 h in the 5 °C treated *C. moschata* plants, and gradually worsened over time. In contrast, no cold injury symptoms were observed in the *C. maxima* plants at any time point.

Two pumpkin species, moderately cold-tolerant *C. maxima* (inbred line CP-1) and cold-susceptible *C. moschata* (inbred line EP-1), were grown in a growth chamber at the department of Horticulture, Sunchon National University, Republic of Korea, at 24 °C and with a 14/10 h light/dark photoperiod. The seeds were directly sown into plastic containers containing soil. Cold stress was imposed on four-week-old seedlings, with five biological replications per treatment. The seedlings were transferred to incubators (TOGA clean system; model: TOGA UGSR01, Daejong, Korea) maintained at 5 °C, 10 °C, and 15 °C until cold injury symptoms were clearly observed on the seedlings (Figure 1a–c). Leaf samples of the cold-treated plants were excised after 0 h, 6 h, 24 h, 48 h, and 72 h of treatment. The samples were immediately frozen in liquid nitrogen and stored at −80 °C for RNA extraction. Root, stem, leaf, and flower bud samples were also collected from healthy *C. maxima* plants, to investigate the organ-specific expression of the *CmaGST*s.

### 2.6. RNA Extraction and cDNA Synthesis

Cold-treated frozen leaf samples were used for total RNA extraction with an RNeasy Mini kit (Qiagen, Hilden, Germany), following the manufacturer’s instructions. DNA was removed from the samples using RNase-free DNase (Promega, Madison, WI, USA), as instructed by the manufacturer. The extracted RNA was quantified using UV spectrophotometry at A260 NanoDropND-1000 and NanoDrop v3.7 software (Thermo Fisher Scientific, Waltham, MA, USA). The complementary DNA (cDNA) was synthesized using a First-Strand cDNA Synthesis kit (Thermo Fisher Scientific), following the manufacturer’s instructions.

### 2.7. Qualitative and Quantitative PCR Expression Analyses

Reverse transcription polymerase chain reaction (RT-PCR) qualitative expression analyses were performed with a one-step Emerald Amp GT PCR Master Mix (Takara Bio Inc., Kusatsu, Japan). *CmaGST* gene-specific primers were used for the RT-PCR, with *CmaActin* expression as an internal control (Table S2). The PCR reactions were performed using 1 µL of 50 ng cDNA from the roots, leaves, stems, and flower buds as templates, as well as a master mix containing 10 pmol each of the forward and reverse primers, 9 µL sterile water, and 8 µL Emerald Mix in a total volume of 20 µL. The PCR conditions were: an initial denaturation at 95 °C for 5 min; followed by 30 cycles of denaturation at 95 °C for 30 s, annealing at 58 °C for 30 s, and extension at 72 °C for 45 s; with a final extension at 72 °C for 5 min. The PCR amplicons were run on a 1.2% agarose gel stained with HIQ blue mango (BioD Co, Gwangmyeong, Korea) and visualized with UV.

Quantitative PCR (qPCR) was performed using a 10-μL reaction volume containing 5 μL 2× Quanti Speed SYBR mix (Thermo Fisher Scientific), 1 μL (10 pmol) each of the forward and reverse gene-specific primers (Table S1), 1 μL template cDNA (50 ng), and 2 μL distilled, deionized water. The qPCR conditions were as follows: initial denaturation at 95 °C for 5 min; followed by 40 cycles of denaturation at 95 °C for 10 s, annealing at 58 °C for 10 s, and extension at 72 °C for 15 s. Light cycler 96 SW 1.1 (Roche, Mannheim, Germany) software was used to detect amplification and process data. Fluorescence was measured at the last step of each cycle, and three replicates were used for each sample. The qPCR reactions were normalized using cucurbit *Actin* genes as a reference for all comparisons [57], and the cycle threshold (Cq)value was calculated using the 2^−ΔΔCt^ method to determine the relative expression of the genes. Heat maps were constructed based on transcript abundance value of 32 GST genes of the two contrasting lines *C. maxima* (inbred line CP-1) and *C. moschata* (inbred line EP-1) exposed to different temperature at several time courses using Cluster 3.0 and tree view software [58].

### 2.8. Statistical Analysis

One-way analyses of variance (ANOVAs) were performed with Minitab 18 (State College, PA, USA) statistical software to detect significant differences among the relative expression levels of the genes. The mean separation of the relative expression values was determined using Tukey’s pair-wise comparison test.

## 3. Results

### 3.1. Identification of GST Genes in C. maxima

We identified GST genes from the cucurbit and NCBI databases using the key word “GST”. The *C. sativus* gene sequences were BLAST-searched against the *C. maxima* genome to obtain the putative *CmaGST* genes, and a series of systematic analyses were performed to assemble the final set of 32 *C. maxima* GST gene sequences (Table 1). The protein and genomic sequences for these candidates were obtained from the cucurbit database [38]. The CmaGST protein sequences were highly similar to those of other plant species, and all CmaGST proteins contained both the GST-N and GST-C domains. Certain proteins contained other specific domains; the EF1G (elongation factor 1γ, pfam00647) was present in CmaEF1G1, CmaEF1G2, and CmaEF1G3, while the UCH (ubiquitin carboxyl terminal hydrolase) and RPT1 (internal repeat 1) domains were found in CmaGSTU16 and CmaGSTU7, respectively. The GST-N domain was comprised of βαβαββα motifs, resulting in three layers of β-sheets sandwiched by three layers of α-helixes (Figure S1). The N-terminus and C-terminus were connected by a small peptide sequence (8–15 aa) called the linker region in all CmaGSTs. The predicted isoelectric points (5.17–9.38), molecular weights (23.19–91.29 KDa), sub-cellular localizations, and residual sizes (202–810 aa) of the 32 putative CmaGST proteins are presented in Table 1.

### 3.2. Phylogenetic Analysis

**Figure 2:**
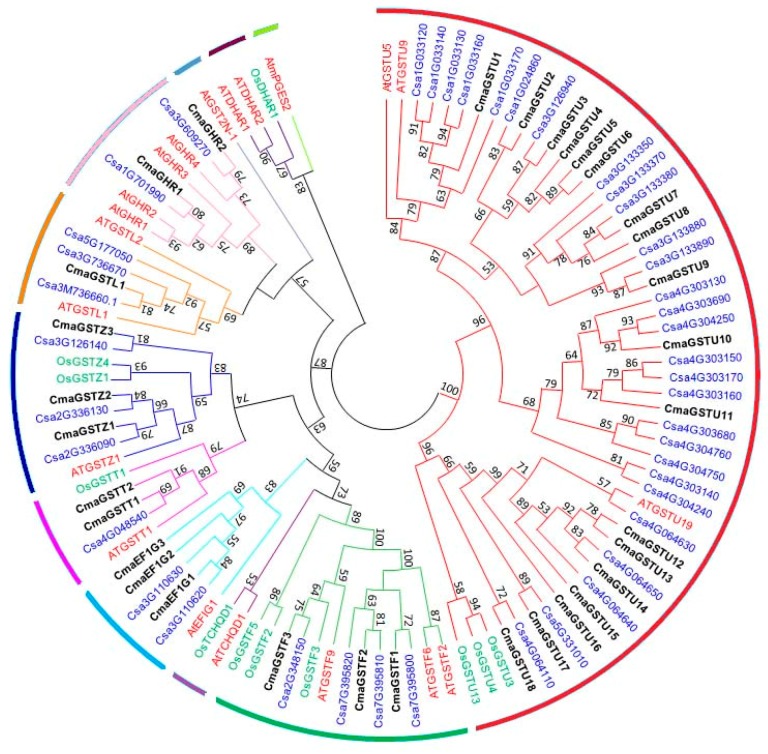
Phylogenetic analysis of full-length glutathione *S*-transferase (GST) proteins from *C.maxima*, *Cucumis sativus*, *Arabidopsis thaliana*, and *Oryza sativa*. This phylogenetic tree was generated using the neighbor-joining method in MEGA 6.0, with 1000 bootstrap replicates. Black, blue, red, and green text indicates *C. maxima*, *C. sativus*, *A. thaliana*, and *O. sativa* proteins, respectively.

A phylogenic tree was constructed using 106 full-length GST protein sequences from *Arabidopsis* (20 sequences), rice (11 sequences), cucumber (43 sequences), and the 32 CmaGST proteins (Figure 2), to investigate the evolutionary relationships of the CmaGST proteins. We have found 11 classes of GSTs out of recently published 14 classes [11], eight of which (tau, phi, zeta, theta, lambda, EF1G, DHAR, and TCHQD) correspond to the *Arabidopsis GST* gene classification by Dixon and Edwards [12], while the remaining three classes (GHR, mPGES-2, and GST2N) were identified by Vijayakumar et al. [59]. A total of 18 CmaGSTs were attributed to the tau class, the largest and most abundant group, while three CmaGSTs each were positioned in the phi, EF1G, and zeta classes (Figure 2). In addition, two CmaGSTs were categorized as theta GSTs, while another two were grouped into the GHR category. The remaining CmaGST was located in the lambda class. No CmaGST proteins were attributed to the TCHQD, GST2N, DHAR, and mPGES2 classes. 

### 3.3. Chromosomal Locations and Gene Duplications of the CmaGSTs

**Figure 3:**
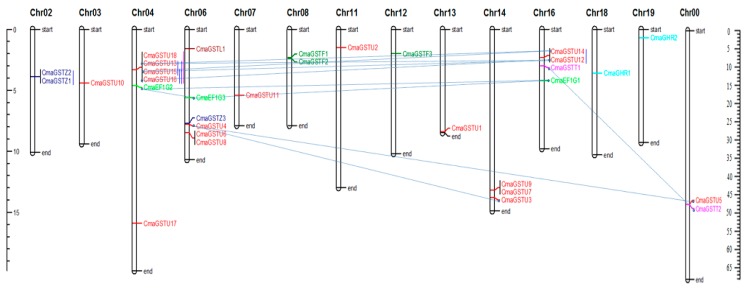
Chromosomal distribution and gene duplication of the *GST* genes in *C. maxima*. The chromosome number is indicated at the top of each chromosome; Chr00 is the gene scaffold. The scales on the left and right sides (in Mb) are for the chromosomes and scaffold, respectively. The pink and blue bars represent *GST* gene clusters and tandem duplication, respectively. The segmentally duplicated gene pairs are connected with dotted light blue lines. Different classes of *GST* genes have different text colors; red, green, light green, blue, pink, paste and brown colours represent tau, phi, EF1G, zeta, theta, GHR and lamda group of *GST* genes, respectively.

The distribution of the *CmaGST* genes across the pumpkin chromosomes was mapped, showing that most were located on relatively few chromosomes, including chromosomes 4, 6, 14, and 16 (Figure 3). Six *CmaGST* genes each were found on chromosomes 4 and 6, while chromosomes 3, 7, 11, 12, 13, 18, and 19 each contained only one *CmaGST* gene. The distribution of the *CmaGST* genes in the pumpkin genome reflected the diversity and complexity of this gene family. A cluster of tau *CmaGST* genes was identified on chromosome 4 (Figure 3). In other species, the genomic locations of the GST gene family are known to be influenced by genetic events including segmental duplication, tandem duplication, and polyploidization [60,61]. We identified 11 pairs of segmentally duplicated and five pairs of tandem-duplicated GST genes in the pumpkin genome (Figure 3, Table 2). Moreover, the substitution rate of non-synonymous (K_a_) and synonymous (K_s_) mutations was estimated to assess the selection pressures and divergence time among the duplicated *CmaGST* gene pairs (Table 2). K_a_/K_s_ values <1, 1, and >1 indicate negative or purifying selection, neutral selection, and positive selection, respectively. Among the 16 *CmaGST* duplicated gene pairs, 15 had K_a_/K_s_ values below 1, indicating that these duplicated genes evolved under purifying selection pressure in *C. maxima* (Table 2). Only one pair of duplicated genes had a K_a_/K_s_ value above 1, suggesting that this gene pair evolved under strong positive selection pressures in *C. maxima*. We also calculated the divergence time of the *CmaGST* genes, revealing that the duplication events began 18.87 Mya and continued up until 1.30 Mya (Table 2). The N-terminal GST domain sequence similarity of the CmaGST proteins was greater within classes than between classes (Figure S1); 11 pairs of tau CmaGST proteins shared more than 80% similarity, indicating the high rate of gene duplication within this class. In addition, four conserved regions and the G-site were found in all CmaGST proteins (Figure S1).

### 3.4. Structural and Motif Analyses of the CmaGSTs

We analyzed the exon-intron structures of the *CmaGST* genes using the GSDS online tool, revealing different numbers of exons and introns in different classes of these genes. The tau-class *CmaGST*s typically contained two exons, except that only one exon was present in *CmaGSTU1*, three exons were present in *CmaGSTU2* and *CmaGSTU5*, four exons were found in *CmaGSTU3*, and *CmaGSTU16* contained 10 exons (Figure S2). All of the genes in the phi class contained three exons, whereas the EF1G class contained six to nine exons. The zeta class genes contained 8–10 very small exons, while the two GHR-class genes contained three or six exons. The lambda (*GSTL1*) and theta (*GSTT1* and *GSTT2*) genes possessed nine and seven exons, respectively (Figure S2). 

Conserved motifs were found among the GST genes of *C. maxima* using the MEME web tool. Motifs 3 and 4 were present in all classes except the EF1G and lambda classes, respectively (Figure S3). The lambda-class gene contained only one motif (motif 3), while motifs 8 and 10 were unique to the EF1G class. Motifs 1 and 9 were unique to the tau class genes, while motifs 5 and 9 were only present in certain tau class members. Motif 2 was present in the tau and zeta class genes, but motif 6 was absent in the genes of the tau, lambda, and GHR classes. The phi and theta class genes contained motifs 3, 4, and 6, but in the theta class genes, motif 4 was partially or completely absent (Figure S3).

### 3.5. Microsynteny Relationships and Analysis of CmaGST Protein Interactions

**Figure 4:**
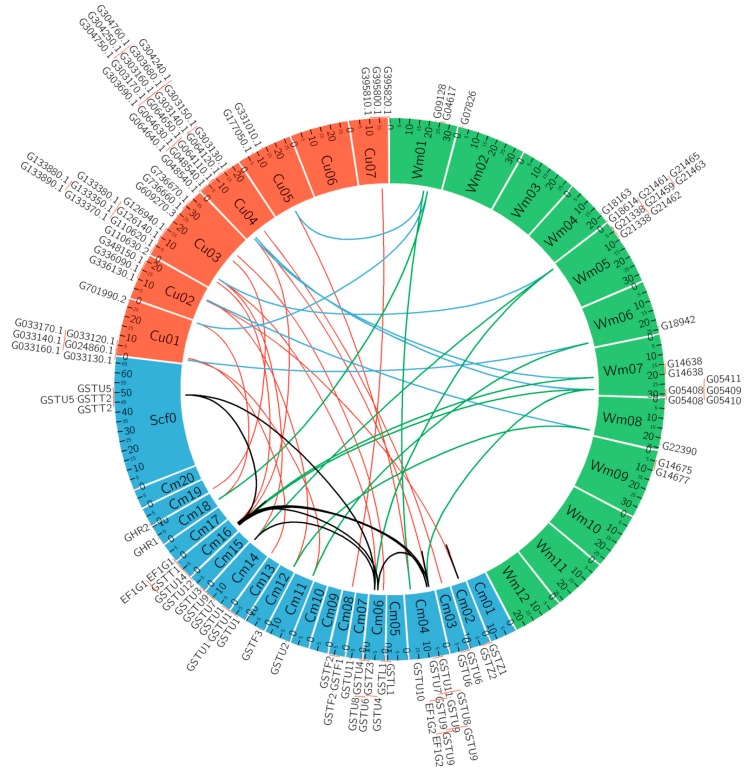
Microsynteny analyses of GST genes in *C. maxima* (Cm), *C. sativus* (Cu), and *Citrullus lanatus* var. *lanatus* (Wm). Light blue, orange, and green blocks represent *C. maxima*, *C. sativus*, and *C.lanatus* var. *lanatus* chromosomes, respectively. Black lines denote duplicated *CmaGST* genes on the *C. maxima* chromosomes.

A microsynteny map was constructed using orthologous GST gene pairs among *C. maxima*, *C. sativus*, and *C. lanatus* subsp. *vulgaris* to explore their evolutionary history and relationships (Figure 4). Nine orthologous gene pairs were identified between *C. maxima* and *C. lanatus* subsp. *lanatus*, whereas 24 orthologous gene pairs were identified between *C. maxima* and *C. sativus* (Figure 4). This indicates that *CmaGST* genes are more closely related to those of *C. sativus* than *C. lanatus*. This study also provided further evidence of the 16 pairs of duplicated *CmaGST* genes.

**Figure 5:**
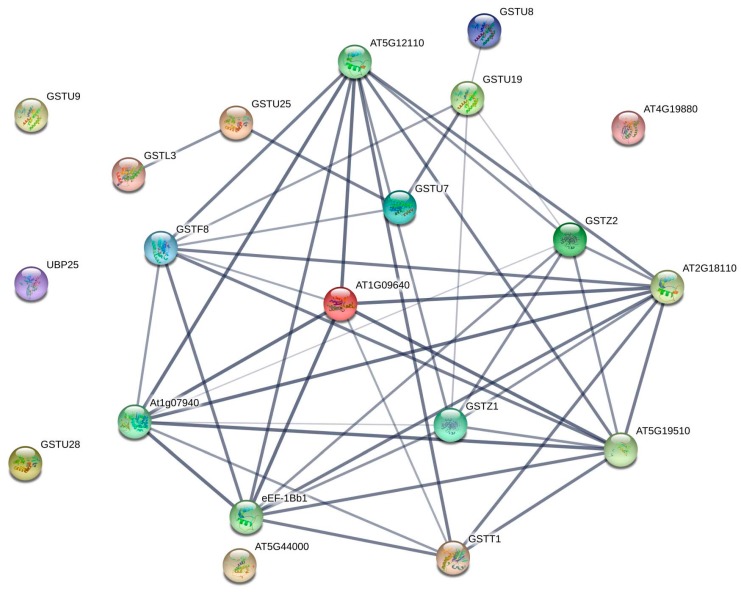
Interaction networks of the 32 CmaGST proteins based on their homology to *Arabidopsis thaliana* GST proteins. Stronger associations are represented by thicker lines.

We used STRING 10.5 version software to predict the interactions of the 32 *C. maxima* GST proteins based on their homology to *Arabidopsis* proteins, to identify their putative functional and physical roles (Figure 5). CmaGSTU2, CmaGSTU7, CmaGSTU8, and CmaGSTU9 showed high homology to AtGSTU7, which is involved in cold stress [62]. AtGSTU7 is also closely related to AtGSTU19 and AtGSTU25, which are associated with the response to chilling, salicylic acid, and H_2_O_2_ stress. The phi (CmaGSTF1, CmaGSTF2, and CmaGSTF1), EF1G (CmaEF1G1, CmaEF1G2, and CmaEF1G3), zeta (CmaGSTZ1 and CmaGSTZ2), and theta (CmaGSTT1 and CmaGSTT2) proteins are highly homologous to *Arabidopsis* AtGSTF8, AT1G09640, AtGSTZ1, and AtGSTT1, respectively, which are associated with responses to a range of stresses and signals, including H_2_O_2_, salicylic acid, and bacterial pathogens. *Arabidopsis* proteins AtGSTU19, AtGSTU25, AtGSTL3, At5G12110, At1G07940, AteEF-1Bb1, AT5G19510, AT2G18110, and AtGSTZ2 are also involved in the protein interaction network (Figure 5).

### 3.6. Expression Profiles of CmaGST Genes in Various Organs

**Figure 6:**
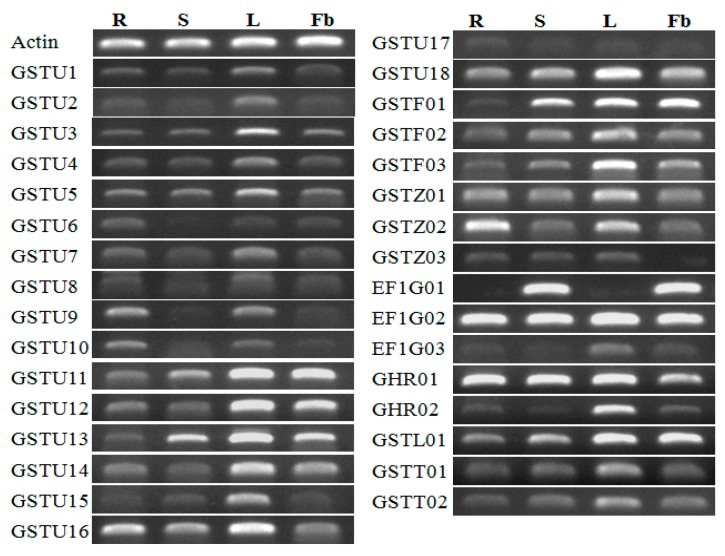
Expression patterns of the *CmaGST* genes in various tissues, determined by reverse transcript polymerase chain reaction (RT-PCR) analysis. From left to right, each band shows the amplified products from the roots (R); stems (S), leaves (L); and flower buds (Fb).

We performed RT-PCR to analyze the expression patterns of the *CmaGST* genes in different organs (root, stem, leaf, and flower buds) of healthy *C. maxima* plants. Most of the *CmaGST* genes were abundantly expressed in all tested organs (Figure 6), although *CmaGSTU17* was weakly expressed in all tested organs. *CmaGSTU15*, *CmaEF1G03*, and *CmaGHR02* were highly expressed in the leaf relative to the other organs. *CmaGSTZ03* was expressed in all organs except the flower buds, while *CmaEF1G01* expression was absent from the root and leaf. *CmaGSTU6* and *CmaGSTU9* were expressed in the root, leaf, and flower buds, but not in the stem. Moreover, *CmaGSTU2*, *CmaGSTU8*, and *CmaGSTU10* were expressed in all organs but only very weakly in the stem and flower buds (Figure 6).

### 3.7. Expression Profiling of the CmaGST Genes during Cold Treatment

**Figure 7:**
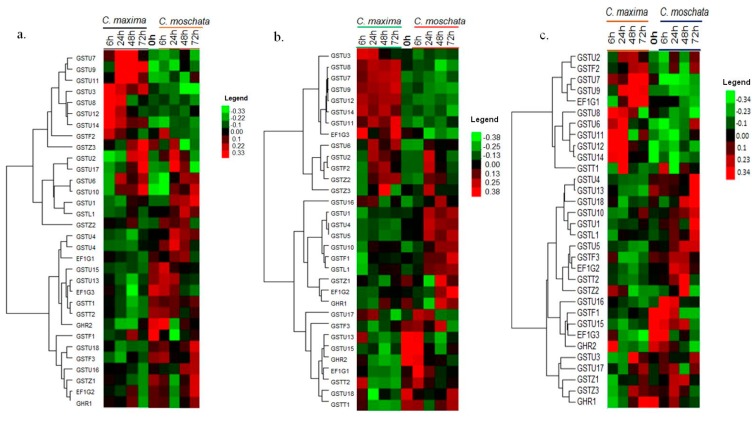
Differential expression profiles of 32 *CmaGST* genes in two contrasting lines *C. maxima* (inbred line CP-1) and *C. moschata* (inbred line EP-1) exposed to different temperatures at different time courses:(**a**) at 5 °C; (**b**) at 10 °C; and (**c**) at 15 °C. The gene name against each expression has mentioned at left side and color bars with values at right side represent differential expression, where red and green color represent up- and down-regulations of the genes, respectively.

Moderately cold-tolerant *C. maxima* and cold-susceptible *C. moschata* lines were treated with different temperatures (5 °C, 10 °C, and 15 °C) to elucidate the expression patterns of the *CmaGST* genes in response to cold stress. A qPCR analysis revealed that the majority of *CmaGST* genes were down-regulated in *C. maxima* during the 5 °C treatment (Figure 7a, and Figures S4a and S5a); however, *CmaGSTU3*, *CmaGSTU8*, *CmaGSTU12*,and *CmaGSTU14* had 8-, 6-, 11-, and 8-fold higher expressions, respectively, after 6 h. *CmaGSTU7*, *CmaGSTU9*,and *CmaGSTU11* exhibited 4-, 5-, and 2-fold higher expressions, respectively, in *C. maxima* compared with *C. moschata* after 24 h at 5 °C. *CmaGSTU10*, *CmaGSTU17*, and *CmaGSTZ2* were up-regulated in both species after 24 h at 5 °C (Figure 7a and Figure S4a,b); however, the majority of the *CmaGST* genes were down-regulated (Figure S5b). In contrast, *CmaGSTU1*, *CmaGSTU4*, *CmaGSTU5*, and *CmaGSTL1* showed 16-, 15-, 14-, and 5-fold higher expression levels, respectively, in *C. moschata* compared with *C. maxima* after 24 h of treatment at 5 °C; however, their expression levels declined over time (Figure 7a and Figure S4b)

A total of nine *CmaGST* genes showed significantly higher expression levels in *C. maxima* compared with *C. moschata* after 6 h at 10 °C. Among them, *CmaGSTU3*, *CmaGSTU7*, *CmaGSTU8*, *CmaGSTU9*, *CmaGSTU11*, *CmaGSTU12*, and *CmaGSTU14* had more than a 10-fold higher expression in the cold-tolerant line than the susceptible line. Four genes, *CmaGSTU7*, *CmaGSTU8*, *CmaGSTU9*, and *CmaGSTU11,* exhibited 10-, 12-, 14-, and 10-fold higher levels of expression, respectively, in *C. maxima* than in *C. moschata* after 24 h at 10 °C (Figure 7b and Figure S4c,d). In the cold-susceptible line, the expression levels of *CmaGSTU1*, *CmaGSTU4*, *CmaGSTU5*, and *CmaGSTL1* were 22-, 30-, 28-, and 6-fold higher than the cold-resistant cultivar at 24 h and 48 h (Figure S4d). The rest of the genes did not have significantly different levels of expression at any time point in *C. maxima* or *C. moschata*, except for the *EF1G3* and *GSTT2* genes at the 6 h time point (Figure S5c,d).

During the 15 °C temperature treatment, seven genes were up-regulated in the cold-tolerant line compared with the susceptible line, with expression changes of 6-fold at 6 h for *CmaGST6*, 12-fold at 24 h for *CmaGST7*, 10-fold at 6 h for *CmaGST8*, 4-fold at 24 h for *CmaGST9*, 14-fold at 6 h for *CmaGST11*, 8-fold at 24 h for *CmaGST12*, and 7-fold at 24 h for *CmaGST14* (Figure 7c and Figure S4e). By contrast, seven *CmaGST* genes (*CmaGSTU1*, *CmaGSTU4*, *CmaGSTU5*, *CmaGSTU10*, *CmaGSTF2*, *CmaGSTZ2*, and *CmaGSTL1*) in the susceptible line were significantly up-regulated at the 48 h time point and drastically decreased at the 72 h time point in *C. moschata* (Figure 7c and Figure S4f). After 48 h at 15 °C, the majority of genes were up-regulated in *C. moschata* compared with *C. maxima*, although some genes showed very low levels of expression or did not have a significant response to the 15 °C treatment in *C. maxima* and *C. moschata* (Figure 7c and Figure S5e,f).

## 4. Discussion

The *GST* genes play key roles in plant growth and stress responses [59,63,64,65]. Plants have a high number of GSTs categorized into 14 classes [11], which can have constitutive or tissue-specific expression, or be induced by biotic and abiotic stresses. In our study, 32 full-length *GST* genes were identified in pumpkin and evaluated through various in silico approaches. The phylogenetic analysis revealed high level of similarities within the same classes of GSTs in four plant species, pumpkin, cucumber, *Arabidopsis*, and rice (Figure 2), which indicates that these classes evolved before the monocot–dicot divergence. Pumpkin has more *GST* genes than soybean (*Glycine max*) [66], but fewer than *Arabidopsis* [62], rice [67], sweet orange (*Citrus sinensis*) [68] and maize (*Zea mays*) [66]. Gene family expansion in plants often arises as a result of tandem duplications, segmental duplications, whole-genome duplications, and inter-specific hybridizations, and can facilitate the evolution of functional diversity [69]. We identified seven pairs of segmental- and four pairs of tandem-duplicated tau-class genes, representing 44% of the tau GSTs (Figure 3 and Table 2). Only one duplicated gene pair was found in each of the theta and zeta classes, whereas the EF1G class contained three gene pairs that evolved by segmental duplication (100% of EF1G-class genes). We calculated the K_a_, K_s_, and K_a_/K_s_ values of the duplicated gene pairs, providing an estimate of their selection history. Fifteen of the 16 duplicated *CmaGST* gene pairs had K_a_/K_s_ values less than 0.8 (Table 2), suggesting that majority of gene pairs evolved through purifying selection. 

In the protein association networks, CmaGSTU2, CmaGSTU7, CmaGSTU8, and CmaGSTU9 showed similarity to GSTU7 in *Arabidopsis* (Figure 5), which is involved in the response to abiotic (chilling and hypoxia) and biotic (fungal and bacterial) stresses [62,70]. CmaGSTU14, CmaGSTU15, and CmaGSTU16 were predicted to have similar functions to AtGSTU19, while CmaGSTU12 and CmaGSTU17 were associated with AtGSTU25, and were predicted to have a strong interaction with the homologs of AtGSTU7. From these results, we speculated that nine CmaGSTs (CmaGSTU2, CmaGSTU7, CmaGSTU8, CmaGSTU9, CmaGSTU12, CmaGSTU13, CmaGSTU15, CmaGSTU16, and CmaGSTU17) might be involved in the response to cold, fungal, and bacterial stresses. By contrast, CmaGSTF1, CmaGSTF2, and CmaGSTF3 were more functionally related to AtGSTF8 (Figure 5), which also has functions in the response to chilling, bacterial, and fungal stresses [3,62]. Theta and zeta classes of GSTs have glutathione peroxidase activity [4] and the ability to reduce cytotoxic hydroperoxides. Glutathione reductase reduces glutathione levels by catalyzing the glutathione disulfide into the sulfhydryl [71], providing a substrate for the GSTs. They play a role in the cellular detoxification process through facilitating a conjugation reaction; and are also associated with other cellular proteins or pathways involved in detoxification.

All *CmaGST* genes were differentially expressed in different organs (Figure 6). Only one gene (*CmaEF1G01*) was expressed exclusively in the stem and flower buds. In contrast, *CmaGSTU6* and *CmaGSTU9* were not expressed in the stem and *CmaGSTZ03* was not expressed in the flower buds, suggesting that they might not be responsible for the development of these respective organs (Figure 6). The rest of the GST genes were expressed in all tested organs, consistent with the organ-specific microarray expression data of the *AtGSTs* [12], tomato (*Solanum lycopersicum*) *GSTs* [63], and *OsGSTs* [67].

The *CmaGST*s were also differentially expressed in cold-tolerant and cold-susceptible lines following treatments with various cold temperatures. Seven genes (*CmaGSTU3*, *CmaGSTU7*, *CmaGSTU8*, *CmaGSTU9*, *CmaGSTU11*, *CmaGSTU12*, and *CmaGSTU14*) were up-regulated in the cold-tolerant line compared with the cold-susceptible line after 6 h or 24 h of treatment with cold temperatures (Figure 7a–c) and might therefore be putative candidates for breeding cold-tolerant pumpkins. Similarly, in cold-stress treatments in cabbage (*Brassica oleracea*), *BoGSTU19*, *BoGSTU24*, and *BoGSTF10* were identified as putative candidate genes involved in cold-stress tolerance, as they were more highly expressed in the cold-tolerant line (Bo106) than the cold-susceptible line (Bo107) [59]. Sappl et al. [62] reported that *AtGSTU7* was significantly up-regulated after 3 h of chilling stress. Dalton et al. [72] reported that the *GSTs* are involved in physiological flexibility and resistance to various biotic and abiotic stresses in plants. The seven putative candidate *CamGST* genes involved in the cold response contained highly cold stress responsive *cis*-acting elements (Table S3). Plant cells sense cold stress via membrane rigidification, as well as through changes metabolite concentration, initiating the primary signals, such as ABA, Ca^2+^, nitric oxide (NO) [73], and ROS, to induce a variety of responses, like stomatal closure. The GST and GPX proteins are known to be highly induced during ROS formation, enabling the detoxification of lipid peroxides and DNA degradation products, and removing ROS. During cold stress, the cellular levels of ROS increase and act as a secondary messengers, inducing programmed cell death [74]; however, ROS also induce the MAPKK (mitogen-activated protein kinase kinase) proteins, which can activate defensive genes to induce conjugation processes, including the processing of GSH by GST enzymes, which becomes localized in the vacuole by transporters (ABC transporter cascade) for degradation. 

*CmaGSTU2*, *CmaGSTU6*, *CmaGSTU10*, *CmaGSTU17*, *CmaGSTF2*, and *CmaGSTZ2* expression was up-regulated by the cold treatments in both the cold-tolerant and -susceptible lines (Figure 7a–c), suggesting that these six genes are not affected by any internal variable factors. Four *GST* genes (*CmaGSTU1*, *CmaGSTU4*, *CmaGSTU5*, and *CmaGSTL1*) were highly expressed in the cold-susceptible line than the cold-tolerant one, which might result in the cold susceptibility of this plant (Figure 7a–c); therefore, these genes might be the putative candidates for developing cold-tolerant pumpkin cultivars via gene editing/anti-sense technique or molecular genetics technologies. These genes might play differential roles in the signal transduction pathways and/or cooperate with other genes to form networks that defend plants against adverse environmental conditions.

## 5. Conclusions

In conclusion, this is the first report of the genome-wide identification, characterization, and expression profiling of the GSTs in pumpkin. We systematically analyzed the *C. maxima* genome, identified 32 GSTs, and characterized those using bioinformatics and expression analyses in response to cold (5 °C, 10 °C, and 15 °C) stresses. Seven (*CmaGSTU3*, *CmaGSTU7*, *CmaGSTU8*, *CmaGSTU9*, *CmaGSTU11*, *CmaGSTU12,* and *CmaGSTU14*) of the 32 GST genes were more highly expressed in the cold-tolerant cultivar (*C. maxima*) than in the cold-susceptible cultivar (*C. moschata*) during cold stress and might therefore be useful for developing cold-tolerant cultivars through conventional, molecular, or transgenic breeding approaches. The comprehensive expression analysis in response to cold stress in pumpkin provided novel information regarding the cold-related physiological roles of the *CmaGST* genes, which could enhance the selection of potential genes utilized for marker-assisted breeding and/or the engineering of transgenic plants with increased cold tolerance in future research.

## Figures and Tables

**Table 1:** In silico analysis and sequence characteristics of the 32 putative glutathione *S*-transferase (GST) genes identified in *Cucurbita maxima*.

Sl. No.	Gene Name	Accession No.	Chromosome	Start Location	End Location	Strand	pI	MW(kDa)	AA	Localization
01	*CmaGSTU1*	CmaCh13G011300	13	8381645	8382352	+	5.74	27.04	235	Cytoplasm
02	*CmaGSTU2*	CmaCh11G002990	11	1483599	1484338	+	5.17	25.08	219	Cytoplasm
03	*CmaGSTU3*	CmaCh14G019610	14	13801828	13805918	+	6.31	44.50	387	Cytoplasm
04	*CmaGSTU4*	CmaCh06G011550	06	7777316	7778827	−	5.93	26.16	227	Cytoplasm
05	*CmaGSTU5*	CmaCh00G006890	00	47643604	47645087	−	6.98	27.96	242	Cytoplasm
06	*CmaGSTU6*	CmaCh06G012770	06	8499592	8500393	−	6.02	26.29	226	Cytoplasm
07	*CmaGSTU7*	CmaCh14G018620	14	13214607	13217686	−	5.98	41.95	370	Cytoplasm
08	*CmaGSTU8*	CmaCh06G012780	06	8501349	8502406	+	6.62	25.61	222	Cytoplasm
09	*CmaGSTU9*	CmaCh14G018610	14	13213235	13214322	−	5.28	25.41	222	Cytoplasm
10	*CmaGSTU10*	CmaCh03G003840	03	4366133	4366923	+	5.94	24.72	217	Cytoplasm
11	*CmaGSTU11*	CmaCh07G010310	07	5378533	5380070	−	6.20	25.66	220	Cytoplasm
12	*CmaGSTU12*	CmaCh16G004650	16	2333498	2334374	+	6.14	24.95	219	Cytoplasm
13	*CmaGSTU13*	CmaCh04G006390	04	3269916	3271375	−	8.63	33.08	288	Cytoplasm
14	*CmaGSTU14*	CmaCh16G004640	16	2326120	2328293	+	7.66	23.19	202	Cytoplasm
15	*CmaGSTU15*	CmaCh04G006400	04	3271577	3272321	−	5.27	24.41	212	Cytoplasm
16	*CmaGSTU16*	CmaCh04G006410	04	3273122	3286431	−	9.02	91.29	810	Nucleus
17	*CmaGSTU17*	CmaCh04G022700	04	15857294	15858045	−	5.58	25.47	224	Cytoplasm
18	*CmaGSTU18*	CmaCh04G006380	04	3268691	3269778	−	5.65	25.65	224	Cytoplasm
19	*CmaGSTF1*	CmaCh08G004090	08	2344840	2346569	+	6.38	24.14	217	Cytoplasm
20	*CmaGSTF2*	CmaCh08G004100	08	2346863	2348162	−	5.75	24.12	214	Cytoplasm
21	*CmaGSTF3*	CmaCh12G003850	12	1988809	1990016	−	5.51	25.09	220	Cytoplasm
22	*CmaEF1G1*	CmaCh16G007670.	16	4206363	4213994	+	5.54	61.96	547	Cytoplasm
23	*CmaEF1G2*	CmaCh04G008920	04	4645271	4652681	+	5.65	60.31	531	Cytoplasm
24	*CmaEF1G3*	CmaCh06G008870	06	5603706	5606718	−	6.01	47.14	415	Cytoplasm
25	*CmaGSTT1*	CmaCh16G005900	16	3054792	3058033	−	9.38	27.09	238	Cytoplasm
26	*CmaGSTT2*	CmaCh04G007350	00	47643604	47645087	+	9.34	26.96	238	Cytoplasm
27	*CmaGSTZ1*	CmaCh02G006430	02	3895323	3899120	+	5.85	24.89	222	Cytoplasm
28	*CmaGSTZ2*	CmaCh02G006410	02	3887013	3892301	+	5.17	24.99	223	Cytoplasm
29	*CmaGSTZ3*	CmaCh06G011350	06	7652425	7656514	+	5.20	25.63	228	Cytoplasm
30	*CmaGSTL1*	CmaCh06G003420	06	1588222	1591488	−	5.25	29.35	257	Cytoplasm
31	*CmaGHR1*	CmaCh18G005590	18	3671672	3674403	−	7.62	40.83	355	Chloroplast
32	*CmaGHR2*	CmaCh19G001140	19	681203	684746	+	8.79	45.58	415	Cytoplasm

pI: isolectric point; MW: molecular weight; aa: amino acids.

**Table 2:** Estimated K_a_/K_s_ ratios and divergence times of the duplicated *CmaGST* genes.

Duplicated Gene Pairs			K_s_	K_a_	K_a_/K_s_	Duplication Type	Type of Selection	Divergence Time (Mya)
*CmaGSTU3*	vs.	*CmaGSTU4*	0.215	0.127	0.593	Segmental	Purifying	7.18
*CmaGSTU4*	vs.	*CmaGSTU5*	0.139	0.056	0.405	Segmental	Purifying	4.66
*CmaGSTU12*	vs.	*CmaGSTU14*	0.038	0.017	0.452	Tandem	Purifying	1.30
*CmaGSTU12*	vs.	*CmaGSTU13*	0.412	0.142	0.346	Segmental	Purifying	13.75
*CmaGSTU12*	vs.	*CmaGSTU15*	0.455	0.137	0.302	Segmental	Purifying	15.19
*CmaGSTU12*	vs.	*CmaGSTU16*	0.533	0.229	0.430	Segmental	Purifying	17.78
*CmaGSTU13*	vs.	*CmaGSTU14*	0.387	0.142	0.367	Segmental	Purifying	12.91
*CmaGSTU13*	vs.	*CmaGSTU15*	0.405	0.172	0.424	Tandem	Purifying	13.52
*CmaGSTU13*	vs.	*CmaGSTU16*	0.309	0.242	0.784	Tandem	Purifying	10.32
*CmaGSTU14*	vs.	*CmaGSTU15*	0.398	0.151	0.379	Segmental	Purifying	13.27
*CmaGSTU15*	vs.	*CmaGSTU16*	0.308	0.226	0.735	Tandem	Purifying	10.28
*CmaGSTZ1*	vs.	*CmaGSTZ2*	0.382	0.220	0.575	Tandem	Purifying	12.76
*CmaEF1G1*	vs.	*CmaEF1G2*	0.080	0.119	1.483	Segmental	Positive	2.68
*CmaEF1G1*	vs.	*CmaEF1G3*	0.536	0.293	0.547	Segmental	Purifying	17.89
*CmaEF1G2*	vs.	*CmaEF1G3*	0.566	0.279	0.493	Segmental	Purifying	18.87
*CmaGSTT1*	vs.	*CmaGSTT2*	0.201	0.130	0.646	Segmental	Purifying	6.72

Mya: Million year.

## Supplementary Materials

The following are available online at http://www.mdpi.com/2073-4425/9/2/84/s1, Figure S1: Sequence and structural analysis of pumpkin GST proteins with *Arabidopsis*, Rice and* Populus trichocarpa* GSTs; Figure S2: Genomic structures of *GST *genes of *Cucurbita maxima*.; Figure S3: Schematic representation of motif compositions in the *GST* sequences; Figure S4: Expression patterns of the *CmaGST *genes under various temperature treatments; Figure S5: Expression patterns of *CmaGST *genes under various temperature treatments; Table S1: List of GSTs from different species with their amino acid sequences used for phylogenetic analysis; Table S2: Primer sequence used for real time and reverse transcription polymerase chain reaction (RT-PCR) of *GST* genes of *C. maxima*; Table S3: Putative *cis*-elements, more than 4 bp, were identified in 32 *CmaGST* genes in *C. maxima* using Place Web Signal Scan.
