# Supplementary material for: Genome-Wide Identification, Characterization, and Expression Profiling of Glutathione S-Transferase (GST) Family in Pumpkin Reveals Likely Role in Cold-Stress Tolerance
Source: Genes (Basel). 2018 Feb 10;9(2):84. doi: 10.3390/genes9020084 (PMC5852580; doi:10.3390/genes9020084)
Supplement: Supplementary file 1 [file genes-09-00084-s001.zip › Supplementary materials/Supplementary Figures.pptx]

## Slide 1
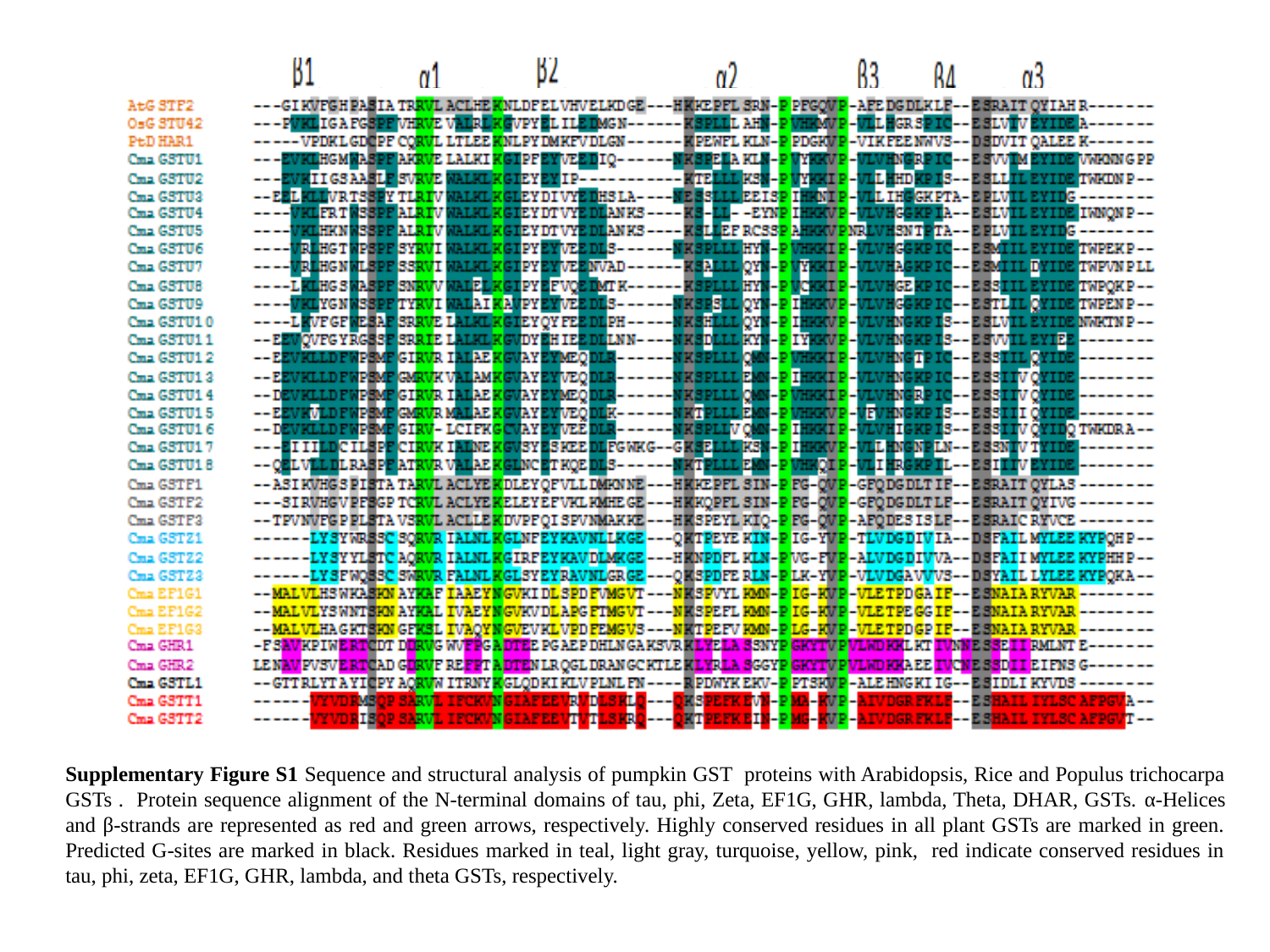

Supplementary Figure S1 Sequence and structural analysis of pumpkin GST proteins with Arabidopsis, Rice and Populus trichocarpa GSTs . Protein sequence alignment of the N-terminal domains of tau, phi, Zeta, EF1G, GHR, lambda, Theta, DHAR, GSTs. α-Helices and β-strands are represented as red and green arrows, respectively. Highly conserved residues in all plant GSTs are marked in green. Predicted G-sites are marked in black. Residues marked in teal, light gray, turquoise, yellow, pink, red indicate conserved residues in tau, phi, zeta, EF1G, GHR, lambda, and theta GSTs, respectively.

## Slide 2
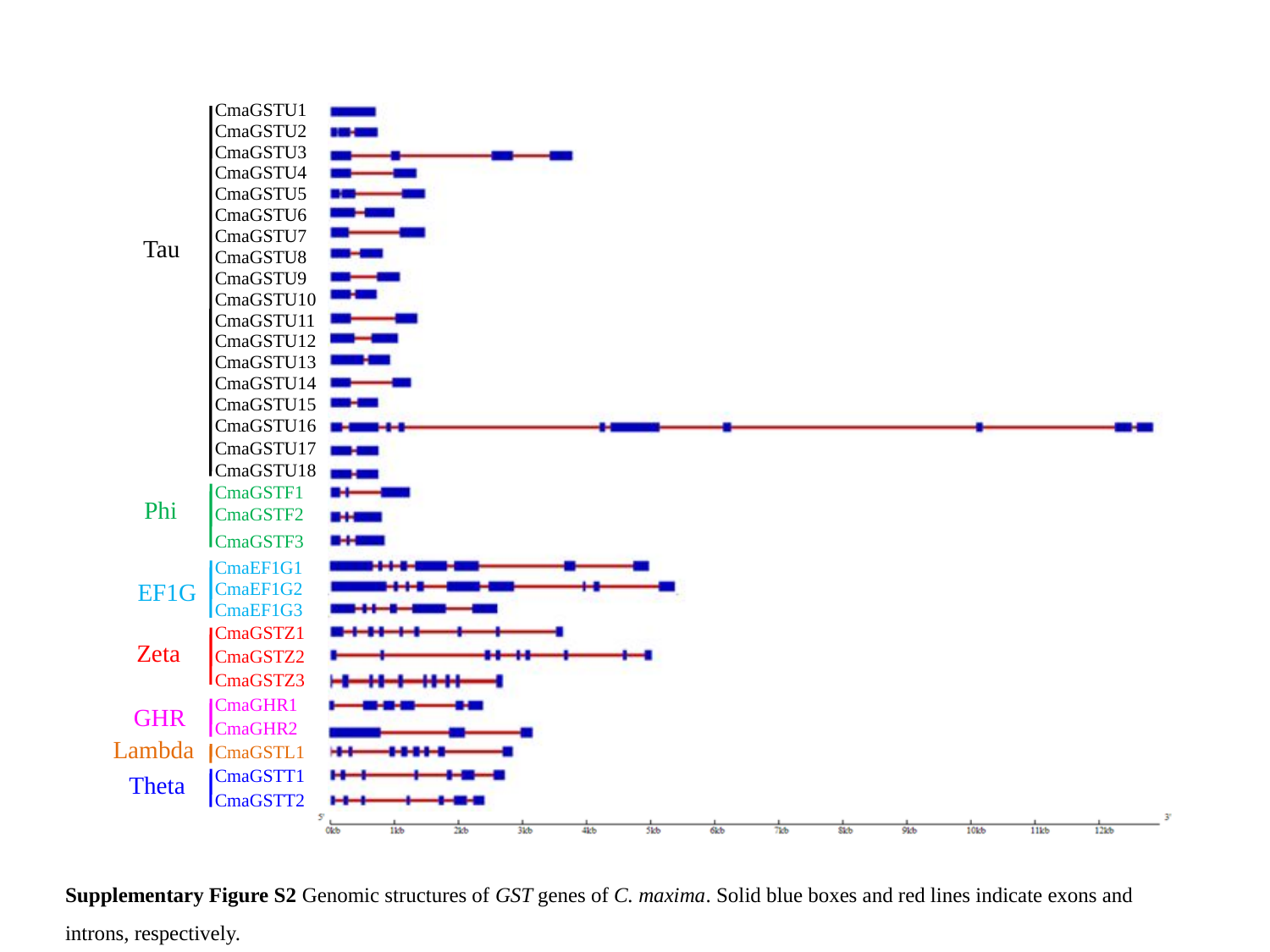

CmaGSTU1
CmaGSTU2
CmaGSTU3
CmaGSTU4
CmaGSTU5
CmaGSTU6
CmaGSTU7
CmaGSTU8
CmaGSTU9
CmaGSTU10
CmaGSTU11
CmaGSTU12
CmaGSTU13
CmaGSTU14
CmaGSTU15
CmaGSTU16
CmaGSTU17
CmaGSTU18
CmaGSTF1
CmaGSTF2
CmaGSTF3
CmaEF1G1
CmaEF1G2
CmaEF1G3
CmaGSTZ1
CmaGSTZ2
CmaGSTZ3
CmaGHR1
CmaGHR2
CmaGSTL1
CmaGSTT1
CmaGSTT2
Tau
Phi
EF1G
Zeta
GHR
Lambda
Theta
Supplementary Figure S2 Genomic structures of GST genes of C. maxima. Solid blue boxes and red lines indicate exons and introns, respectively.

## Slide 3
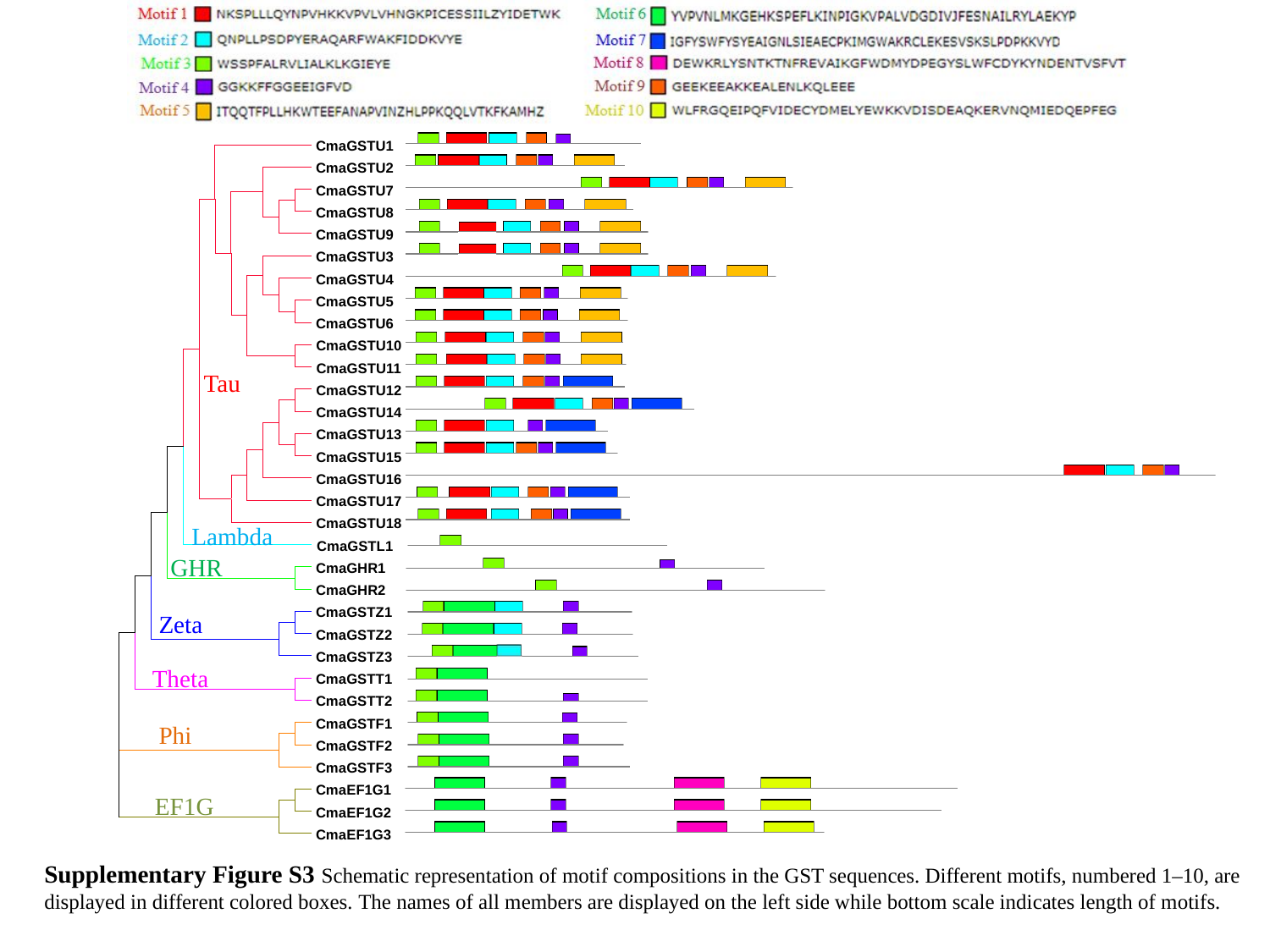

CmaGSTU1
 CmaGSTU2
 CmaGSTU7
 CmaGSTU8
 CmaGSTU9
 CmaGSTU3
 CmaGSTU4
 CmaGSTU5
 CmaGSTU6
 CmaGSTU10
 CmaGSTU11
 CmaGSTU12
 CmaGSTU14
 CmaGSTU13
 CmaGSTU15
 CmaGSTU16
 CmaGSTU17
 CmaGSTU18
 CmaGHR1
 CmaGHR2
 CmaGSTZ1
 CmaGSTZ2
 CmaGSTZ3
 CmaGSTT1
 CmaGSTT2
 CmaGSTF1
 CmaGSTF2
 CmaGSTF3
 CmaEF1G1
 CmaEF1G2
 CmaEF1G3
 CmaGSTL1
Tau
Lambda
GHR
Zeta
Theta
Phi
EF1G
Supplementary Figure S3 Schematic representation of motif compositions in the GST sequences. Different motifs, numbered 1–10, are displayed in different colored boxes. The names of all members are displayed on the left side while bottom scale indicates length of motifs.

## Slide 4
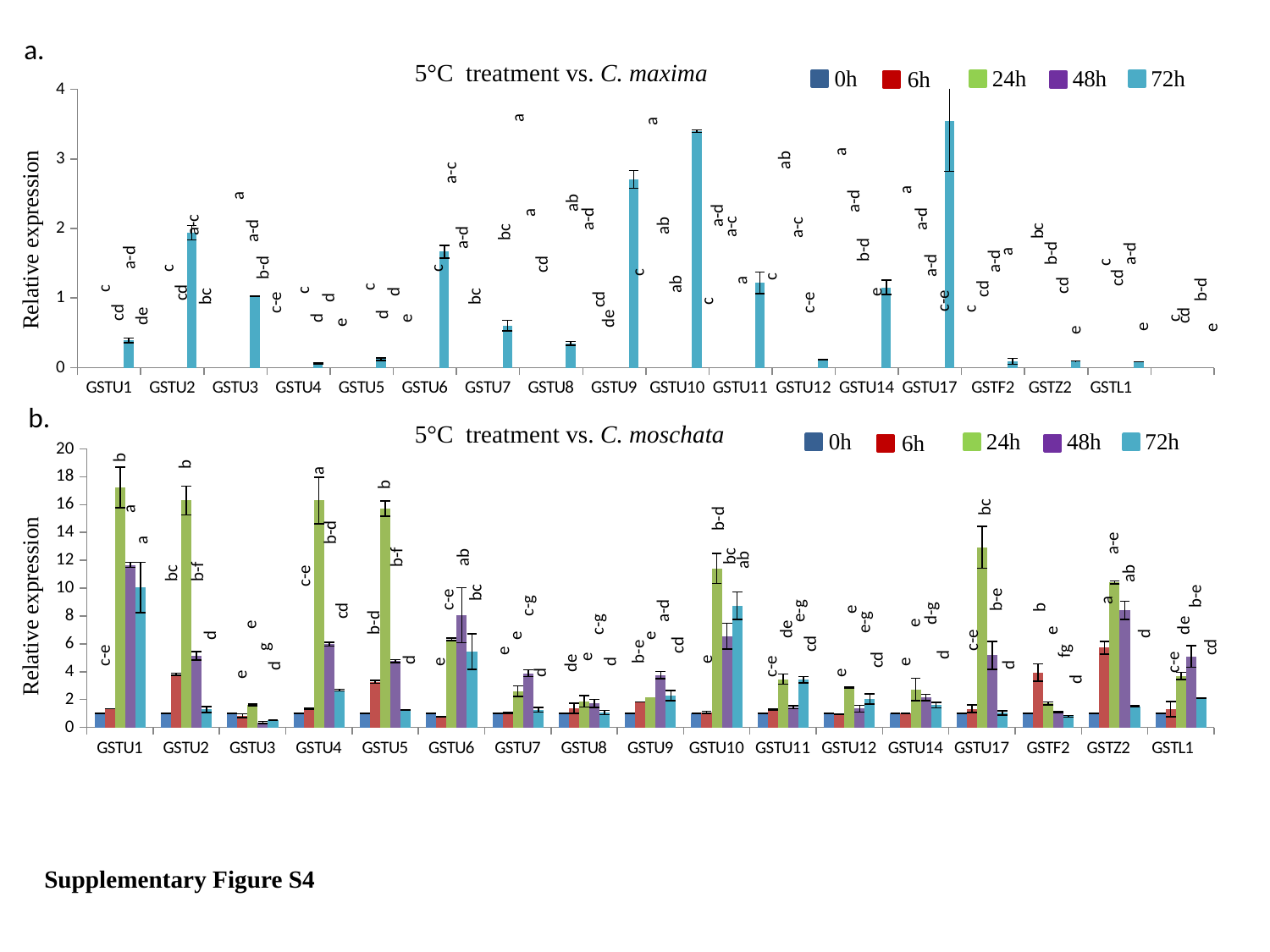

a.
5°C treatment vs. C. maxima
0h
24h
48h
72h
6h
### Chart
| Category | | | | | |
|---|---|---|---|---|---|
| GSTU1 | 1.0 | 1.1654331561897826 | 0.7100773685113728 | 3.732938896546673 | 0.3903405984468141 |
| GSTU2 | 1.0 | 0.5665644139932836 | 0.6398039026779696 | 6.041926627842925 | 1.9399885415935265 |
| GSTU3 | 1.0 | 8.350000000000007 | 5.1162598220850555 | 2.3560211012986 | 1.0293367762542098 |
| GSTU4 | 1.0 | 0.6245401645820625 | 0.44967313462338676 | 0.44682564552074605 | 0.05796424672011694 |
| GSTU5 | 1.0 | 0.6115551223811376 | 0.5216837156315146 | 0.7969022195288606 | 0.1244217373151986 |
| GSTU6 | 1.0 | 1.2112223889377818 | 8.480736053821445 | 3.938231332312941 | 1.6656071088974824 |
| GSTU7 | 1.0 | 3.762138747077402 | 12.946148555431 | 7.456803048933542 | 0.6053532566623782 |
| GSTU8 | 1.0 | 7.41108326501683 | 6.444824176658579 | 1.1910359296053266 | 0.350503935175244 |
| GSTU9 | 1.0 | 2.1466456117956367 | 12.9327940863509 | 6.113344467359476 | 2.70555434521368 |
| GSTU10 | 1.0 | 1.6270030270817601 | 6.72212069491604 | 5.929760187924423 | 3.3987604082609604 |
| GSTU11 | 1.0 | 2.517735261311241 | 9.771228475838482 | 5.661981782210585 | 1.2191276430821696 |
| GSTU12 | 1.0 | 10.9149073413106 | 6.74748164180766 | 2.2955541807805 | 0.1186855825288533 |
| GSTU14 | 1.0 | 8.007560354126705 | 5.39112501061933 | 3.133897297346061 | 1.1544282570802398 |
| GSTU17 | 1.0 | 1.0871525893168077 | 1.4773918641621928 | 3.2387217153556436 | 3.543635526090444 |
| GSTF2 | 1.0 | 3.7896555374036867 | 2.4104915732108267 | 1.3400385224909321 | 0.09354617295918514 |
| GSTZ2 | 1.0 | 2.669304293669666 | 1.9841489576890898 | 4.1510116019853776 | 0.09572294969526826 |
| GSTL1 | 1.0 | 0.5957609330106246 | 0.619672817663638 | 1.5210609643495403 | 0.0877778047336247 |a
a
a
ab
a-c
a
a
ab
a-d
a
Relative expression
a-d
a-d
a-d
ab
a-c
a-c
a-c
bc
bc
a-d
a-d
a
b-d
b-d
a-d
c
a-d
c
c
cd
a-d
c
a-d
b-d
c
a
cd
c
c
ab
c
cd
d
e
cd
cd
b-d
d
bc
bc
c
cd
c-e
c
c-e
c-e
d
cd
c
d
e
cd
de
e
de
e
e
e
b.
5°C treatment vs. C. moschata
### Chart
| Category | | | | | |
|---|---|---|---|---|---|
| GSTU1 | 1.0 | 1.3379275547861103 | 17.2230809994407 | 11.672793651254402 | 10.034029054535335 |
| GSTU2 | 1.0 | 3.8142044232756467 | 16.296201038925062 | 5.14079112014642 | 1.2878893641001958 |
| GSTU3 | 1.0 | 0.8293154549315916 | 1.6062875508286065 | 0.3509934478587506 | 0.5105183266115235 |
| GSTU4 | 1.0 | 1.3380561191102864 | 16.290392549352013 | 5.968297915378193 | 2.6761122382205658 |
| GSTU5 | 1.0 | 3.263567334894421 | 15.70938289498324 | 4.7643336621617856 | 1.2312627166483279 |
| GSTU6 | 1.0 | 0.7607131824875116 | 6.31809253254866 | 8.060949613064045 | 5.4478763300377855 |
| GSTU7 | 1.0 | 1.0177791281390776 | 2.60204849670642 | 3.8915675159262597 | 1.2680666004988 |
| GSTU8 | 1.0 | 1.37368187967043 | 1.8717640590869955 | 1.722828790383955 | 1.0774108269351141 |
| GSTU9 | 1.0 | 1.8150383106343198 | 2.1369223476095 | 3.7576951148540068 | 2.2796788707050397 |
| GSTU10 | 1.0 | 1.0695089822394137 | 11.416770141639498 | 6.553540285025528 | 8.751792845945005 |
| GSTU11 | 1.0 | 1.275050551898676 | 3.4655380429692 | 1.446149732857474 | 3.4145549345684127 |
| GSTU12 | 1.0 | 0.962646472728116 | 2.84480131282283 | 1.3442757578711375 | 2.03229150264607 |
| GSTU14 | 1.0 | 1.010506062853597 | 2.7211599790774 | 2.142920888398648 | 1.5999910095404355 |
| GSTU17 | 1.0 | 1.3414736964329184 | 12.9429991069357 | 5.1711555912365865 | 1.0401432325412183 |
| GSTF2 | 1.0 | 3.93961302996714 | 1.7129271294857593 | 1.1020110002991395 | 0.7970849683399982 |
| GSTZ2 | 1.0 | 5.7204935176361955 | 10.410984937983475 | 8.410647870019536 | 1.5149614754872298 |
| GSTL1 | 1.0 | 1.3153297372142898 | 3.69987010911056 | 5.084931521682231 | 2.084981607464458 |b
b
a
b
a
bc
b-d
a
b-d
a-e
bc
ab
b-f
ab
bc
b-f
ab
c-e
Relative expression
bc
a
b-e
c-e
b-e
b
e
c-g
cd
a-d
e-g
d-g
e
e
e-g
e
c-g
b-d
de
d
de
e
e
d
c-e
g
cd
cd
e
cd
fg
d
e
b-e
e
d
d
c-e
e
e
cd
d
d
de
c-e
c-e
d
e
e
d
0h
24h
48h
72h
6h
Supplementary Figure S4

## Slide 5
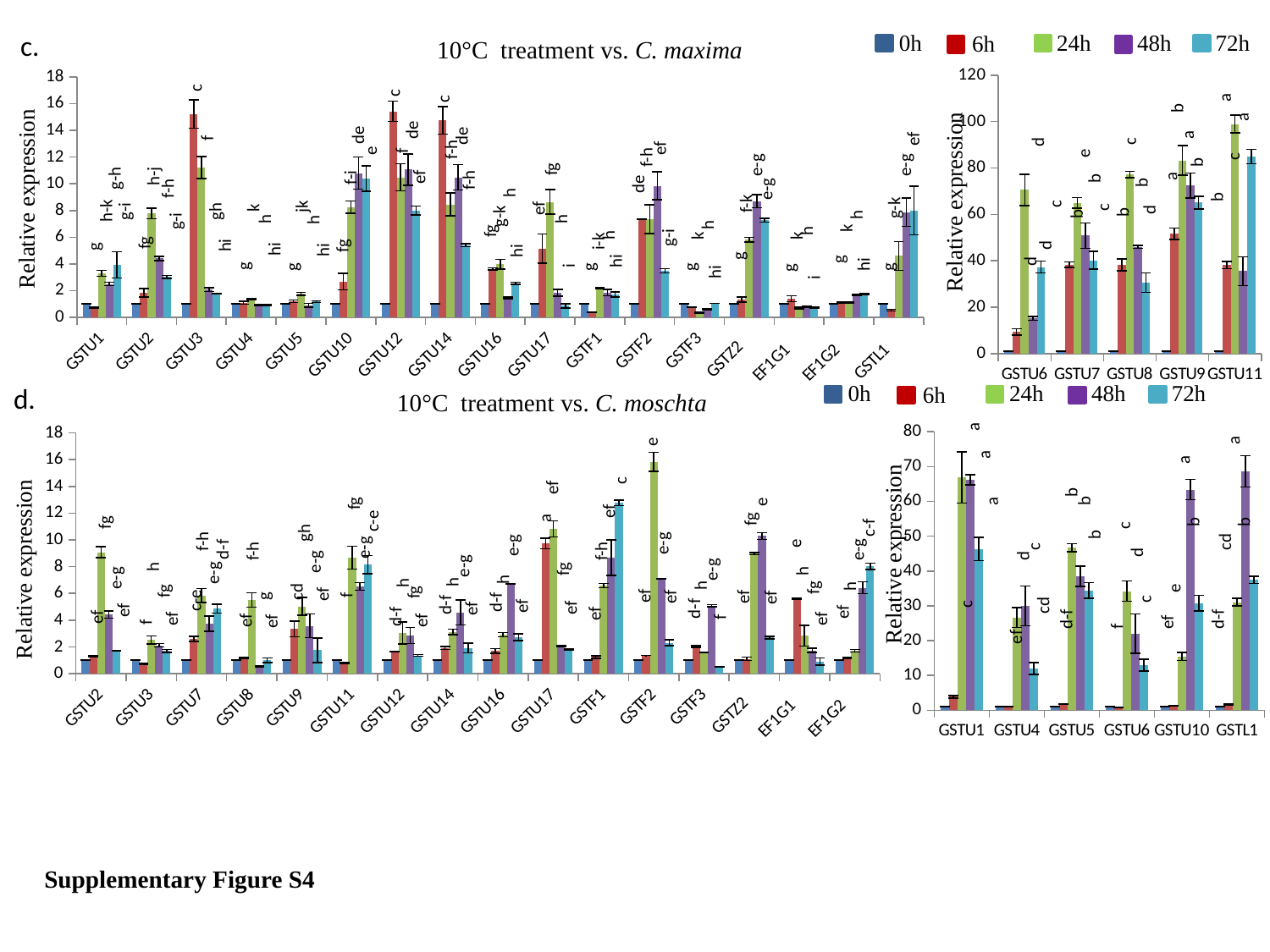

10°C treatment vs. C. maxima
### Chart
| Category | | | | | |
|---|---|---|---|---|---|
| GSTU6 | 1.0 | 9.317868691747638 | 70.52192741610295 | 15.136922347609548 | 37.28431653657473 |
| GSTU7 | 1.0 | 38.31931854780588 | 64.94818793201628 | 50.8351759563181 | 40.22442798469854 |
| GSTU8 | 1.0 | 38.124488886146295 | 77.18186775187628 | 46.001871510041894 | 30.5382254660622 |
| GSTU9 | 1.0 | 51.62507259021601 | 83.2229489194035 | 72.3447760452602 | 65.0647279748484 |
| GSTU11 | 1.0 | 38.05462768008696 | 98.91433496031516 | 35.50622310617121 | 84.8859315946603 |
### Chart
| Category | | | | | |
|---|---|---|---|---|---|
| GSTU1 | 1.0 | 0.7422617853145247 | 3.29436406907029 | 2.4966610978032167 | 3.9176811903477136 |
| GSTU2 | 1.0 | 1.8403753012497541 | 7.781239579298289 | 4.407620463506455 | 3.0314331330207946 |
| GSTU3 | 1.0 | 15.221482898665249 | 11.210073671684498 | 2.0849315216822575 | 1.7654059925813144 |
| GSTU4 | 1.0 | 1.094293701260742 | 1.34723357686569 | 0.9012504626108316 | 0.9201876506248791 |
| GSTU5 | 1.0 | 1.2141948843950479 | 1.753211442632065 | 0.9138314502294035 | 1.180992661429532 |
| GSTU10 | 1.0 | 2.675855109572216 | 8.2522194538948 | 10.794153738328799 | 10.392401564776632 |
| GSTU12 | 1.0 | 15.4200074017733 | 10.483147230866928 | 11.056106996174636 | 8.0 |
| GSTU14 | 1.0 | 14.7370469830041 | 8.456144324491053 | 10.483147230866985 | 5.3889343074627645 |
| GSTU16 | 1.0 | 3.6300766212686435 | 3.9723699817481317 | 1.4640856959456297 | 2.549121254638537 |
| GSTU17 | 1.0 | 5.1475007251520495 | 8.64646262152608 | 1.8403753012497541 | 0.8585654364377546 |
| GSTF1 | 1.0 | 0.37113089265726334 | 2.188587402521489 | 1.8403753012497501 | 1.7052697835359083 |
| GSTF2 | 1.0 | 7.351710219144445 | 7.351710219144445 | 9.84915530675936 | 3.4822022531845063 |
| GSTF3 | 1.0 | 0.7371346086455539 | 0.3368083942164243 | 0.6029039138453836 | 1.042465760841126 |
| GSTZ2 | 1.0 | 1.337927554786114 | 5.8158900692812345 | 8.693878900208489 | 7.260153242537285 |
| EF1G1 | 1.0 | 1.394743666350406 | 0.6973718331752032 | 0.7737824967711987 | 0.7269862586601566 |
| EF1G2 | 1.0 | 1.109569472067843 | 1.101905115876611 | 1.6934906247250563 | 1.7532114426320693 |
| GSTL1 | 1.0 | 0.5433674312630293 | 4.59479341998814 | 7.8765557765427685 | 8.0004678775104 |c
c
a
c
b
a
a
de
f
de
c
de
ef
d
f
ef
e
e
f-h
c
b
f-h
fg
e-g
e-g
Relative expression
a
b
ef
f-i
h-j
b
f-h
g-h
Relative expression
de
h
f-h
e-g
b
c
c
k
jk
f-k
d
ef
b
b
g-k
h
g-i
gh
h-k
h
h
h
g-k
h
g-i
k
h
fg
h
k
k
g-i
fg
i-k
d
g
hi
fg
hi
hi
hi
g
g
d
hi
i
g
g
g
g
g
hi
g
hi
i
c.
0h
24h
48h
72h
6h
0h
24h
48h
72h
6h
10°C treatment vs. C. moschta
a
### Chart
| Category | | | | | |
|---|---|---|---|---|---|
| GSTU1 | 1.0 | 3.8339130156452677 | 66.86635706026775 | 66.18310524301118 | 46.31677712652932 |
| GSTU4 | 1.0 | 0.9659595333687786 | 26.604717671422186 | 29.920885696137493 | 11.923217507242027 |
| GSTU5 | 1.0 | 1.7305700084252686 | 46.69566109831802 | 38.463669801228484 | 34.365343328242105 |
| GSTU6 | 1.0 | 0.7872997447646015 | 34.18621908387685 | 21.980092748068166 | 12.988856210988176 |
| GSTU10 | 1.0 | 1.2934706384023378 | 15.44013811460732 | 63.38404652265379 | 30.748504962764265 |
| GSTL1 | 1.0 | 1.6367866242259046 | 31.026402274602038 | 68.60833231628095 | 37.42805390534164 |
### Chart
| Category | | | | | |
|---|---|---|---|---|---|
| GSTU2 | 1.0 | 1.310676726174507 | 9.070910095822365 | 4.411432791116849 | 1.6876417276162403 |
| GSTU3 | 1.0 | 0.7152223763473329 | 2.5053026824447104 | 2.1144931603458037 | 1.6954844403886962 |
| GSTU7 | 1.0 | 2.587748740792598 | 5.838920619568339 | 3.7256768516187915 | 4.8624976224491006 |
| GSTU8 | 1.0 | 1.18144661969604 | 5.515390229708021 | 0.5558646055676625 | 0.9947978579983366 |
| GSTU9 | 1.0 | 3.3431311943736413 | 5.021570116246236 | 3.5663725210013792 | 1.7472900216399099 |
| GSTU11 | 1.0 | 0.8100304974571066 | 8.665101193319881 | 6.512911586873265 | 8.153969423318769 |
| GSTU12 | 1.0 | 1.6591087458660267 | 3.0240118192492513 | 2.8393918131668503 | 1.3504712850221556 |
| GSTU14 | 1.0 | 1.9124532891237394 | 3.100013449886325 | 4.570072128390383 | 1.8994581018817382 |
| GSTU16 | 1.0 | 1.6866837349776627 | 2.9201614422484012 | 6.7039373387283305 | 2.7197291112905893 |
| GSTU17 | 1.0 | 9.74734064834845 | 10.829904372639945 | 2.0711161314144038 | 1.8037025144853827 |
| GSTF1 | 1.0 | 1.2375634951634038 | 6.574262743593174 | 8.668015286781149 | 12.773464568684066 |
| GSTF2 | 1.0 | 1.35191895215668 | 15.843829592922082 | 7.086182709828897 | 2.3029178758812083 |
| GSTF3 | 1.0 | 2.02835741927841 | 1.6021782429770466 | 5.080878544169461 | 0.4780444241565739 |
| GSTZ2 | 1.0 | 1.102217127337827 | 8.978433647072697 | 10.29208209103467 | 2.6859352219981014 |
| EF1G1 | 1.0 | 5.606119448305047 | 2.8390819854312364 | 1.7317335087871764 | 0.8888640336093137 |
| EF1G2 | 1.0 | 1.164985415435572 | 1.7006050548484364 | 6.416104078935956 | 8.027822200226868 |a
e
a
a
c
ef
b
b
a
e
fg
ef
a
fg
b
b
fg
c
c-e
c-f
b
gh
Relative expression
e
Relative expression
c
cd
f-h
e-g
e-g
d
e-g
d-f
f-h
f-h
e-g
d
h
e-g
fg
h
e-g
e-g
e-g
h
h
h
e-g
h
h
e
fg
f
fg
fg
cd
g
ef
ef
c
ef
ef
ef
c
c-e
d-f
ef
cd
ef
d-f
ef
ef
d-f
ef
f
ef
ef
f
ef
ef
d-f
ef
ef
ef
d-f
f
ef
d-f
ef
d.
Supplementary Figure S4

## Slide 6
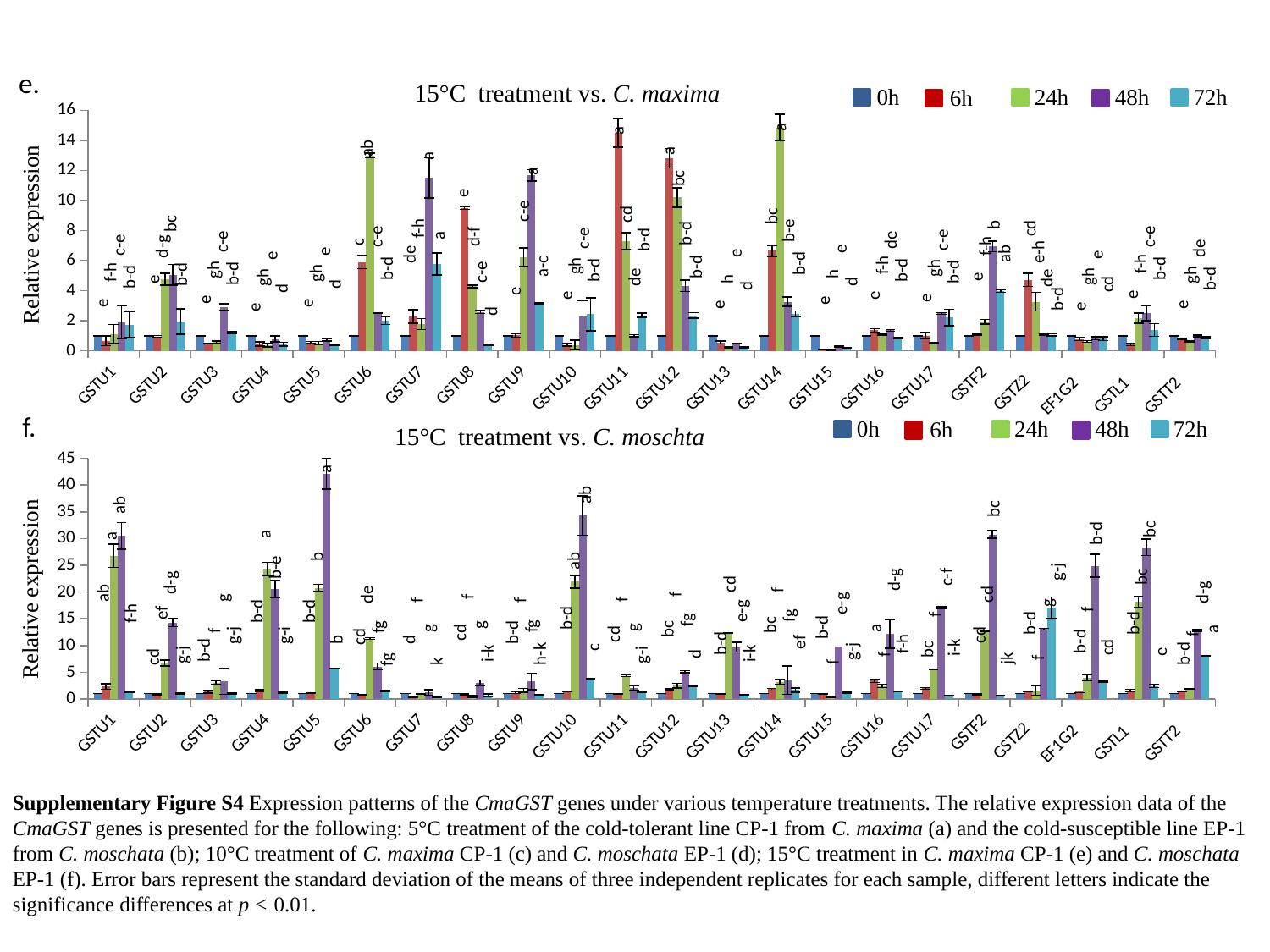

e.
15°C treatment vs. C. maxima
a
a
a
ab
a
a
bc
e
c-e
cd
bc
Relative expression
b
bc
cd
f-h
a
b-e
b-d
c
d-f
c-e
c-e
c-e
de
c-e
b-d
c-e
e
c-e
e
f-h
e
de
d-g
e
e
ab
de
e-h
f-h
gh
f-h
b-d
a-c
gh
gh
b-d
h
b-d
b-d
e
f-h
gh
b-d
b-d
c-e
e
h
gh
b-d
b-d
gh
b-d
gh
d
de
de
b-d
d
d
b-d
cd
d
e
e
e
e
e
e
e
e
e
e
e
b-d
e
e
d
0h
24h
48h
72h
6h
### Chart
| Category | | | | | |
|---|---|---|---|---|---|
| GSTU1 | 1.0 | 0.6651501762880208 | 1.1167555514208822 | 1.891215056440916 | 1.7435219037739358 |
| GSTU2 | 1.0 | 0.9635716003407976 | 4.771559426561352 | 5.051746367958684 | 1.9393733824031198 |
| GSTU3 | 1.0 | 0.4717348632260218 | 0.5705433651950246 | 2.9018010271002987 | 1.206850779213558 |
| GSTU4 | 1.0 | 0.46246537732592946 | 0.3716573983181934 | 0.7928495822663821 | 0.4301919811412399 |
| GSTU5 | 1.0 | 0.5522182455463566 | 0.5088210374411463 | 0.7114417799333629 | 0.3665843077066663 |
| GSTU6 | 1.0 | 5.91133168402084 | 12.991494189064802 | 2.5140267490436536 | 1.9942540178432244 |
| GSTU7 | 1.0 | 2.2807621530288587 | 1.7841417301874678 | 11.511348280375518 | 5.787950317000968 |
| GSTU8 | 1.0 | 9.491211066139167 | 4.2905790271656095 | 2.58285138239138 | 0.38842331282379 |
| GSTU9 | 1.0 | 1.034962568058078 | 6.2483605372304 | 11.6787222293096 | 3.1602411633033354 |
| GSTU10 | 1.0 | 0.39728785700448443 | 0.3984554421845629 | 2.257870121116601 | 2.431459300993672 |
| GSTU11 | 1.0 | 14.5071545949121 | 7.313183036738645 | 1.0151532556890968 | 2.364028298640714 |
| GSTU12 | 1.0 | 12.804435208099756 | 10.198197941922098 | 4.325315669574126 | 2.3492771720068326 |
| GSTU13 | 1.0 | 0.5545986480035537 | 0.2231134179710647 | 0.4781133222809332 | 0.2152992154756942 |
| GSTU14 | 1.0 | 6.6567712883575085 | 14.863828969739902 | 3.2673603355528082 | 2.4490410145427797 |
| GSTU15 | 1.0 | 0.09443231908854652 | 0.050949669481376865 | 0.2663337394241622 | 0.17541178767831309 |
| GSTU16 | 1.0 | 1.359244911420877 | 1.1065342687251938 | 1.357419139798178 | 0.8588954578595168 |
| GSTU17 | 1.0 | 1.0116495114531046 | 0.5310606720322335 | 2.4883968716623657 | 2.213423827662808 |
| GSTF2 | 1.0 | 1.106268589202358 | 1.9348435759365945 | 6.9450255860479055 | 3.98027624503247 |
| GSTZ2 | 1.0 | 4.73248253222087 | 3.26443040040174 | 1.0910382626250934 | 1.0544303329666629 |
| EF1G2 | 1.0 | 0.7911173910781005 | 0.6167703969409116 | 0.8438069735002987 | 0.8116075301860399 |
| GSTL1 | 1.0 | 0.42786578638993705 | 2.171744156615602 | 2.5057287105521215 | 1.388043265884085 |
| GSTT2 | 1.0 | 0.790724650261388 | 0.6096478858005386 | 0.9901381225162285 | 0.8834664814936979 |f.
15°C treatment vs. C. moschta
### Chart
| Category | | | | | |
|---|---|---|---|---|---|
| GSTU1 | 1.0 | 2.3081061116834403 | 26.7743124531465 | 30.5312120611958 | 1.3014669041679 |
| GSTU2 | 1.0 | 0.8456458872371092 | 6.730496162349732 | 14.2751754477899 | 1.0547847360339233 |
| GSTU3 | 1.0 | 1.3374787819788 | 3.0728526540019767 | 3.2948964993200978 | 1.0778269525268758 |
| GSTU4 | 1.0 | 1.5258134551365738 | 24.3257184945866 | 20.560885436891535 | 1.2126183579666456 |
| GSTU5 | 1.0 | 1.1260315928681544 | 20.796361836483687 | 42.1412568927275 | 5.779183297333971 |
| GSTU6 | 1.0 | 0.8066417592221292 | 11.275175447789916 | 6.069841685576 | 1.5442952443290479 |
| GSTU7 | 1.0 | 0.25105026122074797 | 0.9931879238218217 | 1.2021340918807 | 0.3440176396449024 |
| GSTU8 | 1.0 | 0.9058739152828786 | 0.4852952857638013 | 3.0061500434507997 | 0.6620591283900138 |
| GSTU9 | 1.0 | 1.158513194080194 | 1.5827952276224535 | 3.2422406601005 | 0.8094470777891845 |
| GSTU10 | 1.0 | 1.4442413898320998 | 21.948464850959667 | 34.2800173042587 | 3.8105519921757467 |
| GSTU11 | 1.0 | 0.9012504626108316 | 4.334007643390574 | 2.042243083388782 | 1.2060685373872246 |
| GSTU12 | 1.0 | 1.809030331791448 | 2.4732773096639997 | 5.098535009777379 | 2.41341679777365 |
| GSTU13 | 1.0 | 1.0000960921416906 | 12.294223607977841 | 9.700946863733149 | 0.7075314881750052 |
| GSTU14 | 1.0 | 1.9520752449992178 | 3.179940433052887 | 3.52585088278 | 1.675597776928 |
| GSTU15 | 1.0 | 0.9592641193252656 | 0.3499153788105044 | 9.781122221536542 | 1.1584669894764061 |
| GSTU16 | 1.0 | 3.3951209343610778 | 2.385331013439519 | 12.16250679406214 | 1.357125788561651 |
| GSTU17 | 1.0 | 1.9747876730553944 | 5.47031444385533 | 17.0631507756747 | 0.6736815177127289 |
| GSTF2 | 1.0 | 0.8713035373727667 | 12.712452758857706 | 30.77257234847349 | 0.6181582531414098 |
| GSTZ2 | 1.0 | 1.464648471504072 | 1.6417129487814501 | 13.0551586634554 | 17.0176324619206 |
| EF1G2 | 1.0 | 1.3656412959064608 | 4.01625030298028 | 24.928886488835087 | 3.2956303799043 |
| GSTL1 | 1.0 | 1.583712502502586 | 18.12084830826555 | 28.36516067828529 | 2.370160760088966 |
| GSTT2 | 1.0 | 1.4093969309737395 | 1.8728256486688117 | 12.7819872952744 | 8.106853112233319 |a
ab
ab
bc
bc
a
a
b-d
b
ab
b-e
g-j
Relative expression
bc
c-f
d-g
cd
f
d-g
f
f
ab
g
f
cd
f
f
de
d-g
g
f
e-g
f
ef
e-g
fg
f-h
b-d
b-d
fg
g
b-d
g
a
g
fg
f
fg
bc
a
b-d
b-d
f
bc
b-d
cd
cd
cd
g-j
g-i
b
d
b-d
cd
ef
c
f-h
b-d
b-d
cd
i-k
e
f
bc
d
g-j
f
b-d
i-k
i-k
g-j
g-i
f
jk
cd
k
h-k
b-d
fg
0h
24h
48h
72h
6h
Supplementary Figure S4 Expression patterns of the CmaGST genes under various temperature treatments. The relative expression data of the CmaGST genes is presented for the following: 5°C treatment of the cold-tolerant line CP-1 from C. maxima (a) and the cold-susceptible line EP-1 from C. moschata (b); 10°C treatment of C. maxima CP-1 (c) and C. moschata EP-1 (d); 15°C treatment in C. maxima CP-1 (e) and C. moschata EP-1 (f). Error bars represent the standard deviation of the means of three independent replicates for each sample, different letters indicate the significance differences at p ˂ 0.01.

## Slide 7
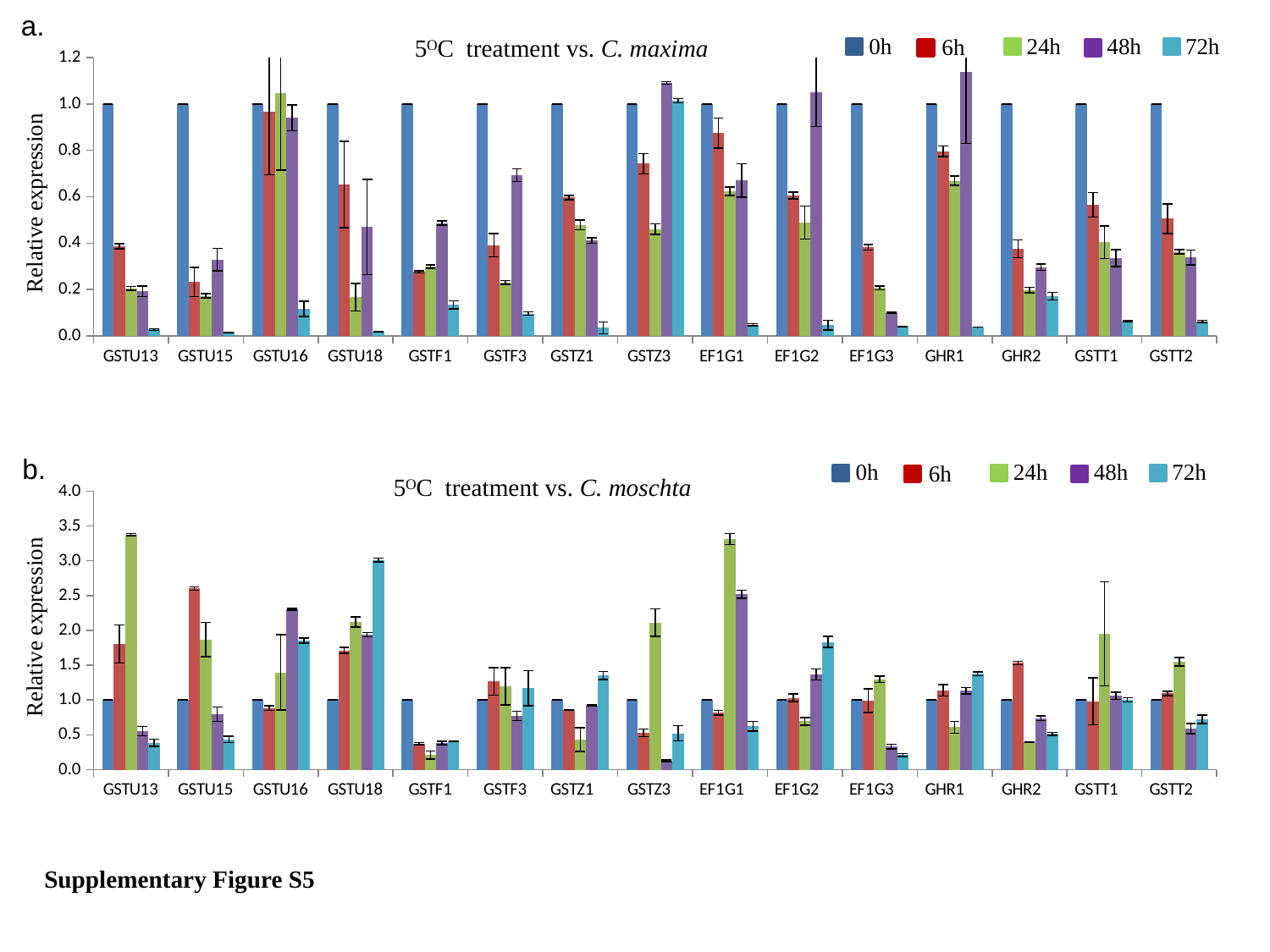

a.
5ᴼC treatment vs. C. maxima
0h
24h
48h
72h
6h
### Chart
| Category | | | | | |
|---|---|---|---|---|---|
| GSTU13 | 1.0 | 0.38697489877964664 | 0.20455411237607904 | 0.1927743375768654 | 0.02748874620728224 |
| GSTU15 | 1.0 | 0.2327741057216722 | 0.17266506638202136 | 0.3293706473437317 | 0.013534687867039905 |
| GSTU16 | 1.0 | 0.9652353403382412 | 1.0478391469595694 | 0.9403353800988786 | 0.11622180771167163 |
| GSTU18 | 1.0 | 0.6529084891586556 | 0.16688598992854567 | 0.47045383526647144 | 0.018585378678621114 |
| GSTF1 | 1.0 | 0.27644760491661985 | 0.2983787292033665 | 0.48637420595465786 | 0.1340931185402599 |
| GSTF3 | 1.0 | 0.39116499739162436 | 0.23091351366705298 | 0.6928209431156924 | 0.09620241593852413 |
| GSTZ1 | 1.0 | 0.5967001222800732 | 0.4782051915959321 | 0.41188454361896115 | 0.035313351868278615 |
| GSTZ3 | 1.0 | 0.742903797284866 | 0.46037016980262757 | 1.0905142818933988 | 1.0139838378819532 |
| EF1G1 | 1.0 | 0.8747535982196581 | 0.6243002262528334 | 0.6709092238653733 | 0.04832178655694857 |
| EF1G2 | 1.0 | 0.605087882086908 | 0.488958489455116 | 1.0513729153154414 | 0.0463405647692275 |
| EF1G3 | 1.0 | 0.38164730099449296 | 0.20740958137376056 | 0.10015538477840823 | 0.03956344579025739 |
| GHR1 | 1.0 | 0.7957084880083677 | 0.6691084151531935 | 1.1383959890362767 | 0.03807554940761121 |
| GHR2 | 1.0 | 0.3760033980993318 | 0.19768116256534649 | 0.2964173290861378 | 0.17171947459428327 |
| GSTT1 | 1.0 | 0.5657064670630346 | 0.4036564841259265 | 0.335454611932687 | 0.06252402418963593 |
| GSTT2 | 1.0 | 0.5055231916253705 | 0.3622899266110212 | 0.3387107236865969 | 0.06216373227615863 |Relative expression
b.
0h
24h
48h
72h
6h
5ᴼC treatment vs. C. moschta
### Chart
| Category | | | | | |
|---|---|---|---|---|---|
| GSTU13 | 1.0 | 1.8066417592221238 | 3.3752834552324837 | 0.5511085636689118 | 0.38724193755788217 |
| GSTU15 | 1.0 | 2.6027462344958527 | 1.8677574380697226 | 0.7962575805286867 | 0.4349186876897349 |
| GSTU16 | 1.0 | 0.883042295877455 | 1.3958224210615862 | 2.3053865389023285 | 1.853354199443413 |
| GSTU18 | 1.0 | 1.711446978549839 | 2.121999786367447 | 1.9386844169121935 | 3.010565815145336 |
| GSTF1 | 1.0 | 0.37260054710131385 | 0.20892433239510969 | 0.3818948329319334 | 0.4061359544449772 |
| GSTF3 | 1.0 | 1.2647516802134018 | 1.196032509083827 | 0.7699333281595157 | 1.170163036151365 |
| GSTZ1 | 1.0 | 0.8556001641028554 | 0.43151759758185554 | 0.9233878562576239 | 1.3525685351043848 |
| GSTZ3 | 1.0 | 0.5280760457206806 | 2.11129118918217 | 0.126939848866348 | 0.5215803464463745 |
| EF1G1 | 1.0 | 0.8182164495043172 | 3.313691767641566 | 2.51913781434937 | 0.6239829829656818 |
| EF1G2 | 1.0 | 1.0324329789866686 | 0.6936197749509977 | 1.3672217997277198 | 1.8349003278921678 |
| EF1G3 | 1.0 | 0.9900600176819847 | 1.2972212040333928 | 0.33296973694200815 | 0.21005188028391614 |
| GHR1 | 1.0 | 1.13835347056168 | 0.6080553140117126 | 1.133319351172533 | 1.3756739968990395 |
| GHR2 | 1.0 | 1.5316407800263918 | 0.39641349387159397 | 0.740053614244099 | 0.5125276339996035 |
| GSTT1 | 1.0 | 0.9797967740503286 | 1.9507859438535853 | 1.057652913483968 | 1.0 |
| GSTT2 | 1.0 | 1.094530300304256 | 1.5481598574604498 | 0.5906635335168325 | 0.7207158841020579 |Relative expression
Supplementary Figure S5

## Slide 8
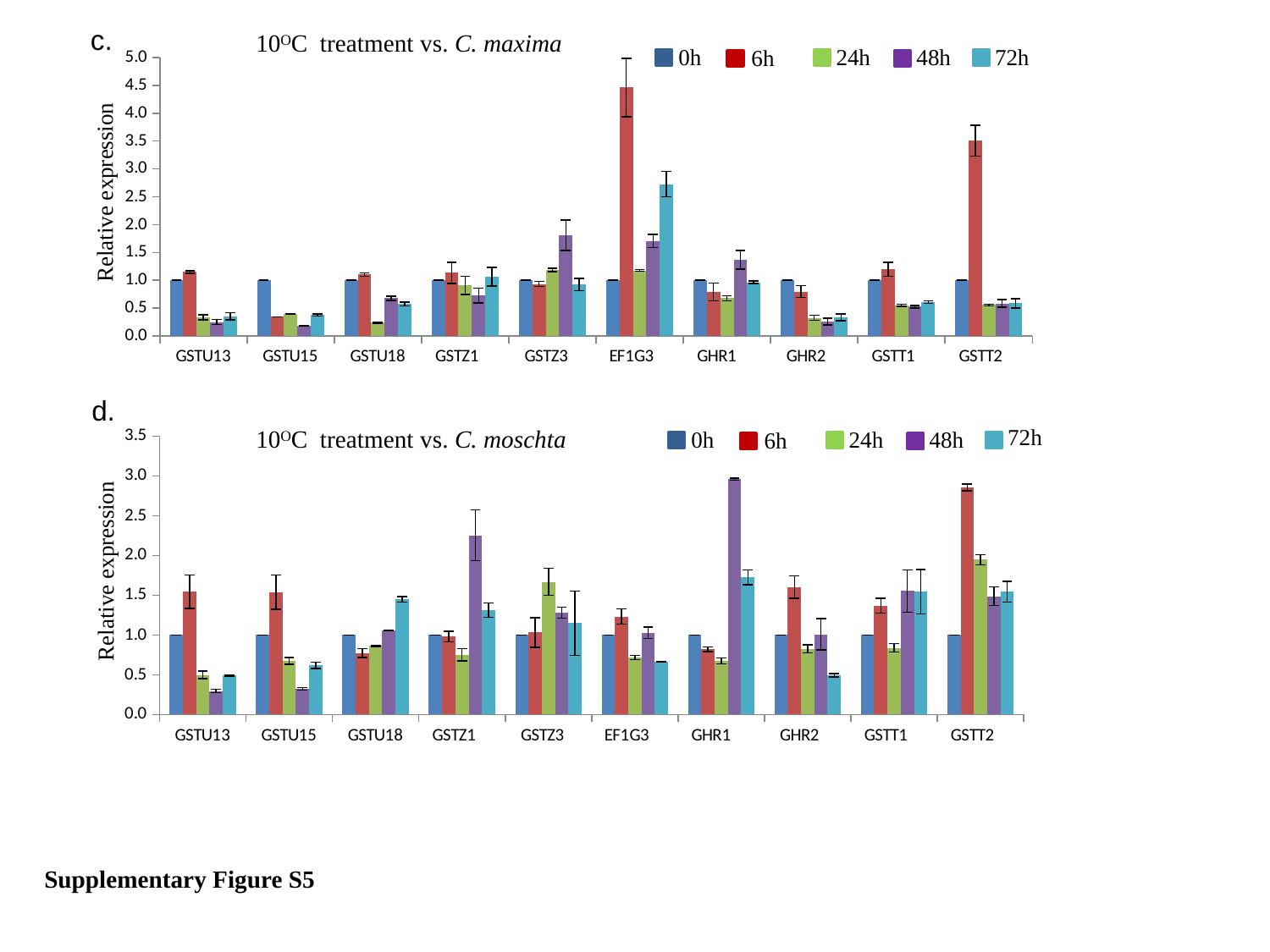

10ᴼC treatment vs. C. maxima
c.
### Chart
| Category | | | | | |
|---|---|---|---|---|---|
| GSTU13 | 1.0 | 1.1488087358821295 | 0.3347850725425844 | 0.24967763957545874 | 0.3527771726068449 |
| GSTU15 | 1.0 | 0.3426977593617683 | 0.39780646427972177 | 0.17989370364492074 | 0.37915673646739234 |
| GSTU18 | 1.0 | 1.1021433605966215 | 0.2399082120037705 | 0.6764466827949843 | 0.5729431014699179 |
| GSTZ1 | 1.0 | 1.1328782969851419 | 0.9055267187852856 | 0.7232043729093497 | 1.0635251540775639 |
| GSTZ3 | 1.0 | 0.9335933957678363 | 1.1852707070796074 | 1.8066417592221258 | 0.9233726561812641 |
| EF1G3 | 1.0 | 4.4623479776310555 | 1.176970350735588 | 1.7072774672842095 | 2.727355093085404 |
| GHR1 | 1.0 | 0.7897763131673339 | 0.676641598252648 | 1.3664270597653307 | 0.9627389721562627 |
| GHR2 | 1.0 | 0.7962575805286867 | 0.3235979727581906 | 0.2585036584893817 | 0.33044345045399726 |
| GSTT1 | 1.0 | 1.196497684815378 | 0.55107168029831 | 0.5250600905529053 | 0.6073308025827885 |
| GSTT2 | 1.0 | 3.507831593697141 | 0.5549046869554644 | 0.5843825141266423 | 0.5851109845310726 |0h
24h
48h
72h
6h
Relative expression
d.
10ᴼC treatment vs. C. moschta
### Chart
| Category | | | | | |
|---|---|---|---|---|---|
| GSTU13 | 1.0 | 1.544117145537733 | 0.49935079349754435 | 0.29667362919880363 | 0.4914368607123315 |
| GSTU15 | 1.0 | 1.5393000464179876 | 0.6766415982526494 | 0.3255308689234415 | 0.6183363730167555 |
| GSTU18 | 1.0 | 0.7721476221645251 | 0.8615513338728097 | 1.05701804056138 | 1.4491597064440838 |
| GSTZ1 | 1.0 | 0.9805734090964348 | 0.7520067961986658 | 2.253667179729014 | 1.3119361835206778 |
| GSTZ3 | 1.0 | 1.032992024539642 | 1.6688075676991687 | 1.2799151137207143 | 1.1492462396618461 |
| EF1G3 | 1.0 | 1.2330377209551937 | 0.7172532209103774 | 1.0293242670456706 | 0.6643588663897537 |
| GHR1 | 1.0 | 0.8209831464216684 | 0.6764466827949851 | 2.9587927879201494 | 1.7243446167424112 |
| GHR2 | 1.0 | 1.605258265473488 | 0.8272892715856222 | 1.0096244591465768 | 0.495069061258115 |
| GSTT1 | 1.0 | 1.3676485517288801 | 0.8388377994528302 | 1.5535706916733236 | 1.54416735172897 |
| GSTT2 | 1.0 | 2.8542868831130535 | 1.94717147902393 | 1.486806535765748 | 1.5448883172179317 |72h
0h
24h
48h
6h
Relative expression
Supplementary Figure S5

## Slide 9
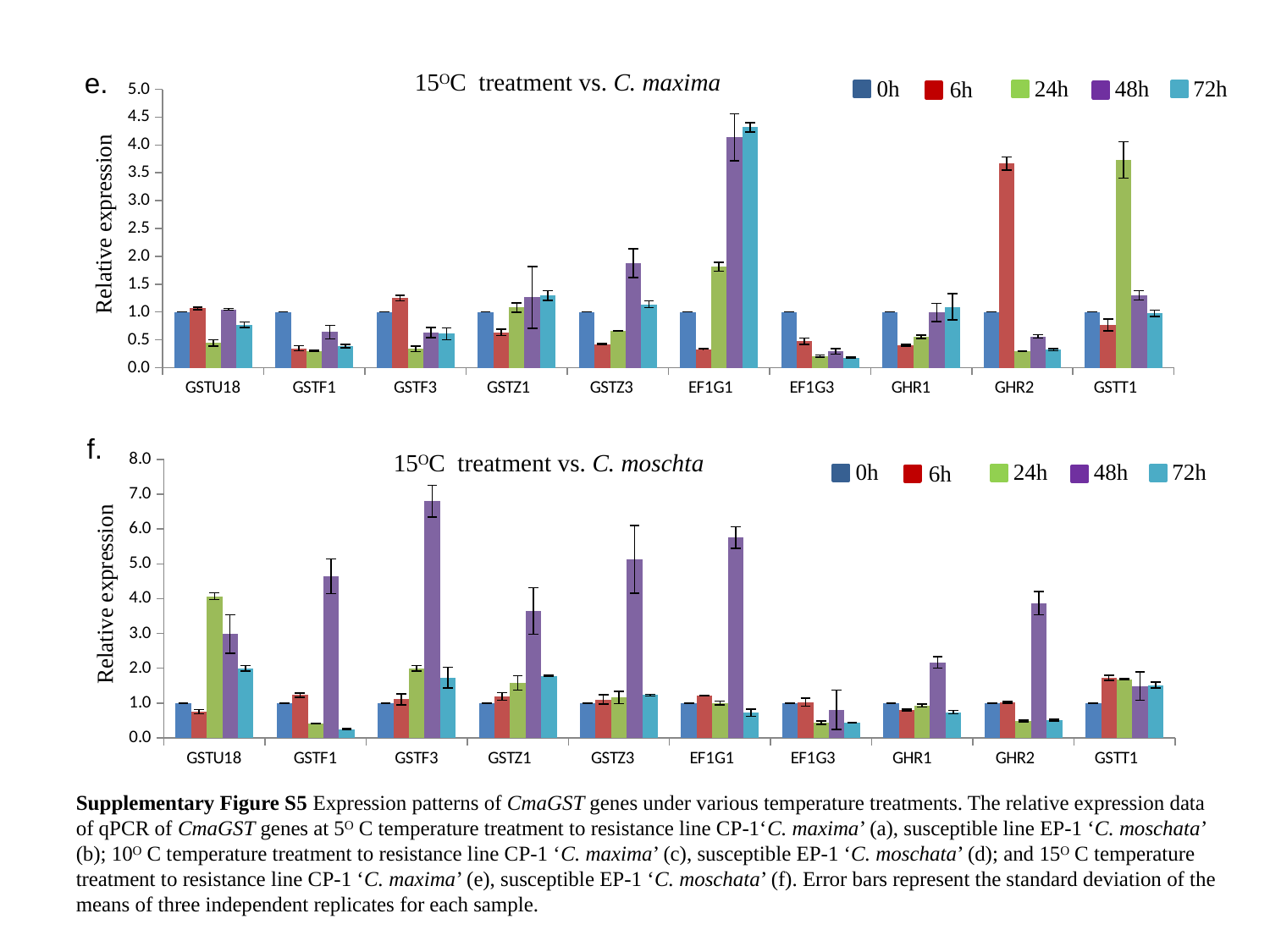

15ᴼC treatment vs. C. maxima
e.
### Chart
| Category | | | | | |
|---|---|---|---|---|---|
| GSTU18 | 1.0 | 1.064472460063751 | 0.444824176658603 | 1.0461414822013773 | 0.7718881839338376 |
| GSTF1 | 1.0 | 0.3490019753663556 | 0.3015171343468054 | 0.6389694082992317 | 0.3862220793044861 |
| GSTF3 | 1.0 | 1.2488103909789738 | 0.3409858781378924 | 0.6294983383516821 | 0.6076026098019386 |
| GSTZ1 | 1.0 | 0.6341101729671019 | 1.0808879207878108 | 1.2628922491706758 | 1.2938743703962934 |
| GSTZ3 | 1.0 | 0.4234133389665302 | 0.6574753286578139 | 1.8748591390185951 | 1.137643181345134 |
| EF1G1 | 1.0 | 0.3344899238702771 | 1.8096387128528382 | 4.137667182786213 | 4.3173277674281945 |
| EF1G3 | 1.0 | 0.47645387178437615 | 0.20706068663273094 | 0.29434660432678456 | 0.18010980145404942 |
| GHR1 | 1.0 | 0.40212103136873556 | 0.5532671343625972 | 0.9930875966919386 | 1.0918007182060978 |
| GHR2 | 1.0 | 3.66952042416073 | 0.29219606696254197 | 0.5609904557432206 | 0.32561685954366093 |
| GSTT1 | 1.0 | 0.7657835975621416 | 3.729724861058006 | 1.2981560136933281 | 0.9734962356634193 |0h
24h
48h
72h
6h
Relative expression
f.
15ᴼC treatment vs. C. moschta
### Chart
| Category | | | | | |
|---|---|---|---|---|---|
| GSTU18 | 1.0 | 0.7513216324707374 | 4.070529847682761 | 2.97728800770506 | 2.000768774068354 |
| GSTF1 | 1.0 | 1.227776644374394 | 0.4204886093956015 | 4.640208022875807 | 0.24956759872192447 |
| GSTF3 | 1.0 | 1.1078663865645515 | 2.000768774068354 | 6.804687127299487 | 1.7357845518925672 |
| GSTZ1 | 1.0 | 1.191521866021252 | 1.5815148553157798 | 3.6503521084657997 | 1.7838677520945407 |
| GSTZ3 | 1.0 | 1.102217127337829 | 1.1593454107021426 | 5.1270839787762785 | 1.2312627166483299 |
| EF1G1 | 1.0 | 1.2100013560429028 | 1.0042010448829828 | 5.759917433491721 | 0.723103705737941 |
| EF1G3 | 1.0 | 1.0278135593967221 | 0.43957751077045903 | 0.8092813608017996 | 0.43984831111567557 |
| GHR1 | 1.0 | 0.8040875575821632 | 0.9271445951266185 | 2.168881895592386 | 0.7380024673200976 |
| GHR2 | 1.0 | 1.0247106554489216 | 0.4865144116813993 | 3.8712659639464464 | 0.5125276339996042 |
| GSTT1 | 1.0 | 1.7239306005949386 | 1.687641727616246 | 1.4871768167397998 | 1.517027570523536 |0h
24h
48h
72h
6h
Relative expression
Supplementary Figure S5 Expression patterns of CmaGST genes under various temperature treatments. The relative expression data of qPCR of CmaGST genes at 5ᴼ C temperature treatment to resistance line CP-1‘C. maxima’ (a), susceptible line EP-1 ‘C. moschata’ (b); 10ᴼ C temperature treatment to resistance line CP-1 ‘C. maxima’ (c), susceptible EP-1 ‘C. moschata’ (d); and 15ᴼ C temperature treatment to resistance line CP-1 ‘C. maxima’ (e), susceptible EP-1 ‘C. moschata’ (f). Error bars represent the standard deviation of the means of three independent replicates for each sample.
