# Supplementary material for: Genome-Wide Identification, Characterization, and Expression Profiling of Glutathione S-Transferase (GST) Family in Pumpkin Reveals Likely Role in Cold-Stress Tolerance
Source: Genes (Basel). 2018 Feb 10;9(2):84. doi: 10.3390/genes9020084 (PMC5852580; doi:10.3390/genes9020084)
Supplement: Supplementary file 1 [file genes-09-00084-s001.zip › Supplementary materials/Supplementary Tables.doc]

**Supplementary Table S1** list of GSTs from different species with their amino acid sequences used for phylogenetic analysis

| Gene name | Protein sequences |
| --- | --- |
| CmaGSTU1 | MAEQNRVVLHGMWASPFAKRVELALKIKGIPFEYVEEDIQNKSPELAKLNPVYKKVPVLVHNGRPICESVVIMEYIDEVWKNNGPPLLPQDPYRRSQIRFWADFVQKQVFEGFFLSMTTEWEAQEKAIKEVKEKLKVLEEQGLKSLLAEAEGSPFVNGDELGFLDIVTLTVLGMYEIHEEFFGMKLVEEEKIPIMYSWLNRLKEHPVAKEAGAPKEKVLALLQFIRQKALRSTAA |
| CmaGSTU2 | MAEVKIIGSAASLFSVRVEWALKLKGIEYEYIPKTELLLKSNPVYKKIPVLLHHDKPISESLLILEYIDETWKDNPLIPEDPYNRAMARFWAKLLDEKGLLGAWEACQAEGEEKEKAVEAAIQNLALLDKEIQGKKFFGGDEIGYLDLAAGWMCHWLSVLDEVGEMKVFDKDRVPSLHEWVQNFIHIPVIKESLPPREDLVNYFKGSLSYVRSLAENKQ |
| CmaGSTU3 | MGEELKLLVRTSSPYTLRIVWALKLKGLEYDIVYEDHSLANESSLLLEEISPIHKNLPVLLIHGGKPTAEPLVILEYIDGVWKQNPLLSQDPYQRAVARFWVKFAEDKVLPSIINVFVRGNRREEAIGELEASRRRAKGKEILWKGKCWDCGHRGWSRGSELRREMGEELKLFRTWSTPFALRIVWALKLKGLEYDTVYEDLANKSPQLLEYNPVHKKVPVLVHGGKPIAESLVILEYIDEMWKQNPLLPQDPYQRAAARFWAKFGDDKVLESIRKVLMNQGKVREEGVEEALENMKHLEGELEGKRFFGGEAIGFVDIAVGWLANIVSVMEEVIGVELITEQRFPLLWKWIHEFAAAPIINETWPPRDKLVARYQAIDQASMSKQA |
| CmaGSTU4 | MEEEEGEVKLFRTWSSPFALRIVWALKLKGIEYDTVYEDLANKSSSLLEYNPIHKKVPVLVHGGKPIAESLVILEYIDEIWNQNPLLPQDPYQRALARFWAKFGDDKVWESMKKVFMKEGKLKEEGIEEAKENLKHLEEELKAGGKRFFGGEVIGFVDVALGWLANLPSVLEEVIGLSLITQQTFPLLSKWTHEFANAPIINQHWPPKQQLVTKFKAMHQIFMNKQA |
| CmaGSTU5 | MEEEEGEVKLFRTWSSPFALRIVWALKLKGIEYDTVYEDLANKSSSLLEFRCSSMAESRSPNRSSSSNTSMKYGTKIHCCLRILTREPLLYIDEIWNQNPLLPQDPYQRALARFWAKFGDDKVWESMKKVFMKEGKLKEEGIEEAKENLKHLEEELKAGGKRFFGGEVIGFVDVALGWLANLPSVLEEVIGLSLITQQTFPLLSKWTHEFANAPIINQHWPPKQQLVTKFKAMHQIFMNKQA |
| CmaGSTU6 | MAEVKLHGTWPSPFSYRVIWALKLKGIPYEYVEEDLSNKSPLLLHYNPVHKKIPVLVHGGKPICESMIILEYIDETWPEKPLLPSDPFERATARFWIKFIEDKGPAVFMIFRAKSDEEQENAKKQSLEMLRTVEEWGFRGGDGRERKFFGGDEIGMTDLAFGWIAHWIGVIEEVVNVKLLDANEFPKVYAWTERFKDAAVIRDNLPDRNLMAAAFRRRREQLLQST |
| CmaGSTU7 | MVPLPGQNSAGLPSTANPGGRNSTSLTPNDMNESVERIPVLRGMLLAEGEEDRAKVRKASLEVLQTLELPVFQEVVGIKVLEPDTFPRTFPRLHAWAENFRQAAVIRDSLPDHAQMSTRELPGQENSPIHRGIIKRKVGNDPKAITMADQVKLHGNWLSPFSSRVIWALKLKGIPYEYVEENVADKSALLLQYNPVYKKIPVLVHAGKPICESMIILDYIDETWPVNPLLPSDPFHRATARFWIKFIEDKSLVLWGVFMANGEDDREKARKATLEVLQTLEEQAFGDEKFFGGDEINMVDLAYGMLARWMPVIEEVVGVKLLDPDMFPRLHAWAENFRQAAVIRDNLPDHAQMVPLFKIRREQLLQIARK |
| CmaGSTU8 | MGGVKLHGSWASPFSNRVVWALELKGIPYEFVQEDMTKKSPLLLHYNPVCKKIPVLVHGEKPICESSIILEYIDETWPQKPLLPIDPLDRATARFWIKFIEDKEATMRRIFVARGEDKEEARRASLEVLRTVEERGLGGGGKFFGGDAINMVDLTYGWIAVWMKVLEEVLDMKLIEGPEFPRLQAWAESFRTAAVIRDHIPDHQKMVLHLRRRREELLLPPN |
| CmaGSTU9 | MGEVKLYGNWSSPFTYRVIWALAIKAVPYEYVEEDLSNKSPSLLQYNPIHKKVPVLVHGGKPICESTLILQYIDETWPENPLFPADPLDRAAARFWIRFAEDKVPVTWRMFCSDGEEHLKAKRESLEMLQIVEEGGIGEGKFFGGETIGIVDLVYGIFGDWLGVIEEVIGEKLVEADSFPRLHAWIRAFVEAPTIRENRPNRDRMVVTFKGLRERLLKSTSE |
| CmaGSTU10 | MAEQLQVFGFWESAFSRRVELALKLKGIEYQYFEEDLPHNKSHLLLQYNPIHKKVPVLVHNGKPISESLVILEYIDENWKTNPLLPQHPHDRAVARFWAKFIDDKVVVAVVKAAGSKGEEREKAVEEAGEALGALEKELGSRTLFGGERVGFVDVVGSVIVGWVPAIEECFGFEVLRVNKFPNLMKWSEEFAKHGVVKEILPPKHEIVAFMEANWKF |
| CmaGSTU11 | MAEEVQVFGYRSGSFSRRIELALKLKGVDYEHIEEDLLNNNKSDLLLKYNPIYKKVPVLVHNGKPISESVVILEYIEETWKHNPIFPEHPYDRAQARFWAKYIDDLVVPAMQKASLSKEEEREKAIEELHNKFEPLENELKNKKFFGGDEMGVVDIVGFVFAGWIPAIEEAVGFKLLNHKFPNLRKWIEEYANHTVAKQILPEKDALVIFIKNVSLKRKY |
| CmaGSTU12 | MAEEVKLLDFWPSMFGIRVRIALAEKGVAYEYMEQDLRNKSPLLLQMNPVHKKIPVLVHNGTPICESSIILQYIDEVWNDKAPLLPSDPYHRAQARFWVDFIDKKLCDAGRKVWGGRGEEAEAGMKELIGVLKQLEEVLGEKGFFGGERLGFVDIALIGFYSWFYSYEAFGKLSIEAECPKIMGWVKRCLEKESVSKSLPDSKKVYDCAVQMKKAHGLE |
| CmaGSTU13 | MVHSIGSCRKWTVFTASFPLLSFLFSLHYSILSHFLPNGSTLKLALNPSKLIALKRRSPFEFLLPHLATMAEEVKLLDFWPSMFGMRVKVALAMKGVAYEYVEQDLRNKSPLLLEMNPIHKKIPVLVHNGKPICESSIIVQYIDETWKNRAPLLPSDPYERAQARFWVDFIDKKVFESGRKIWASKGEEREEGKKEFMGSLKKLEEVLGEKAYFGGESVGFVDIVLIGYYTWFYSYESIGNFSVEAECSKIMGWAKRCLQNESLSNSLPDPFNICDFVLLMKKKYGLE |
| CmaGSTU14 | MADEVKLLDFWPSMFGIRVRIALAEKGVAYEYMEQDLRNKSPLLLQMNPVHKKIPVLVHNGRPICESSIIVQYIDEVWKDKAPLLPSDPYQRAQARFWVDFIDKKAGMKELIGVLKQLEEVLGEKSFFGGERLGFVDIALIGFHSWFYSYEAFGKLSIEAECPKIMGWAKRCLEKESVSKSLPDSKKVYDFVVQMKKALGLE |
| CmaGSTU15 | MAEEVKVLDFWPSMFGMRVRMALAEKGVAYEYVEQDLKNMTPLLLEMNPVHKKVPVFVHNGKPISESSIIIQYIDEVWKDRGPLLPSDPYERAEARFWVDFIDKKIYMSKGEEQETGKKEVIAILKQLEEALGERAYFGGECLGFVDIALIGFSSWFYSYETIGNFSIEAECPKIMVWVKRCLQKESVSSSLPDPIKVYHFLLKDRKALGLD |
| CmaGSTU16 | MALQLQMSWQPSLLSQKRRNGPPLGLRNLGNTCYLNSVLQCLTYTPPLANFCLRNQHSSLCDSASSNAERKRECPFCILERRIVRSLSLDLTLDSPLKIQNCLRIFAEHFRLGRQEDAHEFLRYVIDACHNTCLRLKKLRRTGGGVSGSSTVVKEIFGGALQSQVKCLSCGNDSNKVDEIMDISLDVLHSSSLKEALQKFFQLEVLDGSNKYKCDNCKKLVVARKQMSILQAPNTLVVQLKRFEGIFGGKIDKAIAYEESLQLSNFMCKGSQDPRPEYKLFGTIVHSGLSAESGHYYAYIKDASGRWYCCNDSFVTVSTLQEVLSEKVYILFFSRTNQRPVTANAALGSNGVKCHEGNGIEASKISKPSAVTKAAQTKSHVEQSSRKELSSLSKVEKPTFSQRGKSYMNGHSNSVQAPSTINGKIVLEKDQFIKENEKENVNSLHLENGVRHKSSLGNGNSKKSNEVADDVTERERKSVLMNGNGNCESVAMKEDKYDQCNGNPMNNKFTAGNGSGHDEVDNAVNCSSEIKMAKRKSDLCILFQQDALSRKRVGELKEDLRRETFSVLRTCGWSKEVFSCMRSRKRLCLREMGSTPSSNDLKKLLIADAKQKFISRIPEALKEDLIKKLQVFSQENLLNNLCIFKGCVAYEYVEEDLRNKSPLLVQMNPIHKKIPVLVHIGKPISESSIIVQYIDQTWKDRAPLLPSDPYERAQARFWVDFIDKKLYVETRKIWMNKGEQQEAGKKEMIAILKQLEEVLGEKAFFGGERLGFIDIGLIGFSTWFYSYETIGNFSIEAECPKIMGWVKRCL |
| CmaGSTU17 | MSKDEIIILDCILSPFCIRVKIALNEKGVSYESKEEDLFGWKGGKSELLLKSNPIHKKVPVLLHNGNPLNESSNIVTYIDETWPSSSPLLPSSPYERARARFWIDYIDKKVFETGGKIWRSNGDEQEAAKKEFIEILKQLEEALGDNDFFGGERFGIVDVIAIPFSCWFYAFEKVGGFKVEEHCPKLSNWEKRCLLKESVASVIPSPDMVYGFVSSMRKKLGLE |
| CmaGSTU18 | MSSSQELVLLDLRASPFATRVRVALAEKGLNCETKQEDLSNKTPLLLEMNPVHKQIPVLIHRGKPILESIIIVEYIDETWSSEAGYANLLPSHPYERSHARFWADYVDKKIYPNGGKLWNKKGLTEEEKEAAMKELLGSFKQLEEELGDKPYFGGQSFGFIDLSLIPFYSMFLAFKLLGNLDLEAECPKILEWAKRCCLRETVAKNMPTQQEVYDVVLDIIKRH |
| CmaGSTF1 | MASIKVHGSPISTATARVLACLYEKDLEYQFVLLDMKNNEHKKEPFLSINPFGQVPGFQDGDLTIFESRAITQYLASAYASKGNQLLSDDPKKAAIISTWIEVEGHHYDPPGVALSYEYLVKPLFGLGETDPAAVAENEAKLGKVLDIYENQLSKSKFLAGNEFSLADLHHIPNVHYLLATPSKKLFESRPRVNAWVAEITARPSWGKVLAMRNQKL |
| CmaGSTF2 | MATSIRVHGVPFSGPTCRVLACLYEKELEYEFVKLKMHEGEHKKQPFLSINPFGQVPGFQDGDLTLFESRAITQYIVGNYANNGTQLIFQDPKKAAIVLTWIEVESHHYDPPASKLVLELCLKPLLGWGETDAAVVEQSEAELAKVLDIYEDRLAQSKYLGGESFTLADLHHLPTIDYLFGTQMKKLFESRPHVNAWAVDIMARPAWAKVLALR |
| CmaGSTF3 | MATPVNVFGPPLSTAVSRVLACLLEKDVPFQISPVNMAKKEHKSPEYLKIQPFGQVPAFQDESISLFESRAICRYVCEKHAEKGNKGLYGLDQLQKASIDQWIEAEGQSFNPPSTILGFQLIFAPRMNVPQDEKAIKESEEKLSKVLDVYEQRLEQSRYLAGDEFSLADLSHLPNIHYLVNLTDRAELFTSRKNVERWWNEISGRESWKEVVEMQKQGSS |
| CmaGSTZ1 | MTENVASPPLKLYSYWRSSCSQRVRIALNLKGLNFEYKAVNLLKGEQFTPEYEKINPIGYVPTLVDGDIVIADSFAILMYLEEKYPQHPLLPSDLSKRAVNYQAANIVSSSIQPLQNLVVLKYIEEKSGPEEKLHWVQRVIEKGFAALEKLLKAHAGKFATGDEIFLADVFLAPQIHGAINRFSVDMSKFPLLSRLNDEYNAIAAFQDAEPSKQPDAPQSSS |
| CmaGSTZ2 | MAAGEGCPLKLYSYYLSTCAQRVRIALNLKGIRFEYKAVDLMKGEHLNPDFLKLNPVGFVPALVDGDIVVADSFAIIMYLEEKYPHHPLLPSDLYKRAINYQAANIVSSSIQPLQNLIVENYIEEKCGVEDKLSWVHMIIGKGFLALEKLLKDHAGKFATGDEIYLADFIFGTSTLPRAIETFNVDMSKFPLLSRLYEEYKEVPAFEDAAPEKQPDAPSQTSA |
| CmaGSTZ3 | MEFEAQAQSSDGSSTSSKLVLYSFWQSSCSWRVRFALNLKGLSYEYRAVNLGRGEQLSPDFERLNPLKYVPVLVDGAVVVSDSYAILLYLEEKYPQKALLPADLHLKSLNLQVASIVSSSIQPLIMLGLLKTIEEKFGPEEQLPWAQTNIENGFNALEKLLKNFSGRYALGEEVYMADVFLAPQVSVAFGRFNIDTSKFPALARIYESYKMLPEFQASSPERQPDALH |
| CmaEF1G1 | MALVLHSWKANKNAYKAFIAAEYNGVKIDLSPDFVMGVTNKSPVYLKMNPIGKVPVLETPDGAIFESNAIARYVARLKDSSLFGSSSIDYGHVEQWIDFSTMEIDAHISTILRPRFGYGVFHPAVEEAANAALKRSFAALNSYLASNTFLVGHSVTLADIILTCNLYLGFTYILTKSFTSEFPHVERYFWTLVNQPNFRKIIGEVKQTDAIPPVKTPEEAAAAAKPKPEPKKQEEKPKAAPAAEEEAPKPKAKNPLDLLPPSKMILDEWKRLYSNTKTNFREVAIKGFWDMYDPEGYSLWFCDYKYNDENTVSFVTLNKVSGFLQRMDLARKYAFGKMLVIGSEAPFKVKGLWLFRGQEIPQFVIDECYDMELYEWKKVDISDEAQKERVNQMIEDQEPFEGEALLDAKCFNFPFNFFHFSTASDRAFEFVAEVLDTDSQTIDMSRNLSRRSSDLVALSASLSLLFVSALSNDSSGKDSINSGKHSSSSSKTGSMVAIILLGFLVVVLLSFFLFKLWQKKRREEQYARLLKLFEEDDELEVELGLRD |
| CmaEF1G2 | MALVLYSWNTNKNAYKALIVAEYNGVKVDLAPGFTMGVTNKSPEFLKMNPIGKVPVLETPEGGIFESNAIARYVARLKDCGLFGSSPIEYGHVEQWIDFATLEIDVHIGALAGPRYGYRAYHAAVEEAANAALKRSFAALNSYLASNTYLVGHSVTLADIIVTCNLYWGISFLLTKSFTSEFPHVERYFWTMVNQPNFKKIIGEFKQTETVPPVKTPGEAAAAAKPKPEPKKEEKPKAAAPAEEEDAPKPKAKNPLDLLPPSKMILDEWKRLYSNTKTNFREVAIKGFWDMYDPEGYSLWFCDYKYNDENTVSFVTLNKVSGFLQRMDLARKYAFGKMLVIGSEAPFKVKGLWLFRGQEIPQFVMDECYDMELYEWKKVDISDEAQKERVNQMIEDQEPFEGEALLDAKCFKNFNQPSWFFTLQIDTEMSGNLSRRSFHFISFSALLSLLFVSALSDDSTPKDGIKSGNHPSSRSNTSSMIAIILLGLLVVVLSFFLFKLWQKKRREEQYARLLKLFEEDDELEVELGLRD |
| CmaEF1G3 | MALVLHAGKTNKNGFKSLIVAQYNGVEVKLVPDFEMGVSNKTPEFVKMNPLGKVPVLETPDGPIFESNAIARYVARLKADTTLCGSSVIEYGHIEQWIDFASLEIDSNMVTCYRPRMGRAPYLPPVEEAAIGALKRALGALNTYLASNTYLVGHSVTLADIIMTCNLFMGFTKLMTKSFTSDFPHVERYFWTLVNQPNIKRILGEVEQAESVLPIQSTKQTPKDEVKKEPKKEAAKVKPKAVVGEGDDETPKPKPKNPLDLLPPSKMILDEWKRLYSNTKTNFREIAIKGFWEMYDPEGYSLWFCDYKYNDENTVSFVTLNKVGGFLQRMDLARKYAFGKMLVIGSEAPFKVKGLWVFRGQEIPQFVVDECYDMELYEWRKVDISDEAQKERVNQMIEDKEPFEGEALLDAKCFK |
| CmaGHR1 | MLSALPSPQRTLRPLRLSDHCFTSLRFFCKMARSALDEMSTTGAFVRTASTFRNLISRDQSSQFPAESGRYHLYISYACPWASRCLAYLKIKGLEKAISFSSVKPIWERTKDTDDHRGWVFPGSDTEEPGAEPDHLNGAKSVRDLYELASSNYTGKYTVPVLWDKKLKTIVNNESSEIIRMLNTEFNDIAENPALDLYPPHLQAQIDETNGWIYDAINNGVYKCGFARMQQPYDQAVKELYEALDRCEDILSKQRYLCGNTLSEADVRLFVTIIRFDEVYAVHFKCNKKLLREYPNLFNYTKDIYQTKGVSSSVHMEHIKKHYYGSHPQINPFGIIPLGPNIDYTSPHDRHRFSS |
| CmaGHR2 | MPTGTAAVFSATNFLPPPLYPPPATSKTKPTRCHVIPRMSLNQSPSSGPKSSASSSSSSLLSSITNLLWGPSLPPGLLVATVRTAWETTWQLMMSQLAPSDSTGGYSRPLSQFRATKISANNLHLYVGLPCPWAHRTLIVRALKGLENAVPVSVAGPGADGSWEFRENPTADNENLRQGLDRANGCKTLKEVYRLRGGGYNGRSTVPMLWDAEKKEVICNESSDIIEIFNSGLNGLAENPDLDLSPPLLKGKIEEWNAIVYPNVNNGVYRCGFAQSQKAYDKAVNELFSTLDVLDEHLGRSRYLCGENLTLADVCLFTTLIRFDLVYNVLFKCTKKKLLEYANLHGYMRDIYQIPKVAATCNFTAIMDGYYKTLFPLNPGNIRPTKPASCYHEALSVPSKRESLPILDTTKQVSV |
| CmaGSTL1 | MVAFVEEVLPPSLDATAEQPPLFDGTTRLYTAYICPYAQRVWITRNYKGLQDKIKLVPLNLFNRPDWYKEKVYPTSKVPALEHNGKIIGESIDLIKYVDSNFEGPSLLPDDPAKKEYAEELISYSDTFNGAIISSFKGDTAKEAGAQFDYLENALQKFNGPFFLGEISQVDIVYIPFVERFQVFLLEAFKIDITEGRPKLAAWIEEFNKSDAYKQTKADPKLVVEVYTKRFLVTFALLHILHINAIFLKNLLTLLLC |
| CmaGSTT1 | MALKVYVDRMSQPSRAVLIFCKVNGIAFEEVRVDLSKLQQLSPEFKEVNPMAKVPAIVDGRFKLFESHAILIYLSCAFPGVADHWYPADLFRRAKINSVLDWHHSNLRIGAAPFVFYTVLAPLFGRPQNSQAAAEAEMLLSKSLSKIESFWLKGNGKFLVGSLKPSIADLSLVCEIMQLELLDEKERSRILGPYQKVREWVENTRNAANPHFDEVHKLLFKLKATLQKQRSEKLTSKM |
| CmaGSTT2 | MALKVYVDRISQPSRAVLIFCKVNGIAFEEVTVTLSKRQQLTPEFKEINPMGKVPAIVDGRFKLFESHAILIYLSCAFPGVTDHWYPADLSRRAKIHSVLDWHHSDLRRGSALLVFNTVLAPLFGRTPDPQVASEAELLLSKSLSKIESVWLKGNGKFLLGGFKPSIADLSLVCEITQLQLLDEKDRNRILGPHLKVREWLENTRNATNPQFDEVHRPLYMLKTALIKQGVEKFTSKM |
| AtGSTU5 | MAEKEEVKLLGIWASPFSRRVEMALKLKGIPYEYVEEILENKSPLLLALNPIHKKVPVLVHNGKTILESHVILEYIDETWPQNPILPQDPYERSKARFFAKLVDEQIMNVGFISMARADEKGREVLAEQVRELIMYLEKELVGKDYFGGKTVGFLDFVAGSLIPFCLERGWEGIGLEVITEEKFPEFKRWVRNLEKVEIVKDCVPPREEHVEHMNYMAERVRSS |
| ATGSTU9 | MDEEVENKVILHGSFASPYSKRIELALRLKSIPYQFVQEDLQNKSQTLLRYNPVHKKIPVLVHNGKPISESLFIIEYIDETWSNGPHILPEDPYRRSKVRFWANYIQLHLYDLVIKVVKSEGEEQKKALTEVKEKLSVIEKEGLKEIFSDTDGEPTVTNETMSLVDIVMCTLLSPYKAHEEVLGLKIIDPEIVPGVYGWINAINETSVVKDLSPPYEQILEILRAFRQMSLSPVLETYQS |
| ATGSTU19 | MANEVILLDFWPSMFGMRTRIALREKGVEFEYREEDLRNKSPLLLQMNPIHKKIPVLIHNGKPVNESIIQVQYIDEVWSHKNPILPSDPYLRAQARFWADFIDKKLYDAQRKVWATKGEEQEAGKKDFIEILKTLESELGDKPYFSGDDFGYVDIALIGFYTWFPAYEKFANFSIESEVPKLIAWVKKCLQRESVAKSLPDPEKVTEFVSELRKKFVPE |
| ATGSTF2 | MAGIKVFGHPASIATRRVLIALHEKNLDFELVHVELKDGEHKKEPFLSRNPFGQVPAFEDGDLKLFESRAITQYIAHRYENQGTNLLQTDSKNISQYAIMAIGMQVEDHQFDPVASKLAFEQIFKSIYGLTTDEAVVAEEEAKLAKVLDVYEARLKEFKYLAGETFTLTDLHHIPAIQYLLGTPTKKLFTERPRVNEWVAEITKRPASEKVQ |
| ATGSTF6 | MAGIKVFGHPASTATRRVLIALHEKNVDFEFVHVELKDGEHKKEPFILRNPFGKVPAFEDGDFKIFESRAITQYIAHEFSDKGNNLLSTGKDMAIIAMGIEIESHEFDPVGSKLVWEQVLKPLYGMTTDKTVVEEEEAKLAKVLDVYEHRLGESKYLASDHFTLVDLHTIPVIQYLLGTPTKKLFDERPHVSAWVADITSRPSAQKVL |
| ATGSTF9 | MVLKVYGPHFASPKRALVTLIEKGVAFETIPVDLMKGEHKQPAYLALQPFGTVPAVVDGDYKIFESRAVMRYVAEKYRSQGPDLLGKTVEDRGQVEQWLDVEATTYHPPLLNLTLHIMFASVMGFPSDEKLIKESEEKLAGVLDVYEAHLSKSKYLAGDFVSLADLAHLPFTDYLVGPIGKAYMIKDRKHVSAWWDDISSRPAWKETVAKYSFPA |
| ATGSTT1 | MMKLKVYADRMSQPSRAVIIFCKVNGIQFDEVLISLAKRQQLSPEFKDINPLGKVPAIVDGRLKLFESHAILIYLSSAFPSVADHWYPNDLSKRAKIHSVLDWHHTNLRRGAAGYVLNSVLGPALGLPLNPKAAAEAEQLLTKSLSTLETFWLKGNAKFLLGSNQPSIADLSLVCELMQLQVLDDKDRLRLLSTHKKVEQWIENTKKATMPHFDETHEILFKVKEGFQKRREMGTLSKPGLQSKI |
| ATGSTZ1 | MANSGEEKLKLYSYWRSSCAHRVRIALALKGLDYEYIPVNLLKGDQFDSVYRFDLQDFKKINPMGTVPALVDGDVVINDSFAIIMYLDEKYPEPPLLPRDLHKRAVNYQAMSIVLSGIQPHQNLAVIRYIEEKINVEEKTAWVNNAITKGFTALEKLLVNCAGKHATGDEIYLADLFLAPQIHGAINRFQINMEPYPTLAKCYESYNELPAFQNALPEKQPDAPSSTI |
| ATGSTL1 | MALSPPKIFVEDRQVPLDATSDPPALFDGTTRLYISYTCPFAQRVWITRNLKGLQDEIKLVPIDLPNRPAWLKEKVNPANKVPALEHNGKITGESLDLIKYVDSNFDGPSLYPEDSAKREFGEELLKYVDETFVKTVFGSFKGDPVKETASAFDHVENALKKFDDGPFFLGELSLVDIAYIPFIERFQVFLDEVFKYEIIIGRPNLAAWIEQMNKMVAYTQTKTDSEYVVNYFKRFM |
| ATGSTL2 | MSVGLKVSAFLHPTLALSSRDVSLSSSSSSLYLDRKILRPGSGRRWCKSRRTEPILAVVESSRVPELDSSSEPVQVFDGSTRLYISYTCPFAQRAWIARNYKGLQNKIELVPIDLKNRPAWYKEKVYSANKVPALEHNNRVLGESLDLIKYIDTNFEGPSLTPDGLEKQVVADELLSYTDSFSKAVRSTLNGTDTNAADVAFDYIEQALSKFNEGPFFLGQFSLVDVAYAPFIERFRLILSDVMNVDITSGRPNLALWIQEMNKIEAYTETRQDPQELVERYKRRVQAEARL |
| ATDHAR1 | MALEICVKAAVGAPDHLGDCPFSQRALLTLEEKSLTYKIHLINLSDKPQWFLDISPQGKVPVLKIDDKWVTDSDVIVGILEEKYPDPPLKTPAEFASVGSNIFGTFGTFLKSKDSNDGSEHALLVELEALENHLKSHDGPFIAGERVSAVDLSLAPKLYHLQVALGHFKSWSVPESFPHVHNYMKTLFSLDSFEKTKTEEKYVISGWAPKVNP |
| ATDHAR2 | MALDICVKVAVGAPDVLGDCPFSQRVLLTLEEKKLPYKTHLINVSDKPQWFLDISPEGKVPVVKLDGKWVADSDVIVGLLEEKYPEPSLKTPPEFASVGSKIFGAFVTFLKSKDANDGSEKALVDELEALENHLKTHSGPFVAGEKITAVDLSLAPKLYHLEVALGHYKNWSVPESLTSVRNYAKALFSRESFENTKAKKEIVVAGWESKVNA |
| AtTCHQD1 | MQLYHHPYSIDSQRVRLALEEKGIDYTSYHVNPITGKHMDPSFFRMNPNAKLPVFRNGSHIILDTIEIIEYLERIAEVSSGIEDATFNREVVEWMRKIREWESKLFTLAHIPDNRRLYVSKFLRMVVIARMAESPDLASAYHRKLREAYDTEDKLKDPGALRRSKDHLLRLLDEVETKLEGTTYLAGNEFSMADVMLIPVLARLSLLDLEEEYISSRKNLAEYWALVRRRPSYKKVIGRYFNGWRKYATLVKTWMFVRVRSLLRKY |
| AtGHR1 | MSYSTIISNTSFLSLASKFTTRGSRLQCTVSMARSAVDETSDSGAFQRTASTFRNFVSKDSNSQFPAESGRYHLYISYACPWASRCLSYLKIKGLDDAISFSSVKPIWGRTKETDEHMGWVFPGSDTEVPGADPDHLNGAKSVRELYEIASPNYTGKYTVPVLWDKKLKTVVNNESAEIIRMFNTEFNHIAGNPDLDLYPSHLQAKIDETNEWIYNGINNGVYRCGFAKKQGPYEEAVEQVYEALDRCEEILGKHRYICGNTLTETDIRLFVTLIRFDEVSSYFQSKKKKYTICERISNVETLIQVYAVHFKCNKKLIREYPNLFNYTKDIFQIPGMSSTVNMNHIKQHYYGSHPSINPFGIIPHGPNIDYTSPHDRHRFSK |
| AtGHR2 | MARSGVDETSESGAFVRTASTFRNFVSQDPDSQFPAESGRYHLYISYACPWACRCLSYLKIKGLDEAITFSSVHAIWGRTKETDDHRGWVFPDSDTELPGAEPDYLNGAKSVRELYEIASPNYEGKYTVPVLWDKKLKTVVNNESSEIIRMFNTEFNGIAKTPSLDLYPSHLRDVINETNGWVFNGINNGVYKCGFARKQEPYNEAVNQLYEAVDRCEEVLGKQRYICGNTFTEADIRLFVTLIRFDEVYAVHFKCNKRLLREYPNIFNYIKDIYQIHGMSSTVNMEHIKQHYYGSHPTINPFGIIPHGPNIDYSSPHDRDRFSS |
| AtGHR3 | MATPMENENPNFARTATSFRNFVSKDPDSQFPAESGRYHLYISYACPWASRCLAILKLKGLDKAISFSSVQPLWRNTKENDEHMGWVFPDSDTEVLGAERDHINGAKSVRELYDIASSNYTGKYTVPVLWDKKLKTIVNNESSEILRMFNTEFNHVAENPSLDLYPPNLRAIIDETNEWIHDGINNGVYKCGFATNQETYDVEVKRLYEALDRCEDILRKQRFLCGNTLTESDIRLFVTVIRFDEAYAVIFKCDKRLVREYYHLFNYTKDIYQIAGMSSTVKMDHIKQNYYGSFPSINPLEIIAHGPNIDYSLPHDRHRFSLESDYTRLELFESASFVCELKLIEIFDSL |
| AtGHR4 | MANCFAPQLTFPSFSPRHFSPRMSHQSPKPSTSTTTSIFTSATKLLWGPSLPPGLLISTARTAWTTVWQLMMTQLAPSDSSGSYTRPTSKFRLDPTQFTSAASSELHLYVGLPCPWAHRTLIVRALKGLNDAVPVSIASPGQDGSWEFKNNNIPIKDKDKLIPSLDKANRCRNLKEVYKSRSGGYDGRCTVPMLWDLRKKDVVCNESYDIIEFFNSGLNKLARNDNLDLSPPELKEMIQGWNQIVYPKVNNGVYRCGFAQSQEAYDGAVNELFSTLDEIEDHLGSNRYLCGERLTLADVCLFTTLIRFDSVYNILFKCTKKKLVEYPNLYGYLREIYQIPGVAATCDISAIMDGYYKTLFPLNASGIQPAISSSGDQDSLWRPHNRDLVGKAIEAQLSV |
| AtmPGES2 | MRRVTGLAARTISSSVAINSRLTQSMAITTISSSEPISRRFGGLPEIKTPSFAGGVAGVVFFSAAAVSSLGQEVHAKEMAQKFNPKEVVLYQYEACPFCNKVKAFLDYNKIPYKVVEVNPISKKEIKWSDYKKVPILTVDGEQMVDSSVIIDSLFQKMHPEISKSEDDEETKWRKWVDNHLVHLLSPNIYRNTSEALESFEYITTHGNFSFTERLVAKYAGATAMYFVSKKLKKKYNITDERAALYDAAETWVDALKERPYLGGSKPNLGDLAVFGVLRPIRYLRSGKDMVDNTRIGEWYSRMENTVGEPSRIKE |
| AtEFIG1 | MALVLHTYKGNKSAEKALIAAEYVGVQIDVPSDFQMGVTNKTPAFLKMNPIGKVPVLETPEGSVFESNAIARYVSRLNGDNSLNGSSLIEYAQIEQWIDFSSLEIYASILRWFGPRMGFMPYSAPAEEGAISTLKRALDALNTHLTSNTYLVGHSITLADIITVCNLNLGFATVMTKKFTSEFPHVERYFWTVVNQPNFTKVLGDVKQTEAVPPIASKKAAQPAKPKEEPKKKEAPVAEAPKLAEEEEAPKPKAKNPLDLLPPSPMVLDDWKRLYSNTKSNFREVAIKGFWDMYDPEGYSLWFCDYKYNDENMVSFVTLNKVGGFLQRMDLARKYSFGKMLICGSEGPFKVKGLWLFRGPEIPKFIMDEVYDMELYEWTKVDISDEAQKERVSQMIEDAEPFEGEALLDAKCFK |
| AtGST2N-1 | MAGILVNNILPQPPILRSLSSSSRRSSIRTLVMVKASSSEPSESVSVSTKTSDDTGAVVVFTAPPGFKPPEPKRFAVKSGKLFDVLGAAIGLFFRFGTGVFVSGYSASFVSKEEIPADQYALRLGGITVKETAKVGPRPEKPIEIYEFEGCPFCRKVREMVAVLDLDILYYPCPRGSPNFRPKVKQMGGKQQFPYMVDPNTGVSMYESDGIIKYLSEKYGDGTVPLSLSLGALTAITAGFAMIGRMGKGNLYTPAKLPPKPLEFWAYEGSPFCKLVREVLVELELPHIQRSCARGSPKRQVLLEKAGHFQVPYLEDPNTGVAMFESAEIVEYLKQTYAA |
| OsGSTU3 | MAGGGDELKLLGMWASPFALRAKLALSFKGLSYEYVEEDLKNKSELLLTSNPVHKKVPVLIHNGKPVCESQVIVQYIDEAFPDAGVPLLPSDPYDRAVARFWAAYIDDKLLKSWLQASMCKTEQEKAAAMKETFAAVANLEAAFKECSKGKPFFGGDAVGYVDVTLGAVIGFVRVGEAVHGMRLFDASRSPLLDAWLDRFAALDAAKAVLPDTGRLAEYAKMKQAEWAAAATN |
| OsGSTU4 | MAGSGDELMLLGKWPSPFVTRVELALGLKGLSYEYVKQDLVNKSELLLASNPVHKKIPVLIHNGKPVCESSIIVQYIDEAFPDAGAGAALLPADPYERAVARFWVAYVDDKFVPAWVATFRGKTEEEKAEGMKQLLAAVETLEGALKDCSKGKPFFGGDTVGIVDVALGGLISWVKATEVLAGSKIFDEEKAPLLAAWAQRFGELDVAEKVLPDVDGVVEFAKMRLAEAAAAAAAASKN |
| OsGSTU13 | MAGKDDDVKVLGVVVSPFAIRVRIALNIKGVSYEYVEEDIFNKSELLLTSNPVHKKVPVLIHGGKPISESLVIVQYVDEVWAAAPSVLPADPYDRAVARFWAAYVDNNMFPGMAGVLFAATEEERAAKAEETLAALAQLEKAFAECAGGKAFFGGDSIGYVDLALGSNLHWFEALRRLFGVALLDAGKTPLLAAWAKRFVEAEAAKGVVPDAGVAVELGKKLQARAAAASTAA |
| OsGSTF2 | MAPMKLYGSTLSWNVTRCVAVLEEAGAEYEIVPLDFSKGEHKAPDHLARNPFGQVPALQDGDLFLWESRAICKYVCRKNKPELLKDGDLKESAMVDVWLEVESNQYTPALNPILFQCLIRPMMFGAPPDEKVVEENLEKLKKVLEVYEARLTKCKYLAGDYISVADLSHVAGTVCLGATPHASVLDAYPHVKAWWTDLMARPSSQKVASLMKPPA* |
| OsGSTF3 | MAAPVTVYGPMISPAVARVAACLLEKDVPFQVEPVDMSKGEHKSPSFLKLQPFGQVPAFKDSLTTVFESRAICRYICDQYADSGNKTLMGRKEDGAVGRAAIEKWIEAEGQSFNPPSLAMAFQLAFAPFMGRATDMAVVEQNEAKLVKVLDVYEQWLGENQYFAGDEFSLADLVHMPNTDLLVRKTNKAGLFTERKNLAKWWDEVSARPSWKKVVELQNVPRPS* |
| OsGSTF5 | MAPMKVYGWVVSPWMARVLVALEEAGAEYEVVPMSRSGGDHRRPEHLARNPFGEIPVLEDGDLTLYQSRAIARYIFRKYKPEFLGLGEGGSLEESAMVDVWLDVEAHQHEAAVRPILWHCIINKFEGRDRDQGVVDESVRKLEKVLGVYEARLSGSRYLAGDRISLADLSHFSNMRYFMATEYAGVVDAYPHVKAWWEALLARPTVQKVMAGMPPDFGFGSGNIP* |
| OsGSTT1 | MQPLLKVYADRRSQPSRAIIIFCRVNRIDFEEVTVDLFKREHLSPEFKKINPMGQVPAIVDGRFRLFESHAILRYLATVFPGVADHWYPADLFTRAKLEAILDWHHSNLRRGAATFILNTVLAPSLGLPSSPQAAKEAEKVLFRSLGLIESMWLKGNAKFLLGNPQLSIADLSLVCEIMQLEVLGDSERDRILGPHEKIRSWVQNVKKATSPHFDEVHELIFKMKERMAAKRQSEPSKDLKTASKL* |
| OsGSTZ1 | MAAAEKTKPVLYSEWMSSCSYRVRIALNLKGIDYEYRAVTRGDPDYGKINPIKYVPALVDGDFTISDSLAIILYLEDKYPQHPLLPQDLKKKALNMQIANIVCSSIQPLQCYAVIGLADGKMSANESLQIVQHYTDKGFRAIEKLLEGCRSKYATGDEVQLADVFLAPQIHAGITRFQIDMSKYPILTRFYKAYMELPAFQAAVPENQPDAPSS* |
| OsGSTZ4 | MASSGSPEARQTHGEIAGAAAPERRLKLYSFWRSSCSYRVRIALSLKGLDYEYKPINLLANEQSHPEFEKLNPMKYVPALVDGDDTVVVDSFAILLYLEDTYPQHPLLPQDPKMKALNIQIASIVGSSIQPLQNNSVLDFIEEKLDSQEKVNWIQYHLNRGFTALEKMLKGCTTTYATGDEIQLGDLFLEPQIYGGIKRFGIDMTNYPTLARLHEAYMEHPAFQAALPERQPDAPSSPEI* |
| OsDHAR1 | MGVEVCVKAAVGHPDTLGDCPFSQRVLLTLEEKKVPYEMKLIDVQNKPDWFLKISPEGKVPVFNGGDGKWIPDSDVITQVIEEKYPTPSLVTPPEYASVGSKIFSCFTTFLKSKDPNDGSEKALLTELQALEEHLKAHGPFINGQNISAADLSLAPKLYHLQVALEHFKGWKIPEDLTNVHAYTEALFSRESFIKTKAAKEHLIAGWAPKVNA* |
| OsTCHQD1 | MQLYHHPYSLDSQKVRMALEEKGIDYTSYHVNPLTGKNMNVAFFRMNPSAKLPVFQNGAHVIYRAFDIIQYLDRLSVHLSGEIVPVNTEVYQWMQKVDSWNPKMFTLTHTPIKYRTFVSKFIRRVLIARMAEAPDLASMYHAKLREAYETEDKLKDPDIMKQSEEELSKLLDDVEAQLNNGKYLAGDEFSPADSVFIPILARITLLDLDEEYINCRPRLLEYYTLVKQRPSYKVAIGKFFGGWKKYRTLFKTSFFLCVRTLFRKY* |
| Csa1G024860 | MAEVKIIGSAGSLFCIRVEWALKLKGIEFEYIVEDLRNKSELLLKSNPVHKKIPVFLHNDKAISESLLIIEYIDETWKENPILPEDPYDRANARFWAKFLDEKGLIGAWEACQAEGEEKEKAVEAAIQNLALLDKEIQGKKFFGGEQIGYLDLAAGWICHWLNVLDEVGEMNVFDRERVPSLHEWAQNFIHVPVIKESLPPRETLVNYFKGSLSYVRSLAANKN* |
| Csa1G033120 | MADQNRVVLYGMWASPYAKRVQLALKIKGIPFQYVEEDLQNKSPDLLKFNPVYKKVPVLVHNGRPICESALILEYIEEVWNNNGPSLLPQDPYKRAQVRFWADYVQKQVFEGLFLLIKTEGEAQEKAVEDVKEKLKVLEEQGLKNLLAEGSTFVNGDELGYLDIVMLTLLGMYKVHEEFFGVKIVEEEKIPVVFSWLNRLIEHPFAKESAPPKEKVLGFLHFLRQKFIHSQAAA* |
| Csa1G033130 | MTDKNKVVLYGFWACPFVKSVELALKIKGIPFAYVEEDFLNKSPELLKFNPVYKKVPVLVHNERPICESAIILEYIEEVWNNNGPSLLPQDPFKRSQIRFWVDFVKNQLYDSLLLSMKTEGEAQEKAMEEVKERLKVVEEQGLKSLLAEGSPFVNGDELGYLDIGMLTILGRYKIYEEFFGMKIMEEEEIPIVFSWLNRLIEHPIAKELGAPPKEKILGLLHITRQRFLQSPVAA* |
| Csa1G033140 | MAEQNRVVLYGMWASPYAKRVQLALKIKGIPFQYVEEDLQNKSPDLLKFNPVYKKVPVLVHNGRPICESALILEYIEEVWNNNGPSLLPQDPYKRSQIRFWADYLQKQVFEGLFLLIKTEGEAQEKAIEDVKEKLKVLEEQGLKNLLAEGSTFVNGDELGYLDIGMLTILGRYKIYEEFFGMKIMEEEEIPIVFSWLNRLIEHPIAKEVTPPKEKVLGFLHFIRQKLLRSQAAA* |
| Csa1G033160 | MTEENKVVLYGLWASPFVKRVELALKIKAIPFEYVEEDFLNKSPELLKFNPVYKKVPVLVHNGRPICESAIIFEYIEEVWNNNGPPLLPQDPYKRSQIRFWADFVQNQLFDGLLLAMKTEGEAQEKAIKEVKEKLKVIEEQGLKSLLAEGSPFVNGDELGYLDIGMLTVLGRYKIYEEFFGMKIMEEEEIPIVFSWLNRLIEHPIAKEGAPPKEKVLGLLHIVRRRFLPSPVTA* |
| Csa1G033170 | MAEEHTVMLHGMWASPFVKGVELALKIKVITIESVEEDLQNKTPELLSFNPIYKNVSVLIHSGKPICESLVILENIN* |
| Csa1G701990 | MARSALDEMSTTGAFVRTASTFRNLISRDGSSQFPSESGRYHLYISYACPWASRCLAYLKLKGLEKAISFTSVKPIWERTKDSDDHMGWVFPSSDTEEPGAEPDPLNGVRSLRELYELASTNYSGKYTVPVLWDMKLKTIVNNESSEIIRMLNTEFNDIAENPHLDLYPTHLQAQIDETNDWIYRGINNGVYKCGFARQQQPYDQAVKELYEALDRCEEILSKQRYLCGNTLTEADIRLFVTIIRFDEVYAVHFKCNKKLLREYSNLFNYTKDIYQTKGVGSSVNMEHIKKHYYGSHPTINPFGIIPLGPNIDYSSPHDRDRFSS* |
| Csa2G336090 | MTSPVAASSLKLYSYWRSSCSHRVRIALNLKQYEKLNPIGYVPTLVDGDVVIADSFAIIMYLEEKYPQNPLLPCDLGKRAINYQAANIVSSSIQPLQNIAVLKYIEEKSGPAEKLRWVQHNIEKGFTALEKLLKPHAGKYATGDEIYMADLFLAPQIHGAINRFNIDMSKFSLLNRLNEEYNGTAAFQDAAPAEQPDATPTST* |
| Csa2G336130 | MAVDEESPLKLYSFWASTCAQRVRIALNLKGLNFQYKAVDILKGEHLAPEYLKLNPVGFVPTLVDGDVVIADSFAIIMYLEEKYPERPLLPTDLVKRAINHQVANIVSSSIQPLQNLIVEKYIEEKCGTEEKLSWVHMIIGKGFLALEKLLTVEAGNFATGDQIYMADLFLAPQLHRAIETFNLDMSKFPILSRLYEEYKKIAAFQDAAPENQPDAPSQN* |
| Csa2G348150 | MATPVKVYGPPLSTAVSRVLACLLEKDVQFQLLPVNMAKGEHKSQEYLKIQPFGQVPAFQDESISLFESRAICRYICDKHAEKGNKGLYGLDQLGKASIDQWIEAEGQSFNPASSILVFQLAFAPRMRLPQDEKAIKQNEEKLSKVLDVYEQRLGESRYLAGDEFSLADLSHLPNGHYLVNVADRAELFTSRKNVDRWWNEISGRESWKEVVELQTKASS* |
| Csa3G110620 | MALVLHSWKTNKNAYKALIAAEYNGVKVDLSQDFTMGVTNKSPEYLKMNPIGKVPVLETPDGAIFESNAIARYVARLKDSGLFGSSSIDCGHVEQWIDFSTFEIDIPVSTKLRPRFGYAAFHPGVEETADAALKRSFGALNSYLASNTFLVGHSVTLADIILTCNLYLGFNYILTKSFTSEFPHVERYFWTLVNQPNFKKIMGEVKQTDAVPPVKTPEEAAAAAKAKAEPKKQEEKPAAPAEEEAPKPKAKNPLDLLPPSSMILDEWKCLYSNTKSNFREVAIKGFWDMYDPEGYSLWFCDYKYNDENTVSFVTLNKVGGFLQRMDIARNVLDECYDMELYEWKKVDISDEAQKERVSQMIEDQEPFEGEALLDAKCFK* |
| Csa3G110630 | MALVLHSWTPSKNVYKVLIAAEYNGVKVDLAPDFTMGVSNKSPEFLKMNPIGKVPLLQTPDGPIFESNAITRYVARLKDSILFGSSPIDYGHVEQWIDFSSLEIDIHISTILAPRFGYGVYHAPAEEAANVALKRSFGALDSYLASNTFLVGHSVTLADIVLTCNLYYGFVYILPKSFTSAFPHVERYFWTLVNQPNFKKVIGEVKQTDAIPPVKTPEEVAAAAKAKAEPKKQEEKPAAPAEEAPKPKAKNPLDLLPPSSMILDEWKRLYSNTKTNFREVAIKGFWDMYDPEGYSLWFCDYKYNDENTVSFVTLNKVGGFLQRMDIARKYALGNMLVILSGLWLFRGKEIPQFVLDECYDMELYEWKKVDISDEAQKERVSQMIEDQEPFEGEALLDAKCFK* |
| Csa3G126140 | MDFQSHALKCSDDSSTSSDLVLYSFWRSSCSWRVRFALNLKGLSYEYRAVNLGTREQLSPDFESLNPLKYVPVLVDGPVVVSDSYAILLYLEEKYPQKALLPTDLRLKSRHLQVASIVSSSIQPLIMLELLKTIGENFGPEEPLPWAQSNLEKGFNALEKLVKDFSGQYALGDEVHMADVFLAPQISSAIENFGIDMYKFPTLARIYESYKTIPEFQASSPERQPDALH* |
| Csa3G126940 | MEEELKLFRTWSSPFPLRIVWALKLKGIEYETVYEDLANKSPLLLEYNPIHKKVPVLVHGGKPIAESLVILEYIEETWKQNPLLPQDPYQRAVARFWAKFGDDKVLESIKKVFMNQGKVREEGVEEAMENLKHLEEELKGKRFFGGEAIGFVDIAVGWLANIVSVMEEVVGLELITEERFPLLSKWTKEFAAAPIINENWPPRDKLITKYQALYQTYMTKQE* |
| Csa3G133350 | MEEVKLHGMWASPFVYRVKWGLELKGIPYEYVEEDIANKTSLLLHYNPIHKKVPVLVHGGKPVCESIIILEYIDEIWPQYPLFPVHPFDRAATRFWIKYADNKLFGSRLLFGSSSSEEEEKAKAEGLEFLRTIEEQCLGDKKFFAGEEIGALDLAFGGIAHWLPVLEQITGTKLLNADQFPRLYAWTQNFKESAVIRDNLPDAQKLAALYNALRHKQLRSAAE* |
| Csa3G133370 | MEELKLHGTWPSPFSYRVIWALALKGIPYQYIEEDLSNKSPLLLQYNPVHKKIPVLVHAGKPISESMIILEYIEQTWPQNPLLPSDPYERATARFWIKFIDDKGTVVFKIFRAKNEEEREDAKKQSLEMLRTVEEWGFSGGERKFFGGDDIGMADLAFGAIAYWLGMIEQVTGVKLLDVDEFPQVCGWIGRFKEAPVIRDNLPDWNPMMENFRRRREQLLESAKT* |
| Csa3G133380 | MTEVKLYGAWPSPFSYRVIWALALKGIPYEYVEEDLSNKSSLLLQYNPVHKKIPVFVHAGKPICESMIILQYIDETWPENPLLPSDPIQKAAARFWIKFVEDKSLVMWGLFIAEGEEDKEKARKATLEVLKTVEEQALGDKKFFGGHEINMVDLAYGFLARWLPVMEELVDVKVLEPNTNPRLYVWAENFRQAAVIRDNLPDHDEMVPRLRSRREQLLEMAKKS* |
| Csa3G133880 | MDEVKLHENWLSLSACKVIWALSLKDIPYRYEEEGITNKTPFLPYSNSVSVIVDGKSISDSSVILEYIDETWPQNPLLPSDLLDKATARFWIRFIDDKGAAITRMFYPGGKEQGAKVESLEMLRILEEEVIGEEKFFGGEKIGMVDLAFGEFVEWLGVFEEVIGEKLLESGSFPRLEAWSRAFMAVPAISENRADRNGMVENIKRFRIPLPQHRSALNSSNDMKNVLGFLELFRCFCELVSIVGRFF* |
| Csa3G133890 | MDEVKLYGHWSSPFVYRVIWALKIKAIPYEYVEEDIHNKSPFLLHYNPVYKRVPVLVHGGKSICESTVILEYIDETWPQNPLLPVDPLDRATARFWIQFVENMGLTTWTMFCSSGEEREKKRRESLEMLQIVEEKAIEDQKFFGGEKIGMVDLVYGVFGYWLGVIEDLIGEKLIETSTFPQLHARIKVFLQDPIIRENCPDRDRMAISFKAIRERLLGSR* |
| Csa3G609270 | MPTASASIFFPSPPPYPPPAILTKNPSRCHVIPRMSLNQSPSTGPKRSSSASSSSSSLLASITNLLWGPSLPPGLLIATVRTAWETTWQLMMAQLAPSDTSGTYTRPISQFRATKISANNLHLYVGLPCPWAHRTLIVRALKGLENAVPVSIAGPGSDGSWEFREKPKAENETLNPGLDKANGCRTLKEVYRMKRGGYNGRSTVPMLWDAEKKEVLCNESFDIIEIFNSGLNELAENPELDLSPPLLKRKIEEWNSIIYPNVNNGVYRCGFAQSQKAYDKAVEDLFSTLDLLDDHLGHSRYLCGENLTLADVCLFTTLIRFDLVYNVLFKCTKKKLLEYDNLHGYMRDIYQIPKVSATCNFTAIMDGYYKTLFPLNPGSIRPTIPTSCYHEALSVPSERRSLPFLGRIEQVSA* |
| Csa3M736660 | MAALVEEILPPSLDATAEQPPLFDGTTRLYTAYICPYAQRVWITRNYKGLQDKIKLVPLNLFNRPDWYKEKVYSPNKVPSLEHNGKVIGESLDLMKYVDSHFEGPSLLPNDPAKREYAEELLSYSDTFNGAMISSFKGDTAKEAGAQFDYLENALQKFDGPFLLGEISLVDIAYIPFVERFSVFLLEVFKIDITKGRPKLAAWIEEFNKIDAYKQTKADPKLVVEVYTKRFLG* |
| Csa3G736670 | MASADVQEIPPPSLDATAEQPPLFDGTTRLYTAYICPYAHRAWIIRNYKGLQHKIKLVPLDLRNRPDWYKEKVYPTNKVPSLEHNGKVIGESLDLLKYIDSNFEGPSLLPDDPGKREFAEELLSYSETFNGAIIHSFKGDTAKEAGAQFDYLENALGQFDGPFLVGEISQVDIAYIPFVERFHIFLLEALNIDITEGRPKLVAWIEEFNKMDAYKQTKVDPKLVVDIYTMVFLDSKYD* |
| Csa4G048540 | MALKVYADRMSQPSRAVLIFCKVNGIAFEEVKVTLSKFEHLTPEFKEINPMGKVPVIVDGRFKLFESHAILIYISCAFPGVADHWYPADLFRRAKIHSVLDWHHSNLRSGAAPLIFNTVLAPLFGRPLNPQAAAEAEKLLSKSLSKIESFWLKGNGKYLLGGLKPSIADLSLVCEIMELELLDEKDRSRILGPHPKVREWIENTRNATNPHFDEFHKILFKLKETLQKQHSLKLTSKI* |
| Csa4G064110 | MGEEVKLLDFWPSMFGMRVKIALAEKGVAYEYVEQDLRNKSPLLLQMNPIHKQIPVLIHNGKPICKSSIIVEYIDEFWKDKASLLPSDPYERAQARFWVDFIDKKIYPIGSKLWNRHKTEEEKEAAAKELLECFKQLQEELGDKDLFWWQNIWVYRYCSHSILQHVSCIQIAWKIGLRS* |
| Csa4G064120 | MADQVKLLDFWPSMFGMRVRVALALKCVPYEYVDEDLTNKSPLLLQMNPIHKKIPVLIHNGKPICESSIIVQYIDEFWKDKAPLFPTHPYDRAQARFWVDFIDKKLFDCGRKIWASKGEEQEEGKTEVIGILKQLEEVIGEKAYFGGECLGFVDIVLIGYYTWFYAYETIGNFSIESECPKIMSWAKRCLQNESVSESLPDPKKVCDFVLQMKKKYGL* |
| Csa4G064630 | MGDEVKILDFWPSMFGMRVRVALAEKGVGYECIEEDLRNKSPLLLEMNPIHKKIPVLIHNGKPICESSIIVEYIDEFWNDRAPLFPSDPYERAQARFWIDFIDKKLYGSVRKIYGGKGEEQEEGKKEMMEILKELEKVLGEKDYFGGECFGVLDIALIGFSTWFSGLESIGNFSIESECPKLISWAKRCLQRESVSKSLPDSKKTTEFIAELRKMIVE* |
| Csa4G064640 | MGEEVKLLDFWGSMFGMRIRIALAEKGVSYEYVEQDLRNKSHLLLQMNPIHKKVPVFIHNGKPICESYIILQYIDEVWKDKAPNLLPLHPYDRAQAKFWVDFIDKKLYDGAKKIWAGKGEEREKGKKEVIEILKQLEQVLGDKTYFGGESLGFVDIGLIGYYTWFYTYETIGNFSIESECPKIMCWVKRCMQNESVAKSLPEPKNVYEFVVQWKKSFGLD* |
| Csa4G064650 | MADQEVKLLDFWPSMFGMRVRIALAQKGVAYEYVEEDLRNKSPLLLEMNPIHKKIPVLIHNGKPICESSIIVEYIDEFWNDKAPLLPSHPYDRAQARFWVDFIDKKLYGPTRKIWATKGEEHEAGKKEFIEILKQLEQVLGEKEYFGGESLGFIDIALIGFYSWFYTYETVGKFSIEAECPKIISWGKRCLQNESVAKSLPDSKKIYDFVVQVQKALGII* |
| Csa4G303130 | MEGGVEVFGFWPSPFSLRVELALKLKGIQYQYVEEDVLNQKSDLLVKYNPIYKKVPVLVHHGKPISESLVILEYIEETWKHSPILPQDCHEKALARFWAAYIDGKVVSALGKVCGSKGEVIEEAVEEAKEALEPLEKELKSNKFFGGEKIGFLDIVGILIAFWIPASEEALGIEMLTAHKFPNLIQWIEELANDNVVKELIPKKDDLVTHMKTVLGKN* |
| Csa4G303140 | MQVEKSPPRRHIDLRKIPPGGIRTKELELRMRAGKGERRAVAETCEALKTLKEELKVKRFFGGETLGFVDIVTNFIAYWIPAIEESLGLDGLLSTEMERLPNQRRWCNEFEEHAIVKVIPPPKQDLLAFFISQFGTNVASK* |
| Csa4G303150 | MPAVLRVARSQEDEREKGIEEAEETLEPLEKELNIVGLILAGWIPATEEAIGFELLSAHKFPNLTKWSQHFVNHSVAKEVLPEKNFLVNFLKNVTFRPKNN* |
| Csa4G303160 | MAGEEEVKVFGFRSGSYSRRVELAFKLKGVDYEHIDEDLLNNNKSDLLLKYNPIYQKVPVLVHNGKPISESRVILEYIDETWTQNYAILPTDPYERALARFWAKYIDEKVVPAIEKAFRSNEEEREKRIEEAQEMLEPLEKELQNKLFFGGDKIGFVDIVGLVIPGWMHASQEAAGFEVLTIHKFPNLNRWIQDYLNHSVAKEVLPQKDFLVAFLSKVVFGRNN* |
| Csa4G303170 | MAGEDQEVQVFGFRSGPYSLRVELALKLKGVVYEHIDEDLVNKKSDLLVKYNPIYKKVPVLVHHGKPISESLVILEYIDETWTENYAILPQDPYQRALARFWAKYIDDKVVPAAQKVARSQEDEREKSIEDAQATLEPLEKELENKSFFGGDKIGFVDIVGLVLARWVPATEEAVGFELLSAHKFPNLTKWSQNFVNHSVAKEVLPKKDVLVAFLKNVVFSTKN* |
| Csa4G303680 | MAMAEEVELFGGWMSPFSRRVELGLKLKGIDYKYHEEDLKNKSDFLLTYNPIYKKVPVFLHNGNPISESLIILEYIDQVWNSLYPFFPQQNPYETAQARFWANYIDDKVLAATLKAAKSSKREEREKGLEETEETLKPLEKELQNKKFFGGNKIGIVDIVGTVIAYWIPAIEEAFGFELLTTKKFPKLTKWSEEIVNNSVVKQVLPPKSNLVAYLQTVLTTK* |
| Csa4G303690 | MAEQVQVLGFWESAFSRRVELALKLKGVEYQYFEEDLPHNKSDLLLKYNPIHKKVPVLLHHGRPIAESLVILEYIDDIWKENYPILPQHPHQRALARFWAKYIDDKVVGAVVKAAGSRGEDREKAIEEAREALEALEKEIEKNNELFGGDDIGFVDIVGTVIAGWVPAIEECFEFQLLTTDNFPNLIKWSDQLVQHSIVNQILPPKNEIVAFMKANWKF* |
| Csa4G304240 | MGEKVEVFGAWFSPFSRRVELALKLKGIQYDYIEEEIYKKKSDLILKFNPVYKKVPVFVHGGKPIAESIVILQYIDESWKDNPILPQHPYHKALALFWAKFLDDKVLPSLMKARRSGEGKDREEAIGEAREGLRALEEELKGKKFFGGEKLGFVDIVANFIAYWSQAMDEAFGVQILTSELQNLPRLTQWCHHFLQHPIVKHNLPPKTQLLALFKSQFVDTN* |
| Csa4G304250 | MAEQVQVLGFWGSPFSRRVELALKLKGVEYQYFEEDLFHNKSDLLLKYNPIHKKVPVLLHHGRPIAESLVILEYIDDIWKENYPILPQHPHQRALARFWAKFIDDKVVGAVVKAAGSRGEDREKAIEEAREALEALEKEIEKNNELFGGNDIGFVDIVGTVIAGWIPAFEECFGFQLLTADNFPNLIKWSDQLVNHSIFNQILPPKYEIVACMKANWKI* |
| Csa4G304750t | MAMGEEVELFGGWMSPFSRRVELGLKLKGIDYKYHEEDLKNKSDFLLTYNPIYKKVPVFLHNGNPISESLIILEYIDQVWNSLYPFFPQQNPYETAQARFWANYIDDKKELQNKKFFGGNKIGIVDIVGTVIAYWIPAIEEAFGFELLTTKKFPKLTKWSEEIVNNSVVKQVLPPKSKLVAYFELQVVSITN* |
| Csa4G304760 | MGEEVELFGGWMSPFSRRVELGLKLKGIDYKYHEEDLKNKSDFLLTYNPIYKKVPVFLHNGNPISESLIILEYIDQVWNSLYPFFPQQNPYETAQARFWAKYIDDKVLAATLKAAKSSKREEKEKGLEETEETLKPLEKELQNKKFFGGNKIGIVDIVGTVIAYWIPAIEEGFGSELLTSKKFPKLTKWSEEIVKNSVVKQVLPPKSVLVAYLQVVLTAN* |
| Csa5G331010 | MFSLSRINKFKICFLLGKVFDVGSKIWKSNGEEQEEAKKQLIEILKQLEEALGDKDFFGGEKFGIVDVILIPFTSWFYTYEKVGNFKVEECCPKFSAWEKRCLQKDTVASVLPCPEKIYNFITSMRKNLGLE* |
| Csa5G177050 | MIMATSTLGSTIGFSYSSSSSDISAYCILSSTTRFSFSSIIPSPLKLQKQPPGRRARALIAVKMAAQAFQEVLPPALTSVSEPPPIFDGTTRLYISYTCPYAQRVWITRNCKGLQNRIQLVPINLQDRPSWYKEKVYPPNKVPALEHNNEVKGESLDLIKYIDSNFEGPSLFPDEPEKREFAEELINYVNSFTGSVVSSFKGDGNEADATFDYIESALSKYGDGPFFLGQFSLVDIAYAPFIERFRPFLLEVKTYDITAGRPKLAAWIEETNMIEGYRQTRRDPQEHVDSYKKRFLSRL* |
| Csa7G395800 | MASIKVHGSPISTATARVLVCLYEKDLDYEFVLVDMKNNEHKKHPFLSINPFGQVPGFQDGDLTIFESRAITQYLAKTYANKGNQLIPNDPKKAAIVSTWIEVEGHHYDPPGVALSFEHLVKPLFGLGETDPAAVAENEAKLGKVLDIYENQLSKNKFLAGDEFSLADLHHLPNVSYLLATPSKKLFESRPRVNAWVTEIIARPSWAKVVALRNQS* |
| Csa7G395810 | MASIKVYGLALSTPTCKVLACLYEKDLEFEFINVKLHEGEHKKHPFLSINPFGQIPGFQDGDLTLFESRAITQYISANYTNNGTQLIPQDCKEAAALLTWIEVESHHFNPPASKLVYEIYLKPKLGWGETDGAVVEEKEAELAKVVDIYEKRLAESKYLAGEAFSLADLHHIPTLGFLLETQTKKLFEARPHVNAWVADIMARPAWAKVLALRK* |
| Csa7G395820 | MAGAIKVHGIPTSTATGRVLAALYEKDLHFELVNVKLHEGEHKREPFISLNPFGQVPAFQDGDLNLFESRAITQYISGNYASNGTQLIPQDPKKAAVVLTWIEVESHHFDPVAMKLVFELCLKPVFGWGDADPAVVEQSETELGKVLDIYEKRLTESKYLGGESFSLADLHHLPVLGYLLATQSKKLFESRPHVNAWVADIIARPSWAKVVELRK* |

**Supplementary Table S2** Primer sequence used for real time and RT-PCR amplification of *GST* genes of *C. maxima*

| **Gene name** | Forward | Reverse |
| --- | --- | --- |
| CmaGSTU1 | GCAGAGCAAAACAGAGTGGT | CTGGGCTTTTGTTCTGGATA |
| CmaGSTU2 | TGGCTCAGTGTTCTTGATGA | GGTGGAAGGGATTCTTTGAT |
| CmaGSTU3 | TGAAGCATTTGGAAGGAGAG | ATTTATGATAGGCGCAGCAG |
| CmaGSTU4 | AAGCTGTTCAGAACGTGGTC | AAAGCGACGAACTCTTGTTG |
| CmaGSTU5 | GGAAGGGAAGTTGAAAGAGG | CCAATCACTTCCTCCAACAC |
| CmaGSTU6 | ATGCCAAGAAGCAGAGCTTA | CGTCATTCCGATCTCATCTC |
| CmaGSTU7 | ACTGCAGACGTTGGAAGAAC | ACAACCTCTTCGATCACTGG |
| CmaGSTU8 | GATAAAGAGGAGGCGAGGAG | TCTTCCAAAACCTTCATCCA |
| CmaGSTU9 | AAGCCGTTCCCTACGAATAC | TGGATTCACAGATGGGTTTT |
| CmaGSTU10 | ACAAACCCATTATTGCCTCA | TCTCTTCTCCTTTGCTTCCA |
| CmaGSTU11 | AAAGGCGTGGATTATGAACA | AATGACCACGGACTCTGAAA |
| CmaGSTU12 | GGAGCTGATTGGAGTTCTGA | CAACTTACCAAACGCCTCAT |
| CmaGSTU13 | GCTCCTCTGTTACCGTCTGA | AGTAAGCCTTCTCCCCAAGA |
| CmaGSTU14 | CGAAGTGAAACTGCTGGATT | AGGGCTCTTGTTCCTCAGAT |
| CmaGSTU15 | GTTAGAATGGCTCTGGCTGA | ATTCACTGATGGGTTTTCCA |
| CmaGSTU16 | CACTCTGCAGGAGGTCTTGT | GATGCTTCAATGCCATTACC |
| CmaGSTU17 | ACAAAAAGGTGTTCGAGACG | CTTCCTCCAATTGCTTCAAA |
| CmaGSTU18 | AAACTGCGAGACAAAACAGG | TAGACTCGAGAATGGGCTTG |
| CmaGSTF1 | GCGCTGTCGTACGAGTATCT | GTAGTGCACATTGGGGATGT |
| CmaGSTF2 | GGGCAATCACGCAGTATATC | TGGATCATAATGGTGGCTCT |
| CmaGSTF3 | GCTTGATGTGTACGAGCAGA | ACCACCTCCTTCCAAGATTC |
| CmaGSTZ1 | CGAATTGCCCTAAACTTGAA | TATCCCCATCCACGAGAGTA |
| CmaGSTZ2 | GGAGAGCATTTGAATCCTGA | AAGATCACTCGGCAACAGAG |
| CmaGSTZ3 | AATATCCACAGAAGGCGTTG | AGTTTTCAGCAATCCCAACA |
| CmaEF1G1 | GATTTTGTCATGGGCGTAAC | AAACTGCTGTCCTTCAAACG |
| CmaEF1G2 | TGACTTTGCCACACTTGAAA | GTTCAGAGCAGCAAAAGAGC |
| CmaEF1G3 | CAAAAAGGAAGCTGCAAAAG | CATCTTACTGGGAGGAAGCA |
| CmaGHR1 | CGTTACCTCTGTGGGAACAC | ACAGAGCTGCTTACCCCTTT |
| CmaGHR2 | AGGGGAAGATTGAAGAATGG | AGCAAACATCAGCCAGAGTC |
| CmaGSTL1 | TCGTTTAAAGGCGATACTGC | TAGGCCTTCCTTCTGTGATG |
| CmaGSTT1 | ATCGAATGTCACAGCCATCT | CTCTTTGAATTCGGGAGACA |
| CmaGSTT2 | GTGCTGATCTTCTGCAAGGT | CCATTGGGTTGATCTCTTTG |
| Actin | GATTTGCTGGAGATGATGCT | TCACCAACATAGGCATCCTT |

**Supplementary Table S3** Putative cis-elements, more than 4 bp, were identified in 32 *CmaGST* genes in *Cucurbita maxima* using Place Web Signal Scan.

| **Gene name** | ***Cis* element** | **Organism** | **Sequence** | **Function** |
| --- | --- | --- | --- | --- |
| *CmaGSTU1* | ACGTATERD1 | *Arabidopsis thaliana* | ACGT | Induction by dehydration stress and dark-induced senescence. |
|  | BIHD1OS | *Oryza sativa* | TGTCA | Disease resistance responses |
|  | MYB2CONSENSUSAT | *A. thaliana* | YAACKG | Dehydration-responsive |
|  | GAREAT | *A. thaliana* | TAACAAR | Gibberellin biosynthesis during seed germination |
|  | HEXMOTIFTAH3H4 | *O. sativa* | ACGTCA | Cold Signaling |
|  | MYB1AT | *A. thaliana* | WAACCA | Abscisic acid signaling |
|  | MYB2AT | *A. thaliana* | TAACTG | Induced by dehydration stress |
|  | MYBCORE | *A. thaliana* | CNGTTR | Induced by dehydration stress |
|  | MYCATRD22 | *A. thaliana* | CACATG | Drought- and abscisic acid-regulated gene expression. |
|  | MYCCONSENSUSAT | *A. thaliana* | CANNTG | Regulator of cold-induced transcriptome and freezing tolerance |
|  | WBOXATNPR1 | *A. thaliana* | TTGAC | response to environmental stresses/ regulated plant defense response |
|  | CRTDREHVCBF2 | *Hordeum vulgare* | GTCGAC | DNA binding is regulated by temperature |
| *CmaGSTU2* | CATATGGMSAUR | *A. thaliana* | CATATG | Regulation through the drought-responsive element in an ABA-dependent pathway |
|  | CRTDREHVCBF2 | *Hordeum vulgare* | GTCGAC | DNA binding is regulated by temperature |
|  | BIHD1OS | *O. sativa* | TGTCA | Disease resistance responses |
|  | DRE2COREZMRAB17 | *O. sativa* | ACCGAC | function in drought-, high-salt- and cold-responsive gene expression |
|  | ELRECOREPCRP1 | *Nicotia tabacum* | TTGACC | Rathogen- and salicylic acid-induced genes |
|  | GCCCORE | *Solanum lycopersicum* | GCCGCC | Regulates defence-related gene expression |
|  | LTRECOREATCOR15 | *A. thaliana* | CCGAC | Low temperature responsive element |
|  | ARFAT | *A. thaliana* | TGTCTC | Responsive to ABA and auxin |
|  | MYCCONSENSUSAT | *A. thaliana* | CANNTG | Regulator of cold-induced transcriptome and freezing tolerance |
| *CmaGSTU3* | ACGTATERD1 | *A. thaliana* | ACGT | Induction by dehydration stress and dark-induced senescence. |
|  | ACGTABREMOTIFA2 | *A. thaliana* | ACGTGKC | Response to dehydration and high-salinity stresses. |
|  | ACGTABREMOTIFA | *A. thaliana* | TACGTGTC | Abscisic acid response element |
|  | ARFAT | *A. thaliana* | TGTCTC | Responsive to ABA and auxin |
|  | BIHD1OS | *O. sativa* | TGTCA | Disease resistance responses |
|  | LTRECOREATCOR15 | *A. thaliana* | CCGAC | Low temperature responsive element |
|  | LTRE1HVBLT49 | *H.vulgare* | CCGAAA | low-temperature-responsive element |
|  | CCAATBOX1 | *N. tabacum* | CCAAT | Chimaeric heat shock genes |
|  | ELRECOREPCRP1 | *N. tabacum* | TTGACC | Pathogen- and salicylic acid-induced genes |
|  | HEXMOTIFTAH3H4 | *O. sativa* | ACGTCA | Cold Signaling |
|  | MYBCORE | *A. thaliana* | CNGTTR | Induced by dehydration stress |
|  | MYCCONSENSUSAT | *A. thaliana* | CANNTG | Regulator of cold-induced transcriptome and freezing  tolerance |
|  | WBOXATNPR1 | *A. thaliana* | TTGAC | Functions in response to environmental stresses/ regulated plant defense response |
| *CmaGSTU4* | ACGTATERD1 | *A. thaliana* | ACGT | Induction by dehydration stress and dark-induced senescence. |
|  | DRE2COREZMRAB17 | *O. sativa* | ACCGAC | function in drought-, high-salt- and cold-responsive gene expression |
|  | BIHD1OS | *Oryza sativa* | TGTCA | Disease resistance responses |
|  | CCAATBOX1 | *N. tabacum* | CCAAT | Chimaeric heat shock genes |
|  | DPBFCOREDCDC3 | *A. thaliana* | ACACNNG | Abscisic acid response |
|  | IBOXCORE | Monocot plants | GATAA | Light-regulated |
|  | MYB1AT | *A. thaliana* | WAACCA | Abscisic acid signaling |
|  | MYBCORE | *A. thaliana* | CNGTTR | Induced by dehydration stress |
|  | MYCCONSENSUSAT | *A. thaliana* | CANNTG | Regulator of cold-induced transcriptome and freezing tolerance |
|  | WBOXATNPR1 | *A. thaliana* | TTGAC | response to environmental stresses/ regulated plant defense response |
| *CmaGSTU5* | ACGTATERD1 | *A. thaliana* | ACGT | Induction by dehydration stress and dark-induced senescence. |
|  | BIHD1OS | *O. sativa* | TGTCA | Disease resistance responses |
|  | GATABOX | *B. oleracea var. botrytis* | GATA | mosaic virus responsive |
|  | CCAATBOX1 | *N. tabacum* | CCAAT | Chimaeric heat shock genes |
|  | LTRE1HVBLT49 | *H.vulgare* | CCGAAA | low-temperature-responsive element |
|  | IBOXCORE | Monocot plants | GATAA | Light-regulated |
|  | CRTDREHVCBF2 | *H.vulgare* | GTCGAC | DNA binding is regulated by temperature |
|  | MYBCORE | *A. thaliana* | CNGTTR | Induced by dehydration stress |
|  | MYCCONSENSUSAT | *A. thaliana* | CANNTG | Regulator of cold-induced transcriptome and freezing tolerance |
|  | OSE1ROOTNODULE | *V. faba* | AAAGAT | Activation in the infected cells of root nodules |
|  | WBOXATNPR1 | *A. thaliana* | TTGAC | Functions in response to environmental stresses/ regulated plant defense response |
| *CmaGSTU6* | ACGTATERD1 | *A. thaliana* | ACGT | Induction by dehydration stress and dark-induced senescence. |
|  | BIHD1OS | *O. sativa* | TGTCA | Disease resistance responses |
|  | CCAATBOX1 | *N. tabacum* | CCAAT | Chimaeric heat shock genes |
|  | ELRECOREPCRP1 | *N. tabacum* | TTGACC | Pathogen- and salicylic acid-induced genes |
|  | LTRECOREATCOR15 | *A. thaliana* | CCGAC | Cold, and drought regulated gene expression. |
|  | IBOXCORE | Monocot plants | GATAA | Light-regulated |
|  | L1BOXATPDF1 | *Gossypium hirsutum* | TAAATGYA | Plant trichome development |
|  | LTRE1HVBLT49 | *H. vulgare* | CCGAAA | Low-temperature-responsive gene |
|  | MYBCORE | *A. thaliana* | CNGTTR | Induced by dehydration stress |
|  | NTBBF1ARROLB | *B. napus* | ACTTTA | Tissue-specific and auxin-regulated expression |
|  | WBOXATNPR1 | *A. thaliana* | TTGAC | Functions in response to environmental stresses/ regulated plant defense response |
| *CmaGSTU7* | ACGTATERD1 | *A. thaliana* | ACGT | Induction by dehydration stress and dark-induced senescence. |
|  | BIHD1OS | *O. sativa* | TGTCA | Disease resistance responses |
|  | CCAATBOX1 | *N. tabacum* | CCAAT | Chimaeric heat shock genes |
|  | LTRE1HVBLT49 | *Hordeum vulgare* | CCGAAA | low-temperature-responsive element |
|  | LTRECOREATCOR15 | *A. thaliana* | CCGAC | Cold, and drought regulated gene expression. |
|  | DRECRTCOREAT | *O. sativa* | RCCGAC | Function in drought-, high-salt- and cold-responsive gene expression |
|  | IBOXCORE | Monocot plant | GATAA | Light-regulated |
|  | MYBCORE | *A. thaliana* | CNGTTR | Induced by dehydration stress |
|  | MYCCONSENSUSAT | *A. thaliana* | CANNTG | Regulator of cold-induced transcriptome and freezing tolerance |
|  | WBOXATNPR1 | *A. thaliana* | TTGAC | Functions in response to environmental stresses/ regulated plant defense response |
| *CmaGSTU8* | ARFAT | *A. thaliana* | TGTCTC | Responsive to ABA and auxin |
|  | BIHD1OS | *O. sativa* | TGTCA | Disease resistance responses |
|  | GATABOX | *B. oleracea var. botrytis* | GATA | Mosaic virus responsive |
|  | LTRECOREATCOR15 | *A. thaliana* | CCGAC | Low temperature responsive element |
|  | MYCCONSENSUSAT | *A. thaliana* | CANNTG | Regulator of cold-induced transcriptome and freezing tolerance |
|  | WBOXATNPR1 | *A. thaliana* | TTGAC | response to environmental stresses/ regulated plant defense response |
| *CmaGSTU9* | ACGTATERD1 | *A. thaliana* | ACGT | Induction by dehydration stress and dark-induced senescence. |
|  | LTRECOREATCOR15 | *A. thaliana* | CCGAC | Low temperature responsive element |
|  | MYCCONSENSUSAT | *A. thaliana* | CANNTG | Regulator of cold-induced transcriptome and freezing tolerance |
|  | WBOXATNPR1 | *A. thaliana* | TTGAC | response to environmental stresses/ regulated plant defense response |
|  | MYBCORE | *A. thaliana* | CNGTTR | Induced by dehydration stress |
|  | LTRE1HVBLT49 | *H. vulgare* | CCGAAA | low-temperature-responsive element |
|  | WBOXATNPR1 | *A. thaliana* | TTGAC | Functions in response to environmental stresses and regulated plant defense response |
| *CmaGSTU10* | ABRELATERD1 | *A. thaliana* | ACGTG | Dehydration stress and dark-induced senescence |
|  | DRE2COREZMRAB17 | *O. sativa* | ACCGAC | function in drought-, high-salt- and cold-responsive gene expression |
|  | LTRECOREATCOR15 | *A. thaliana* | CCGAC | Low temperature responsive element |
|  | MYCCONSENSUSAT | *A. thaliana* | CANNTG | Regulator of cold-induced transcriptome and freezing tolerance |
|  | WBOXATNPR1 | *A. thaliana* | TTGAC | response to environmental stresses/ regulated plant defense response |
|  | MYBCORE | *A. thaliana* | CNGTTR | Induced by dehydration stress |
|  | LTRE1HVBLT49 | *H. vulgare* | CCGAAA | low-temperature-responsive element |
| *CmaGSTU11* | ACGTATERD1 | *A. thaliana* | ACGT | Induction by dehydration stress and dark-induced senescence. |
|  | BIHD1OS | *O. sativa* | TGTCA | Disease resistance responses |
|  | LTRECOREATCOR15 | *A. thaliana* | CCGAC | Cold-, drought- and ABA regulated gene expression. |
|  | MYBCORE | *A. thaliana* | CNGTTR | Induced by dehydration stress |
|  | MYCCONSENSUSAT | *A. thaliana* | CANNTG | Regulator of cold-induced transcriptome and freezing tolerance |
|  | WBOXATNPR1 | *A. thaliana* | TTGAC | Response to environmental stresses/ regulated plant defense response |
| *CmaGSTU12* | BIHD1OS | *O. sativa* | TGTCA | Disease resistance responses |
|  | CCAATBOX1 | *N. tabacum* | CCAAT | Chimaeric heat shock genes |
|  | DRECRTCOREAT | *O. sativa* | RCCGAC | Function in drought-, high-salt- and cold-responsive gene expression |
|  | IBOXCORE | Monocot plants | GATAA | Light-regulated |
|  | LTRECOREATCOR15 | *A. thaliana* | CCGAC | Cold-, drought- and ABA regulated gene expression. |
|  | L1BOXATPDF1 | *G. hirsutum* | TAAATGYA | Plant trichome development |
|  | MYB1AT | *A. thaliana* | WAACCA | Abscisic acid signaling |
|  | MYCCONSENSUSAT | *A. thaliana* | CANNTG | Regulator of cold-induced transcriptome and freezing tolerance |
|  | WBOXATNPR1 | *A. thaliana* | TTGAC | Functions in response to environmental stresses/ regulated plant defense response |
| *CmaGSTU13* | ABRELATERD1 | *A. thaliana* | ACGTG | Dehydration stress and dark-induced senescence |
|  | ACGTATERD1 | *A. thaliana* | ACGT | Induction by dehydration stress and dark-induced senescence. |
|  | ABREATRD22 | *A. thaliana* | RYACGTGGYR | Dehydration is mediated by abscisic acid |
|  | CCAATBOX1 | *N. tabacum* | CCAAT | Chimaeric heat shock genes |
|  | GATABOX | *B. oleracea var. botrytis* | GATA | Mosaic virus responsive |
|  | GAREAT | *A. thaliana* | TAACAAR | Gibberellin biosynthesis during seed germination |
|  | IBOXCORE | Monocot plants | GATAA | Light-regulated |
|  | MYB2AT | *A. thaliana* | TAACTG | Induced by dehydration stress |
|  | MYCATRD22 | *A. thaliana* | CACATG | Drought- and abscisic acid-regulated gene expression. |
|  | MYB1LEPR | *S. lycopersicum* | GTTAGTT | Defence-related gene expression |
|  | MYBCORE | *A. thaliana* | CNGTTR | Induced by dehydration stress |
| *CmaGSTU14* | ACGTATERD1 | *A. thaliana* | ACGT | Induction by dehydration stress and dark-induced senescence. |
|  | ARFAT | *A. thaliana* | TGTCTC | Responsive to ABA and auxin |
|  | DRE2COREZMRAB17 | *O. sativa* | ACCGAC | function in drought-, high-salt- and cold-responsive gene expression |
|  | LTRECOREATCOR15 | *A. thaliana* | CCGAC | Low temperature responsive element |
|  | MYCCONSENSUSAT | *A. thaliana* | CANNTG | Regulator of cold-induced transcriptome and freezing tolerance |
|  | MYBCORE | *A. thaliana* | CNGTTR | Induced by dehydration stress |
|  | WBOXATNPR1 | *A. thaliana* | TTGAC | Response to environmental stresses/ regulated plant defense response |
| *CmaGSTU15* | ACGTATERD1 | *A. thaliana* | ACGT | Induction by dehydration stress and dark-induced senescence. |
|  | MYB2AT | *A. thaliana* | TAACTG | Induced by dehydration stress |
|  | MYCATRD22 | *A. thaliana* | CACATG | Drought- and abscisic acid-regulated gene expression. |
|  | MYB1LEPR | *S. lycopersicum* | GTTAGTT | Defence-related gene expression |
|  | MYBCORE | *A. thaliana* | CNGTTR | Induced by dehydration stress |
|  | WBOXATNPR1 | *A. thaliana* | TTGAC | Functions in response to environmental stresses/ regulated plant defense response |
| *CmaGSTU16* | ABRELATERD1 | *A. thaliana* | ACGTG | Dehydration stress and dark-induced senescence |
|  | ACGTATERD1 | *A. thaliana* | ACGT | Induction by dehydration stress and dark-induced senescence. |
|  | CCAATBOX1 | *N. tabacum* | CCAAT | Chimaeric heat shock genes |
|  | MYB2AT | *A. thaliana* | TAACTG | Induced by dehydration stress |
|  | MYB1LEPR | *S. lycopersicum* | GTTAGTT | Defence-related gene expression |
|  | OSE2ROOTNODULE | *V. faba* | AAAGAT | Activation in the infected cells of root nodules |
|  | WBOXATNPR1 | *A. thaliana* | TTGAC | Functions in response to environmental stresses/ regulated plant defense response |
| *CmaGSTU17* | BIHD1OS | *O. sativa* | TGTCA | Disease resistance responses |
|  | CCAATBOX1 | *N. tabacum* | CCAAT | Chimaeric heat shock genes |
|  | GATABOX | *B. oleracea var. botrytis* | GATA | Mosaic virus responsive |
|  | MYB2AT | *A. thaliana* | TAACTG | Induced by dehydration stress |
|  | MYBCORE | *A. thaliana* | CNGTTR | Induced by dehydration stress |
|  | MYCCONSENSUSAT | *A. thaliana* | CANNTG | Regulator of cold-induced transcriptome and freezing tolerance |
|  | OSE2ROOTNODULE | *V. faba* | AAAGAT | Activation in the infected cells of root nodules |
|  | HBOXCONSENSUSPVCHS | *N. tabacum* | CCTACCNNCT | Induced by wounding and by abiotic stress |
|  | WBOXATNPR1 | *A. thaliana* | TTGAC | Putative functions in response to environmental stresses/ regulated plant defense response |
| *CmaGSTU18* | ABRELATERD1 | *A. thaliana* | ACGTG | Dehydration stress and dark-induced senescence |
|  | BIHD1OS | *O. sativa* | TGTCA | Disease resistance responses |
|  | DRECRTCOREAT | *O. sativa* | RCCGAC | Function in drought-, high-salt- and cold-responsive gene expression |
|  | GAREAT | *A. thaliana* | TAACAAR | Gibberellin biosynthesis during seed germination |
|  | MYBCORE | *A. thaliana* | CNGTTR | Induced by dehydration stress |
|  | MYCCONSENSUSAT | *A. thaliana* | CANNTG | Regulator of cold-induced transcriptome and freezing tolerance |
|  | WBOXATNPR1 | *A. thaliana* | TTGAC | Functions in response to environmental stresses/ regulated plant defense response |
| *CmaGSTF1* | BIHD1OS | *O. sativa* | TGTCA | Disease resistance responses |
|  | ELRECOREPCRP1 | *N. tabacum* | TTGACC | Pathogen- and salicylic acid-induced genes |
|  | GAREAT | *A. thaliana* | TAACAAR | Gibberellin biosynthesis during seed germination |
|  | HBOXCONSENSUSPVCHS | *N. tabacum* | CCTACCNNCT | Induced by wounding and by abiotic stress |
|  | MYBCORE | *A. thaliana* | CNGTTR | Induced by dehydration stress |
|  | MYCCONSENSUSAT | *A. thaliana* | CANNTG | Regulator of cold-induced transcriptome and freezing tolerance |
|  | WBOXATNPR1 | *A. thaliana* | TTGAC | Rresponse to environmental stresses/ regulated plant defense response |
| *CmaGSTF2* | ABRELATERD1 | *A. thaliana* | ACGTG | Dehydration stress and dark-induced senescence |
|  | ELRECOREPCRP1 | *N. tabacum* | TTGACC | Pathogen- and salicylic acid-induced genes |
|  | GAREAT | *A. thaliana* | TAACAAR | Gibberellin biosynthesis during seed germination |
|  | MYCCONSENSUSAT | *A. thaliana* | CANNTG | Regulator of cold-induced transcriptome and freezing tolerance |
|  | MYBCORE | *A. thaliana* | CNGTTR | Induced by dehydration stress |
| *CmaGSTF3* | BIHD1OS | *O. sativa* | TGTCA | Disease resistance responses |
|  | ELRECOREPCRP1 | *N. tabacum* | TTGACC | Pathogen- and salicylic acid-induced genes |
|  | GAREAT | *A. thaliana* | TAACAAR | Gibberellin biosynthesis during seed germination |
|  | MYCCONSENSUSAT | *A. thaliana* | CANNTG | Regulator of cold-induced transcriptome and freezing tolerance |
| *CmaEF1G1* | ELRECOREPCRP1 | *N. tabacum* | TTGACC | Pathogen- and salicylic acid-induced genes |
|  | LTRECOREATCOR15 | *A. thaliana* | CCGAC | Low temperature responsive element |
|  | MYCCONSENSUSAT | *A. thaliana* | CANNTG | Regulator of cold-induced transcriptome and freezing tolerance |
|  | MYBCORE | *A. thaliana* | CNGTTR | Induced by dehydration stress |
| *CmaEF1G2* | DRE2COREZMRAB17 | *O. sativa* | ACCGAC | function in drought-, high-salt- and cold-responsive gene expression |
|  | LTRECOREATCOR15 | *A. thaliana* | CCGAC | Low temperature responsive element |
|  | MYCCONSENSUSAT | *A. thaliana* | CANNTG | Regulator of cold-induced transcriptome and freezing tolerance |
|  | MYBCORE | *A. thaliana* | CNGTTR | Induced by dehydration stress |
|  | WBOXATNPR1 | *A. thaliana* | TTGAC | Response to environmental stresses/ regulated plant defense response |
| *CmaEF1G3* | BIHD1OS | *O. sativa* | TGTCA | Disease resistance responses |
|  | MYCCONSENSUSAT | *A. thaliana* | CANNTG | Regulator of cold-induced transcriptome and freezing tolerance |
|  | WBOXATNPR1 | *A. thaliana* | TTGAC | Response to environmental stresses/ regulated plant defense response |
| *CmaGSTT1* | MYCCONSENSUSAT | *A. thaliana* | CANNTG | Regulator of cold-induced transcriptome and freezing tolerance |
|  | MYBCORE | *A. thaliana* | CNGTTR | Induced by dehydration stress |
|  | WBOXATNPR1 | *A. thaliana* | TTGAC | Response to environmental stresses/ regulated plant defense response |
|  | CRTDREHVCBF2 |  | *H.vulgare* |  |
| *CmaGSTT2* | DRE2COREZMRAB17 | *O. sativa* | ACCGAC | Function in drought-, high-salt- and cold-responsive gene expression |
|  | LTRECOREATCOR15 | *A. thaliana* | CCGAC | Low temperature responsive element |
|  | MYBCORE | *A. thaliana* | CNGTTR | Induced by dehydration stress |
|  | WBOXATNPR1 | *A. thaliana* | TTGAC | Response to environmental stresses/ regulated plant defense response |
|  | CRTDREHVCBF2 | *H.vulgare* | GTCGAC | DNA binding is regulated by temperature |
| *CmaGSTZ1* | MYCCONSENSUSAT | *A. thaliana* | CANNTG | Regulator of cold-induced transcriptome and freezing tolerance |
|  | MYBCORE | *A. thaliana* | CNGTTR | Induced by dehydration stress |
|  | WBOXATNPR1 | *A. thaliana* | TTGAC | Response to environmental stresses/ regulated plant defense response |
|  | LTRE1HVBLT49 | *A. thaliana* | CCGAAA | Low-temperature-responsive |
| *CmaGSTZ2* | LTRECOREATCOR15 | *A. thaliana* | CCGAC | Low temperature responsive element |
|  | MYCCONSENSUSAT | *A. thaliana* | CANNTG | Regulator of cold-induced transcriptome and freezing tolerance |
|  | MYBCORE | *A. thaliana* | CNGTTR | Induced by dehydration stress |
|  | WBOXATNPR1 | *A. thaliana* | TTGAC | Response to environmental stresses/ regulated plant defense response |
|  | CRTDREHVCBF2 | *H.vulgare* | GTCGAC | DNA binding is regulated by temperature |
| *CmaGSTZ3* | MYCCONSENSUSAT | *A. thaliana* | CANNTG | Regulator of cold-induced transcriptome and freezing tolerance |
|  | MYBCORE | *A. thaliana* | CNGTTR | Induced by dehydration stress |
|  | WBOXATNPR1 | *A. thaliana* | TTGAC | Response to environmental stresses/ regulated plant defense response |
|  | CRTDREHVCBF2 | *H.vulgare* | GTCGAC | DNA binding is regulated by temperature |
| *CmaGSTL1* | DRE2COREZMRAB17 | *O. sativa* | ACCGAC | Function in drought-, high-salt- and cold-responsive gene expression |
|  | BIHD1OS | *O. sativa* | TGTCA | Disease resistance responses |
|  | MYCCONSENSUSAT | *A. thaliana* | CANNTG | Regulator of cold-induced transcriptome and freezing tolerance |
|  | MYBCORE | *A. thaliana* | CNGTTR | Induced by dehydration stress |
|  | WBOXATNPR1 | *A. thaliana* | TTGAC | Response to environmental stresses/ regulated plant defense response |
| *CmaGHR1* | MYCCONSENSUSAT | *A. thaliana* | CANNTG | Regulator of cold-induced transcriptome and freezing tolerance |
|  | MYBCORE | *A. thaliana* | CNGTTR | Induced by dehydration stress |
|  | WBOXATNPR1 | *A. thaliana* | TTGAC | Response to environmental stresses/ regulated plant defense response |
|  | CRTDREHVCBF2 |  | *H.vulgare* | DNA binding is regulated by temperature |
| *CmaGHR1* | BIHD1OS | *O. sativa* | TGTCA | Disease resistance responses |
|  | MYCCONSENSUSAT | *A. thaliana* | CANNTG | Regulator of cold-induced transcriptome and freezing tolerance |
|  | MYBCORE | *A. thaliana* | CNGTTR | Induced by dehydration stress |
|  | WBOXATNPR1 | *A. thaliana* | TTGAC | Response to environmental stresses/ regulated plant defense response |
